# Effects of Yoga Nidra on Stress, Anxiety, and Depression: A Systematic Review and Meta‐Analysis

**DOI:** 10.1111/nyas.70149

**Published:** 2025-12-01

**Authors:** Shashank Ghai, Pawel Odyniec, Ishan Ghai

**Affiliations:** ^1^ Department of Political, Historical, Religious and Cultural Studies Karlstad University Karlstad Sweden; ^2^ Centre for Societal Risk Research Karlstad University Karlstad Sweden; ^3^ University of Freiburg Freiburg Germany

**Keywords:** holistic care, meditation, mental health, non‐sleep deep rest, Yoga Nidra

## Abstract

The global mental health crisis has escalated to unprecedented levels, with stress, anxiety, and depression posing major public health concerns. Conventional interventions have shown limited success in addressing these multifaceted issues, prompting researchers to explore alternative solutions. Yoga Nidra (YN), a meditative practice, has gained momentum over the past decade as a potential holistic approach to mental health care. Yet, its clinical effectiveness remains inadequately understood. This systematic review and meta‐analysis rigorously assessed YN's impact on stress, anxiety, and depression. A comprehensive search of seven databases and one trial database yielded 814 articles, of which 73 studies involving 5201 participants met the inclusion criteria. Between‐group meta‐analyses revealed significant benefits of YN for stress (Hedge's *g*: −0.80 with active comparator, −1.70 with no comparator), anxiety (active: −1.35, no comparator: −1.43), and depression (active: −0.69, no comparator: −0.92). Within‐group analyses supported these effects, reinforcing YN's therapeutic potential. However, given the low methodological quality and variability in intervention delivery, these moderate‐to‐large effects should be interpreted cautiously, as they likely reflect inflated estimates. Despite these limitations, YN shows potential in managing mental health symptoms, underscoring the need for high‐quality, standardized research to establish its efficacy as a viable clinical intervention.

## 1 Introduction

The global mental health crisis has reached unprecedented levels, placing a significant burden on societies worldwide [1]. Disorders such as anxiety and depression are now among the leading causes of disease burden [2]. Recent statistics show a sharp rise in disability‐adjusted life years over the past decades [3, 4]. Specifically, depression and anxiety rank as the second and sixth highest causes of years lived with disability and disability‐adjusted life years across the world, respectively [5]. While stress is not a disorder but a natural response to challenging situations [6], when experienced over a prolonged period of time or beyond an individual's coping capacity, it can transform into pathological stress [7], a condition that could contribute to the onset and worsening of anxiety and depression [8]. The prevalence of these mental health conditions is expected to further worsen across the globe due to a range of factors, including after pandemic effects [9], and even the ongoing political turmoil across the world [10].

The increasing prevalence of these disorders, despite advancements in pharmacotherapy, raises important questions about the sustained effectiveness of these interventions in managing such conditions [11]. Notably, the annual prescription rates of antidepressants and antianxiety medications have continued to rise over the past decade, particularly among vulnerable population groups [12, 13]. Moreover, concerns regarding stigmatization and the potential for addiction associated with the use of psychotherapeutic medications present a significant challenge [14]. These considerations have increasingly prompted researchers in modern medicine to explore alternative approaches, including ancient Eastern spiritual practices such as meditation and yoga, which may serve as a complementary strategy for managing these persistent conditions [15].

One such meditative practice that has been drawing widespread attention is Yoga Nidra (YN). While ancient Indian texts do not tie YN to a specific yogic discipline, and later medieval texts only loosely associate it with yogic absorption (samādhi), its detailed practice was not well documented until it was standardized by Swami Satyananda Saraswati at the Bihar School of Yoga in 1976 [16]. In the traditional yogic framework, YN is classified as a form of Raja Yoga, functioning specifically as a practical application of *pratyāhāra*, the fifth limb of Patanjali's Aṣṭāṅga Yoga, which centers on the deliberate withdrawal of sensory awareness from external stimuli [16, 17]. This sensory withdrawal is achieved through systematic guidance, wherein YN practitioners progressively detach from bodily sensations, breath patterns, and successive layers of conscious and unconscious experience, enabling awareness to redirect inward. As this withdrawal of the senses deepens and stabilizes, it organically establishes foundations for dhāraṇā (concentration) and prepares practitioners for more refined meditative states, progressively moving toward samādhi.

In practice, YN is typically led by an instructor, following a structured seven‐step process: preparation, saṅkalpa (resolve), rotation of consciousness, breath awareness, relaxation of feelings and emotions, visualization, and conclusion [16, 18]. During a session, practitioners typically lie in a comfortable supine position while following guided instructions, and unlike conventional sleep, YN induces a unique state of awareness between wakefulness and sleep that promotes deep relaxation [17]. Several mechanisms have been suggested in the literature by which YN may have beneficial effects on mental health, particularly in the regulation of stress, anxiety, and depression. For instance, as a meditative practice, YN incorporates aspects of focused attention meditative practices [19]. Steps such as rotation of consciousness, breath awareness, and visualization require practitioners to focus on specific body parts (e.g., performing a body scan), maintain awareness of their breathing, and actively visualize images to induce relaxation. Here, by focusing and sustaining attention on a particular aspect during YN, practitioners may become more adept at detecting and disengaging from distractions. Prolonged practice with focused attention could further enhance the automatic attentional regulation within cognitive systems responsible for conflict monitoring, selective attention, and sustained attention. This, in turn, may foster a state of “effortless concentration,” characterized by reduced activation in systems involved in regulating attention and optimizing performance [19]. Neuroimaging studies have shown that practicing YN can precisely suppress activity in neural networks responsible for executive control, while enhancing sensory experiences and relaxation [20, 21]. This effect may be linked to increased striatal dopamine release observed in YN practitioners [21], which could serve as a potential mechanism for replenishing neurotransmitters typically depleted in conditions such as stress [22], anxiety, and depression [23], thus aiding recovery.

Another key neurophysiological mechanism that may explain the efficacy of YN is its role as a focused attentional meditation that promotes emotional regulation [19]. This may occur particularly during the stage in YN where practitioners are encouraged to recall intense emotional feelings, experience them, and then ultimately discard them. A recent functional magnetic resonance imaging study corroborated these findings, reporting increased activation in regions such as the anterior cingulate cortex, limbic system, and insula among YN practitioners compared to controls [24]. Additionally, the mindful breath awareness practiced during YN might also contribute to reduced amygdala activation, as shown in another study [25]. The overall implication of these findings is that YN could help individuals not only regulate attention but also emotions, potentially leading to the modulation of the default mode network, an essential process for managing stress, anxiety, and depression [24].

In addition to the neurophysiological changes, several physiological benefits of practicing YN have been reported in the literature [17]. For instance, studies have shown that YN can have beneficial effects on the autonomic nervous system by reducing cortisol levels [26], blood pressure [27, 28], inflammatory markers [29], and respiratory rate [30], while enhancing heart rate variability [31]. These physiological parameters are important biomarkers of psychological health and affective disorders, highlighting how YN may play a role in enhancing recovery for individuals affected by stress, anxiety, and depression disorders [32, 33, 34]. Researchers have also adapted YN by removing its religious or mystical elements to make it more accessible in secular contexts. These adaptations include practices like Restorative Integration (iRest) and Non‐Sleep Deep Rest, which might facilitate its implementation and adoption as a therapeutic tool in modern society [35].

Despite growing evidence highlighting the effects of YN, the existing literature lacks consensus. Multiple clinical trials have evaluated YN's efficacy for stress, anxiety, and depression across diverse populations, but with inconsistent findings. While some trials report significant beneficial effects [36, 37, 38, 39], others show no substantial impact [30, 40−42], raising uncertainty about its therapeutic value. This lack of consensus warrants evidence‐based synthesis through systematic review and meta‐analysis. To date, only one systematic review has examined YN's effects on these conditions [43]. Although it reported positive effects, it had notable limitations, the most important being a restricted search carried out across just two databases, yielding only 16 studies and potentially overlooking relevant research. Moreover, the review also lacked a meta‐analysis, which is essential for quantitatively assessing YN's overall efficacy and informing clinical guidelines [44].

To address these gaps, the present review aims to conduct a comprehensive systematic review and meta‐analysis, considering both between‐group and within‐group perspectives. Another objective of this review was to examine variations in the delivery of YN, including the setting (e.g., in‐person or home‐based), instructor involvement, session structure, and the specific YN approach used. The goal was to help standardize YN protocols for future research and clinical applications by identifying best practices that enhance its effectiveness. A clearer understanding of these factors could improve intervention design, strengthen research validity, and support the integration of YN into mental health interventions for stress, anxiety, and depression.

## 2 Methods

The systematic review was performed as per the guidelines outlined in the Preferred Reporting Items for Systematic Reviews and Meta‐Analyses (PRISMA‐SR) statement [45]. The study was preregistered in PROSPERO (CRD42024617650). A PRISMA‐SR checklist has been provided in Table S1.

### 2.1 Data Sources

A search of literature was performed across seven academic databases (Pubmed, CINAHL, SPORTDiscus, PsychInfo, Web of Science, Scopus, ProQuest) and a trial database (Cochrane Central Register of Controlled Trials) from inception until February 2025. Although two reviewers (S.G. and I.G.) initially piloted a full PICOS‐based strategy, it yielded very few records. After further consultation with experienced librarians, the search strategy was refined to focus solely on the intervention of interest, YN, using a broad combination of keywords and synonyms (Table S2). By concentrating on YN and its associated key terms, we were able to capture all relevant literature regardless of study context, population, outcome, or setting, thereby ensuring the most comprehensive and replicable evidence base possible. Furthermore, to capture gray literature, we searched the National Digital Library of India and performed manual Google Scholar searches for each keyword associated with YN mentioned in Table S2, by screening up to the first 10 pages of results per term. Additionally, a search across the citation list of the included studies was also performed to find additional relevant literature. Our entire searches were restricted to English and Hindi, the languages in which our team has the proficiency required to accurately interpret findings and conduct rigorous methodological quality assessments.

### 2.2 Eligibility Criteria

The inclusion criteria for the selection of the study were developed according to the PICOS (population, intervention, comparator, outcome of interest, and study design) criteria by two reviewers (S.G. and I.G.). The criteria were as follows:

1. Population: Studies involving healthy individuals, as well as those with physical health conditions, mental health conditions, or neurological conditions.
2. Intervention: Studies assessing the impact of YN on stress, anxiety, and depression.
3. Comparator: Studies evaluating YN with or without a comparator.
4. Outcome: Studies assessing the impact of YN on stress‐, anxiety‐, and depression‐related outcomes.
5. Study Design: All types of qualitative and quantitative designs, including randomized controlled trials, nonrandomized controlled clinical trials, quasi‐experimental designs, crossover trials, cross‐sectional studies, cohort studies, feasibility studies, case series, and case studies.
6. Language: Studies published in English or Hindi languages.
7. Publication sources: Studies published in peer‐reviewed academic journals, as well as dissertations, and conference proceedings.

The title, abstract, and full‐text screening of the articles were conducted independently by two reviewers (S.G. and I.G.). Any disagreements regarding study selection were resolved through discussion. From the final list of selected studies, the authors extracted relevant data using a customized extraction sheet. The extracted information included author details, year of publication, country of research, study design, and participant demographics (sample size, sex, age, and health status). Additionally, details of the YN intervention (i.e., training dosage, intervention structure, presence of a trained instructor, and training setting) were extracted. Besides, scales used for outcome assessments, assessment time points, and overall study results were also extracted.

### 2.3 Quality Appraisal

Two independent reviewers (S.G. and I.G.) conducted quality assessments for all included studies using design‐specific methodological appraisal tools.

#### 2.3.1 Randomized Designs

We employed the Cochrane Risk of Bias 2 (RoB‐2) tool [46], which evaluates bias across five key domains: bias arising from the randomization process, bias due to deviations from intended interventions, bias due to missing outcome data, bias in measurement of the outcome, and bias in selection of the reported result. According to RoB‐2 guidance [47], overall risk‐of‐bias judgments were assigned as follows:

1. Low risk of bias: The trial was judged to be at low risk of bias for all domains.
2. Some concerns: The trial raised some concerns in at least one domain, but was not at high risk of bias for any domain.
3. High risk of bias: The trial was judged to be at high risk of bias in at least one domain.

Results were visualized using the online robvis tool [48].

#### 2.3.1 Nonrandomized Controlled and Noncontrolled Designs

We used the modified Downs and Black scale [49], which is a 28‐point instrument that assesses methodological quality across five domains:

1. Reporting (11 points) evaluates clarity and completeness in describing the study hypothesis, main outcomes, participant characteristics, intervention details, distributions of principal confounders, main findings, estimates of random variability, adverse events, characteristics of patients lost to follow‐up, and actual probability values.
2. External validity (3 points) assesses the representativeness of participants and settings, including whether invited participants reflect the source population, whether those who agreed to participate are representative of the invited sample, and whether staff, places, and facilities mirror usual treatment settings.
3. Study bias (7 points) examines measures to minimize bias, including attempts to blind subjects and assessors, transparency about data dredging, appropriateness of statistical tests, adjustments for differing follow‐up times, intervention compliance, and validity and reliability of outcome measures.
4. Confounding and selection bias (6 points) evaluates group comparability and handling of confounding factors, including whether participants were recruited from the same population and time period, randomization procedures, allocation concealment, adjustment for confounders, and accounting for losses to follow‐up.
5. Power (1 point) determines whether the study had sufficient statistical power to detect a clinically important effect.

Studies were classified according to their total score: excellent (24−28), good (19−23), fair (14−18), or poor (<14) [49].

#### 2.3.2 Qualitative Designs

We used the Mixed Methods Appraisal Tool [50], with quality determined by criteria fulfillment percentages (100%: all criteria met, 80%: most criteria met, 60%: some criteria met, 40%: few criteria met, 20%: minimal criteria met).

### 2.4 Data Analysis

The review's analysis employed separate random effect meta‐analyses for between‐group comparisons (YN vs. active comparator and YN vs. no comparator) and within‐group comparisons (pre‐ vs. post‐YN). These analyses were carried out using Comprehensive Meta‐Analysis software (Version 4.0) [51]. Data were organized and analyzed separately for stress, anxiety, and depression outcomes. For studies where data were only available in graphical form, the authors initially attempted to contact the relevant researchers for descriptive statistics. If no response was received, data were extracted using the WebPlotDigitizer application [52]. In studies where the standard deviations of mean changes were not reported, pre‐ and post‐intervention standard deviations were used to calculate the changes in standard deviations, using Meta‐Analysis accelerator, an open‐access validated tool [53]. Furthermore, subgroup analyses were conducted based on study design (randomized vs. nonrandomized) and health status (e.g., healthy, cancer, menstrual disorders). The meta‐analysis outcomes included weighted and adjusted effect sizes (Hedges' *g*), 95% confidence intervals, and significance levels. Effect sizes were interpreted as follows: small (0.16 to <0.38), medium (≥0.38 to <0.76), and large (≥0.76). Forest plots were used to visually represent the results. Heterogeneity was assessed using *I* ^2^ statistics, with thresholds indicating negligible (0%–25%), moderate (25%–75%), or substantial (>75%) heterogeneity. Additionally, “leave‐one‐out” sensitivity analyses were conducted to evaluate the robustness of the findings by systematically removing each study to assess its impact on the overall results. Publication bias for between‐group analyses (with and without an active comparator) was examined using Duval and Tweedie's trim‐and‐fill procedure. An alpha level of 5% was set for statistical significance.

## 3 Results

**FIGURE 1:**
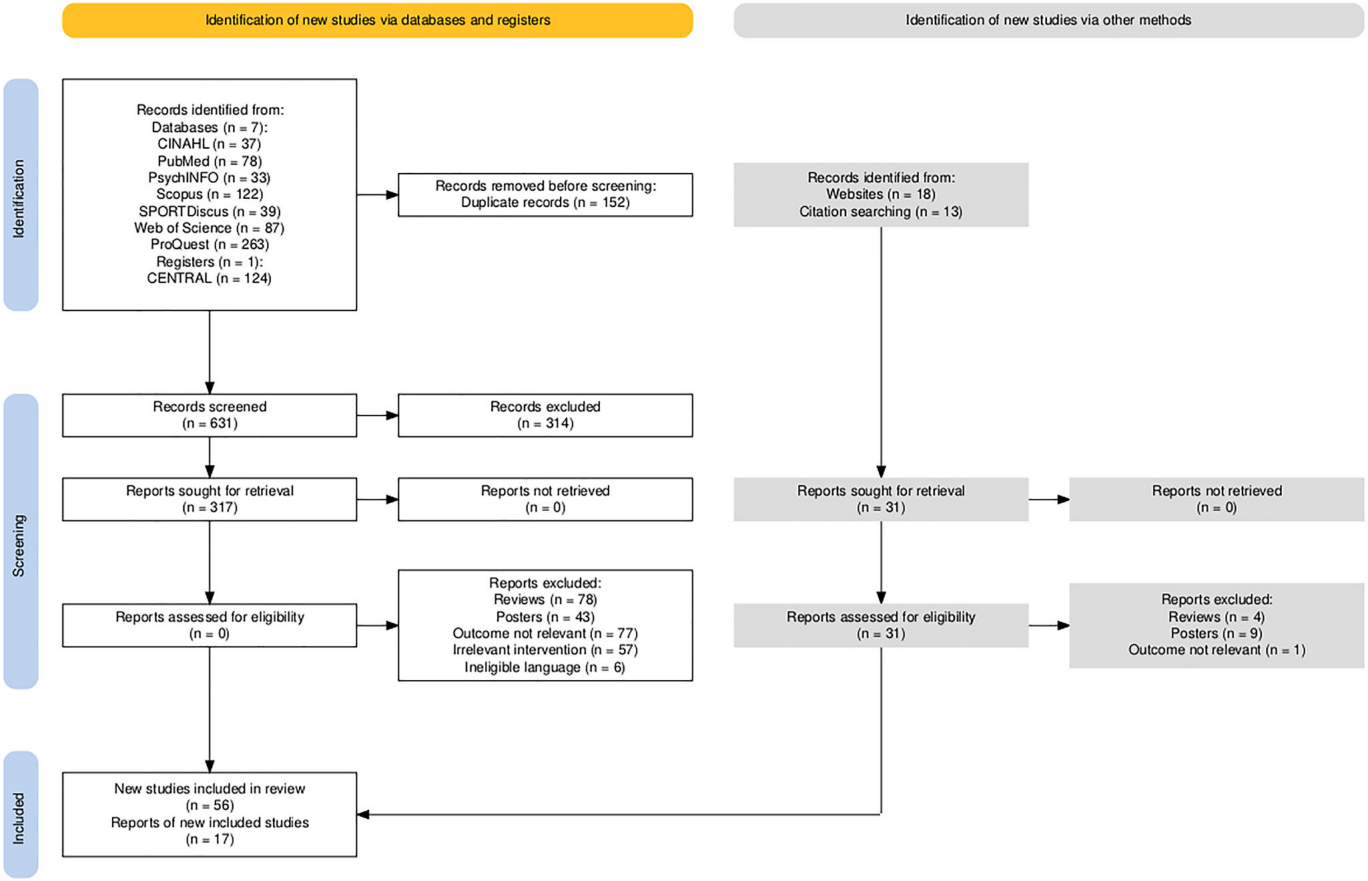
PRISMA flowchart developed using the online tool by Haddaway et al. [54].

The search across seven databases and one trial database initially identified 783 articles. After applying the inclusion criteria, this number was reduced to 56. Additionally, an examination of citations from the assessed full texts revealed 13 more relevant articles. A gray literature search across the first 10 pages of the National Digital Library of India and Google Scholar identified 18 additional relevant articles, which were further assessed. Following an additional screening, 17 of these articles were included, bringing the final total to 73 articles. Details of all the individual extracted studies have been provided in Table S3. The complete selection process is illustrated in the PRISMA flowchart in Figure 1.

### 3.1 Study Design

Of the 73 included studies, the largest proportion (36%) used a quasi‐experimental design with a pre‐post approach, followed by 30% which were randomized controlled trials (RCTs), and 23% non‐RCTs. The remaining studies comprised 7% case series, 3% case studies, and 1% qualitative case series. Detailed information on study designs for individual studies is provided in Table S3.

### 3.2 Country of Study

Geographically, the majority of the studies were conducted in India, representing 64% of all included studies. This was followed by 22% conducted in the USA and 3% studies in Germany. The remaining studies were distributed across Italy, South Korea, Australia, Brazil, Iceland, and Hungary, with each country contributing one study (1% each). The complete geographical distribution is detailed in Table S3.

### 3.3 Study Quality

#### 3.3.1 Randomized Designs

**FIGURE 2:**
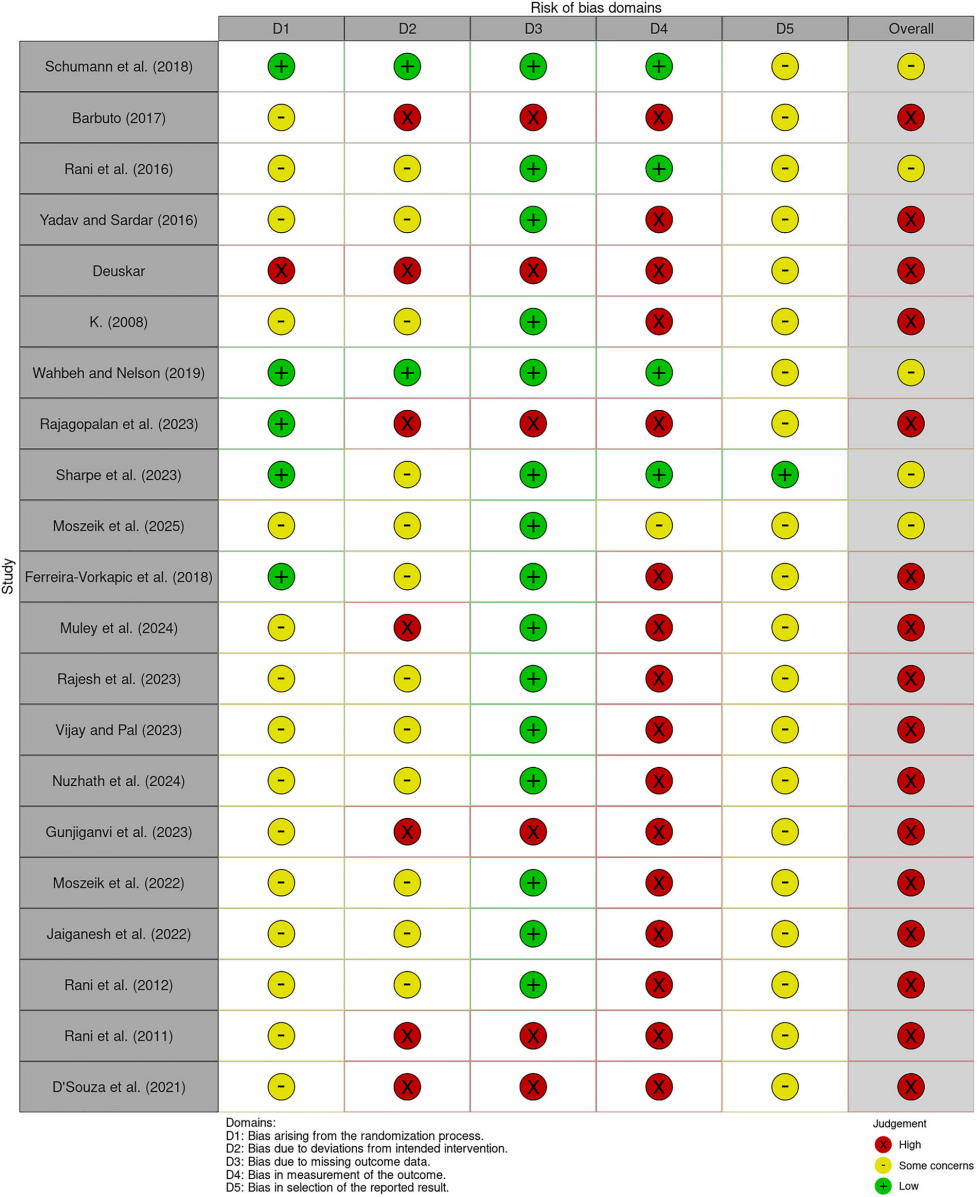
Risk of bias assessment for randomized controlled trials by Cochrane Risk of Bias 2 tool using robvis tool [48] (D1: Randomization process; D2: Deviations from intended interventions; D3: Missing outcome data; D4: Measurement of the outcome; D5: Selection of the reported result).

The quality of 22 RCTs was assessed using the Cochrane Risk of Bias 2.0 tool. Figure 2 and Figure S1 summarize the risk of bias across these trials. The assessment revealed a predominantly high overall risk of bias, with 17 studies classified as high risk [36, 38, 41, 55−67], and five studies were rated as having some concerns [30, 40, 68−70]. Among the individual bias domains, the highest risk was observed in the measurement of outcomes, where 17 studies (77.2%) were judged to have a high risk of bias. This was primarily due to a lack of assessor blinding, as 50% of the RCTs either failed to blind outcome assessors or did not report blinding procedures, thereby potentially compromising the objectivity of outcome evaluation.

### 3.4 Nonrandomized Designs

The methodological quality of 50 studies was evaluated using the Downs and Black checklist (Table 1). The studies had an average score of 11.3 ± 2.5 out of a possible 28 points, indicating an overall “poor” quality. Individually, nine studies were rated as “fair” quality [39, 71−78], with scores ranging from 14 to 16, while the remaining 41 studies were classified as “poor” [42, 79−118], scoring between 6 and 13.

**TABLE 1:** Downs and Black checklist scoring.

Studies	Reporting	External validity	Study bias	Confounding and selection bias	Power	Overall	Quality
Panigrahi et al. [118]	5	0	3	0	0	8	Poor
Shivaji and Dnyeshwar [78]	6	1	4	3	0	14	Fair
Magnúsdóttir et al. [42]	8	0	2	3	0	13	Poor
Tanna and Khatri [76]	10	1	3	1	1	16	Fair
Ravi et al. [110]	6	1	2	1	0	10	Poor
Kumar et al. [99]	8	0	4	1	0	13	Poor
Kamble et al. [97]	7	0	4	0	0	11	Poor
di Fronso et al. [71]	8	0	4	3	0	15	Fair
Navarange et al. [106]	7	1	2	1	0	11	Poor
Neha and Kumar [107]	7	0	3	2	0	12	Poor
Barik [81]	6	0	1	1	0	8	Poor
Graham [91]	5	0	0	1	0	6	Poor
Gupta et al. [92]	5	0	2	2	0	9	Poor
Singh et al. [112]	7	0	3	1	0	11	Poor
Kalita and Choudhury [96]	6	1	3	1	0	11	Poor
Kannan and Kumar [98]	5	0	1	1	0	7	Poor
Dwivedi [72]	7	1	3	3	0	14	Fair
Sharpe et al. [111]	7	0	4	0	0	11	Poor
Sullivan et al. [114]	4	1	1	0	0	6	Poor
Kalita [95]	5	0	3	1	0	9	Poor
Kaur and Sharma [73]	8	1	3	2	0	14	Fair
Joshi [94]	7	0	3	2	0	12	Poor
De Jesus et al. [117]	6	1	4	0	0	11	Poor
Dol [39]	9	0	4	2	1	16	Fair
Livingston and Collette‐Merrill [103]	7	1	5	0	0	13	Poor
Vaishnav et al. [115]	8	0	3	1	0	12	Poor
Tripathi [77]	7	1	5	2	0	15	Fair
Lakshmipathy and Easvaradoss [102]	5	0	4	1	0	10	Poor
Varma and Khan [116]	6	3	3	1	0	13	Poor
Anderson et al. [79]	5	1	0	0	0	6	Poor
Foulkrod et al. [89]	9	0	3	1	0	13	Poor
Ferguson [88]	8	0	4	0	0	12	Poor
Genovese and Fondran [90]	7	0	3	1	0	11	Poor
Manik and Gartia [74]	8	2	3	2	0	15	Fair
Singh and Adhikari [113]	4	0	3	1	0	8	Poor
Chaudhary and Pal [85]	5	0	2	1	0	8	Poor
Pence et al. [75]	9	1	4	0	0	14	Fair
Rani et al. [109]	7	1	3	1	0	12	Poor
Chowdhary [86]	5	0	2	1	0	8	Poor
Eastman‐Mueller et al. [87]	7	0	4	0	0	11	Poor
Jensen et al. [93]	8	0	4	1	0	13	Poor
Lukács et al. [104]	8	0	3	2	0	13	Poor
Bhogaonker [82]	8	1	3	1	0	13	Poor
Anuja [80]	7	0	3	1	0	11	Poor
Birdsall et al. [84]	7	0	3	0	0	10	Poor
Pritchard et al. [108]	7	1	3	1	0	12	Poor
Kumar [101]	6	0	3	1	0	10	Poor
Kumar [100]	7	0	3	2	0	12	Poor
Mishra and Sinha [105]	7	0	3	1	0	11	Poor
Bhushan and Sinha [83]	7	0	3	1	0	11	Poor

*Note*: Scoring ranges: Reporting (0−11), External validity (0−3), Study bias (0−7), Confounding and selection bias (0−6), Power (1). Cutoffs: Excellent (24−28), Good (19−23), Fair (14−18), or Poor (<14).

### 3.5 Qualitative Design

The qualitative case series by Stankovic [119] was assessed using the MMAT. The study met six out of seven criteria, corresponding to 80% of the MMAT quality standards. It demonstrated a clear research question, an appropriate qualitative approach, adequate data collection methods, well‐derived findings, and sufficient substantiation of results. However, coherence between qualitative data sources, collection, analysis, and interpretation remained unclear. Based on these criteria, the study was rated four stars, indicating high methodological quality with a minor area of uncertainty.

### 3.6 Population

Across the 73 studies included in this review, data were reported for a total of 5201 individuals. Of these, 19 studies, encompassing 1215 participants, did not specify the sex distribution of their sample [38, 41, 57, 60, 63, 66, 67, 79, 81, 82, 84−86, 91, 97, 108, 111, 114, 116]. Among the remaining 54 studies, sex distribution was reported for 2760 females, 1225 males, and one diverse individual. Regarding age data, 12 studies did not provide age information for their sample [72, 78, 81, 84, 85, 91, 95, 96, 108, 111, 113, 114]. Age was reported as a range in 33 studies [37, 41, 55−60, 63, 66−68, 73, 77, 79, 80, 82, 83, 86, 87, 90, 94, 97, 99−101, 105, 107, 109, 112, 115, 116, 119]. The remaining 28 studies provided specific age characteristics, allowing for the calculation of a weighted average age of 33.4 years (range: 11–82 years).

Furthermore, across the 73 studies, a total of 3055 (1580 F, 741 M, 1 diverse) individuals received the YN intervention. The discrepancy in sex distribution reporting arose from 19 studies that did not specify the sex breakdown of their sample. The weighted average of this group was 36.9 years. Additionally, 27 studies included a group that received no intervention, meaning there was no comparator to the YN group [30, 37−39, 41, 55−63, 66, 68, 72, 73, 77, 86, 94−96, 102, 104, 107, 116]. This group comprised 1426 individuals (807 F, 381 M). The discrepancy in sex distribution was due to eight studies that did not report sex‐specific data [38, 41, 57, 60, 63, 66, 86, 116]. The weighted age average for this sample was 31.7 years. Likewise, studies included a comparator group, with variations in the type of comparison used. Among these, five studies reported the use of yoga asanas (i.e., postures) [57, 63, 67, 100, 101], while three studies used medications [64, 65, 69], and two used *prāṇāyāma* (i.e., yoga involving breath regulation) [74, 86]. Additionally, one study each used mindfulness‐based meditation [38], housing services for the homeless [82], a vacation retreat with music listening [40], sleep education [42], cyclic meditation [58], nutritional counselling [70], relaxation sleep music [36], and one used Santulan Om meditation music [68]. Across these 18 studies, data were reported for a total of 720 individuals (373 females and 103 males). The discrepancy in sex distribution resulted from six studies that did not report sex‐specific data [38, 57, 63, 67, 82, 86]. The weighted average age of this group was 34 years.

### 3.7 Health Status

A description of the health status of the population included in the review has been provided in Table 2.

**TABLE 2:** Health status of the population.

Health status	Additional subgroup information	Number of studies; references	Sample size (female, male)	Age (weighted average)	Descriptives not reported; references
Healthy	Adolescents	7; [42, 55, 57, 66, 91, 94, 115]	322 (101 F, 115 M)	17.2	6; [55, 57, 66, 91, 94, 115]
	Adults	38; [36, 38, 39, 41, 56, 58−60, 63, 67, 68, 71−73, 77−81, 83, 84, 86−88, 90, 92, 97, 99−104, 107, 109, 112, 113, 118]	3584 (1878 F, 933 M)	31	31; [36, 38, 41, 56, 58−60, 63, 67, 68, 72, 73, 77−81, 83, 84, 86, 87, 90, 92, 97, 99−101, 107, 109, 112, 113]
Menstrual disorders	—	3; [64, 65, 69]	347 (347 F)	27.5	—
Cancer	Cervical cancer	2; [37, 61]	118 (118 F)	52.2	1; [37]
	—	1; [108]	7 (?)	nr	1; [108]
CKD (undergoing hemodialysis)	—	2; [95, 96]	60 (22 F, 38 M)	nr	2; [95, 96]
Hypertension	—	3; [62, 74, 76]	234 (148 F, 86 M)	44.1	—
	Idiopathic intracranial hypertension	1; [110]	1 (1 F)	16	—
Depression	—	2; [40, 89]	33 (26 F, 7 M)	62.1	—
PTSD	—	2; [114, 119]	23 (16 M)	nr	2; [114, 119]
GAD	—	2; [98, 106]	4 (2 F, 2 M)	30	—
Insomnia	—	2; [30, 111]	94 (16 F, 4 M)	31.5	2; [30, 111]
Gastrointestinal disorders	—	1; [105]	22 (12 F, 10 M)	nr	1; [105]
	IBS	1; [70]	59 (52 F, 7 M)	54.9	—
Spondylitis and back ache	—	1; [85]	20 (?)	nr	1; [85]
Angina pectoris	—	1; [116]	30 (?)	nr	1; [116]
With/risk of CVD	—	1; [117]	16 (16 F)	64	—
Behavioral dysfunction	—	1; [93]	7 (7 M)	12.6	—
Sexual trauma	—	1; [75]	15 (15 F)	56	—
Homeless	—	1; [82]	196 (?)	nr	1; [82]
Multiple sclerosis	—	1; [108]	9 (?)	nr	1; [108]

*Note*: “nr” indicates that the value was not reported. Abbreviations: CKD, chronic kidney disease; CVD, cardiovascular disease; GAD, generalized anxiety disorder; IBS, irritable bowel syndrome; PTSD, post‐traumatic stress disorder.

## 4 YN Intervention Details

### 4.1 Intervention Structure and Instructor

Among the 73 included studies, 38 studies reported details regarding session structure, whereas 35 did not. Similarly, 53 studies reported the presence of an instructor during sessions, while 20 did not (Table 3).

**TABLE 3:** Details of studies reporting on session structure and instructor presence.

	Reported	Not reported
Session structure	38; [30, 36, 37, 39−41, 55, 56, 58, 59, 63, 64, 66, 68, 71, 73, 75, 76, 80−84, 87, 88, 91, 93, 94, 97, 100, 101, 103, 105, 108−110, 112, 119]	35; [38, 42, 57, 60−62, 65, 67, 69, 70, 72, 74, 77−79, 85, 86, 89, 90, 92, 95, 96, 98, 99, 102, 104, 106, 107, 111, 113−118]
Instructor presence	53; [30, 36−42, 55, 58−66, 68−71, 73, 75−77, 79, 81−83, 87−91, 93, 94, 97, 99−105, 109, 110, 112, 113, 115, 117−119]	20; [56, 57, 67, 72, 74, 78, 80, 84−86, 92, 95, 96, 98, 106−108, 111, 114, 116]

### 4.2 Type of YN

Thirty‐eight studies indicated following a YN which adheres to the guidelines mentioned by Swami Satyananda Saraswati at Bihar School of Yoga [30, 36−39, 55−60, 62−65, 67−69, 72, 73, 76−78, 80, 83, 86, 88, 89, 92, 93, 100−102, 104, 105, 109, 115, 116]. Eleven studies mentioned using iRest (an adaptation form of YN) [40, 41, 75, 82, 84, 87, 103, 108, 112, 114, 119], one study used a version of YN suggested by Anandmurti Gurumaa ji [91]. Twenty‐three studies had not reported if they followed any specific type of YN [42, 61, 66, 70, 71, 74, 79, 81, 85, 90, 94−99, 106, 107, 110, 111, 113, 117, 118].

### 4.3 Delivery Setting

Forty‐four studies delivered YN sessions in person [30, 37−39, 42, 55−58, 60−67, 70, 71, 73, 75−80, 82, 83, 86, 89, 93, 94, 97, 100−103, 105, 109, 113−117], with participants physically attending the sessions. Additionally, six studies conducted sessions exclusively at home [36, 59, 68, 92, 111, 112], while 13 studies implemented both in‐person and at‐home sessions [40, 41, 69, 84, 87, 88, 91, 98, 104, 108, 110, 118, 119]. Ten studies did not report the setting in which YN was delivered [72, 74, 81, 86, 90, 95, 96, 99, 106, 107].

### 4.4 Training Dosage

Eight of the included studies either did not report or incompletely reported the duration of training their participants received [72, 81, 91, 98, 111, 114, 116, 118]. For the remaining studies, training dosage was standardized by calculating the total training time in minutes, determined by multiplying the duration of each session by the number of sessions delivered. Across all included studies, the median training duration was 750 min, with a range of 30−4550 min.

### 4.5 Type of Assessment

Stress was assessed in 45 studies. The impact of YN on stress was evaluated using the Perceived Stress Scale in 14 studies [40, 41, 71, 76, 81, 82, 84, 87, 88, 102, 108, 110, 117, 118], the Depression, Anxiety, and Stress Scale in six studies [62, 90, 92, 95, 96, 112], and nonspecified stress scales in four studies [66, 72, 79, 85]. Additionally, stress was measured using the Post‐Traumatic Stress Disorder Checklist [75, 114], and the Eight State Questionnaire in two studies each [100, 101]. One study, in addition to using the Perceived stress scale, had used the Cohen's Perceived Stress Scale [70].

Additionally, one study each used the Visual Analogue Scale for Perceived Stress [115], Stress Relaxation Score [60], Stress Questionnaire [37], Stress and Social Adjustment Scale [86], Department of Defense Pain‐Stress Subset [103], Everly and Girnando Questionnaire for Stress [57], Hassles and Uplift Scale [116], Life Stress Intensity by visual analogue scale [39], Lipp's Stress Syndrome [38], Modified Adolescent Stress Questionnaire [55], Modified Stress Assessment Scale [109], Occupational Stress Index [113], Trier Inventory for Chronic Stress 12‐item [68], and Screening Scale for Chronic Stress [59]. Additionally, one study performed a qualitative self‐rating data on post‐traumatic stress disorder symptoms [119], and another used a self‐made questionnaire to assess stress [91].

Anxiety was assessed in 43 studies, with the impact of YN being most frequently evaluated using the Depression Anxiety and Stress Scale, which was employed in seven studies [62, 78, 90, 92, 95, 96, 112]. This State‐Trait Anxiety Inventory was used in six studies [30, 68, 93, 105, 111, 116], followed by the Hamilton Anxiety Rating Scale [65, 70, 73, 74, 106], and the Generalized Anxiety Disorder‐7 scale [36, 42, 66, 89, 117], each of which was used in five studies. The Beck Anxiety Inventory was used in four studies [38, 63, 67, 98], and Sinha's Comprehensive Anxiety Rating Scale in three studies [80, 99, 107]. The Eight State Questionnaire [100, 101] and the Psychological General Well‐Being Index were each used in two studies [64, 69]. Additionally, one study each assessed anxiety using the Anxiety scale [77], Brief symptom inventory [75], Competition State Anxiety Inventory for assessing somatic and cognitive anxiety [58], Hospital Anxiety and Depression scale [61], Kellner symptom questionnaire [82], Smith stress symptoms inventory [56], and the State trait and free‐floating anxiety scale [94]. One study used both the General Anxiety Disorder‐7 scale and the Beck Anxiety Inventory [97], while another used both the State‐Trait Anxiety Inventory and the Anxiety Scale of Derogatis's Symptomatic Checklist [83].

Depression was assessed in 27 studies, with the most common assessment tool being the Depression, Anxiety, and Stress Scale [62, 90, 92, 95, 96, 112], used in six studies. This was followed by the Beck Depression Inventory, which was employed in five studies [38, 42, 87, 94, 104], the Patient Health Questionnaire‐9 in four studies [36, 89, 114, 117], and the Hamilton Depression Rating Scale in three studies [65, 70, 73]. Additionally, one study each utilized the Kellner Symptom Questionnaire [82], Brief Symptom Inventory [75], Center for Epidemiologic Studies Depression Scale‐5 [40], Depression Scale of the Symptomatic Checklist [105], Hospital Anxiety and Depression Scale [61], Profile of Mood States [84], State Trait Depression Inventory [68], and the Psychological General Well‐Being Index [69]. One study reported changes in depression but did not specify the assessment tool used [98].

## 5 Outcomes

### 5.1 Between‐Group

#### 5.1.1 YN Versus Active Comparator

1. Stress: Among the nine studies comparing YN to an active comparator, three reported significant improvements in stress outcomes [62, 100, 101], while six found no significant difference [38, 40, 57, 68, 70, 86].
2. Anxiety: Among the 15 studies comparing YN to an active comparator, 11 reported significant improvements in anxiety outcomes [36, 61−65, 67, 69, 70, 100, 101], while four found no significant difference [38, 42, 58, 68].
3. Depression: Among the 11 studies comparing YN to an active comparator, seven reported significant improvements in depression outcomes [36, 61, 62, 64, 65, 68, 69], whereas four found no significant difference [38, 40, 42, 70].

#### 5.1.2 YN Versus No Comparator

1. Stress: Among the 13 studies comparing YN to no intervention, 12 reported significant improvements in stress outcomes [37, 39, 55, 57, 59, 60, 68, 72, 86, 102, 116], while one found no significant difference [41].
2. Anxiety: Of the 10 studies comparing YN to no intervention, eight reported significant improvements in stress outcomes [38, 56, 58, 68, 73, 77, 107, 116], whereas two found no significant difference [30, 94].
3. Depression: Of the five studies comparing YN to no intervention, three reported significant improvements in depression outcomes [68, 73, 104], while two found no significant difference [38, 94].

#### 5.1.3 Within‐Group

1. Stress: Of the 40 studies evaluating the within‐group effects of YN on stress, 30 reported significant improvements in stress outcomes [37, 39, 41, 55, 62, 66, 68, 70,72, 75, 76, 81, 82, 84, 85, 87, 88, 90, 92, 95, 96, 102, 108, 109, 112, 113, 115, 116, 118], while four found no significant differences [40, 59, 103, 117]. Additionally, three studies noted a reduction in stress but did not include statistical analysis [79, 110, 114]. One study reported a decrease in the number of adolescents experiencing high stress levels [91], also without a statistical analysis. A qualitative study found reduced stress outcomes [119], and a case series observed stress reduction in one case but not the other [71].
2. Anxiety: Of the 36 studies evaluating the within‐group effects of YN on anxiety, 29 reported significant improvements in anxiety outcomes [36, 38, 58, 61−63, 65−70, 73, 74, 78, 80, 82, 83, 90, 92, 94−97, 99, 105, 111, 112, 116], while five found no significant differences [30, 75, 89, 93, 117]. Additionally, two case studies [98, 106] reported reductions in anxiety outcomes, though neither included statistical analysis.
3. Depression: Of the 26 studies evaluating the within‐group effects of YN on depression, 20 reported significant improvements in depression outcomes [36, 40, 61, 62, 65, 68−70, 73, 75, 82, 87, 89, 90, 92, 94−96, 104, 112], while four found no significant differences [38, 84, 105, 117]. Additionally, one study indicated a trend toward as a result of YN [114], and one case study reported a reduction in depression, though neither of these studies included statistical analysis [98].

### 5.2 Publication Bias

**FIGURE 3:**
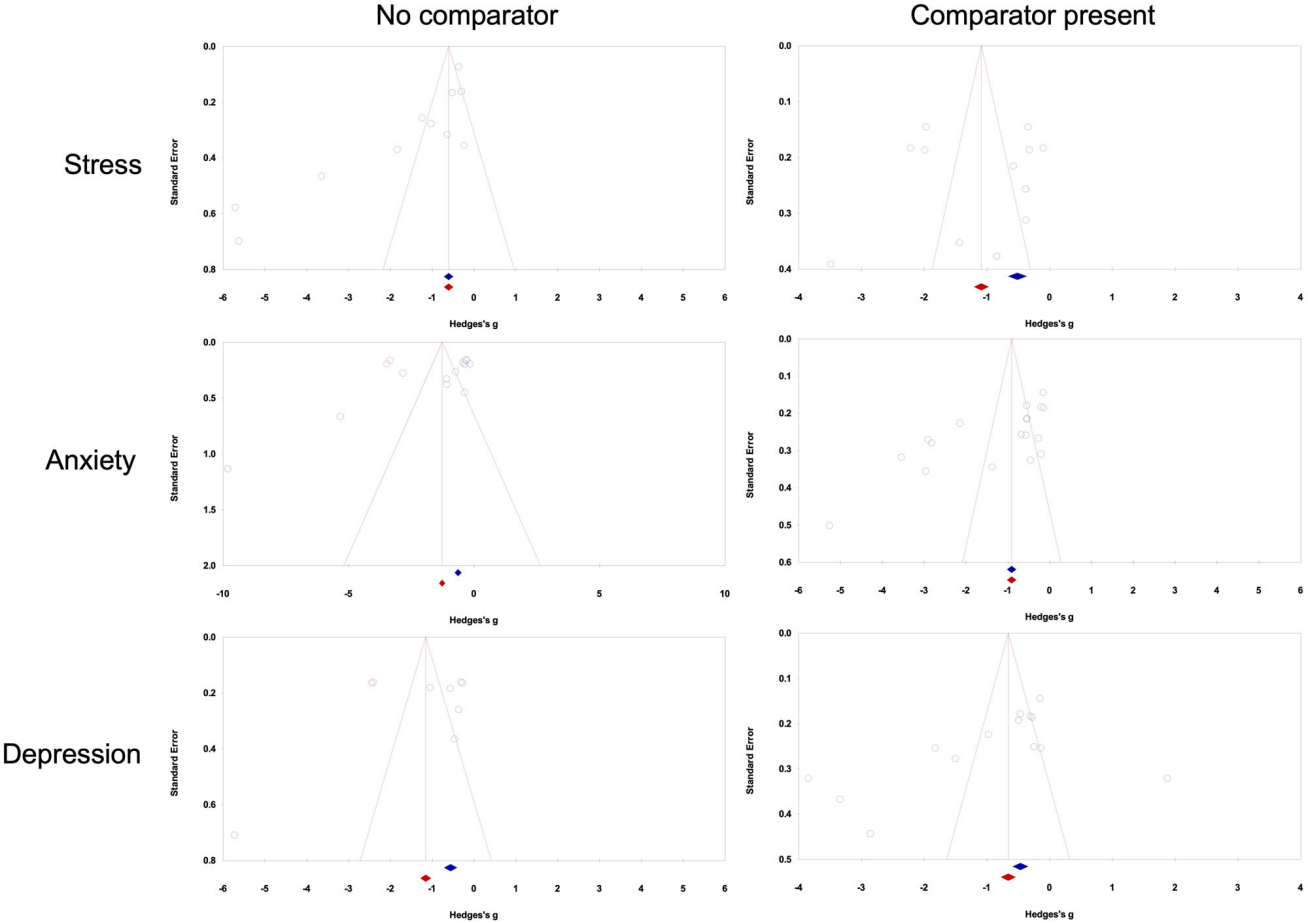
Publication bias for between‐group analyses comparing the efficacy of Yoga Nidra with a passive comparator (Left) and active comparator (right) on stress, anxiety, and depression.

The assessment of publication bias was conducted while using Duval and Tweedie's trim‐and‐fill method using the random effect model for both between‐group analyses, that is, YN versus no comparator and versus a comparator for stress, anxiety, and depression. The outcome of individual assessments has been illustrated in Figure 3 and has been provided in the following sections.

#### 5.2.1 Stress

##### 5.2.1.1 No Comparator

The analysis found no missing studies on either side of the mean effect. The point estimate and 95% confidence interval (CI) were −1.70 (−2.32 to −1.08). Applying the trim‐and‐fill method did not alter these values.

##### 5.2.1.2 Comparator Present

The analysis identified three missing studies on the left side of the mean. The point estimate and 95% CI were −0.80 (−1.27 to −0.33), after applying the trim‐and‐fill method, the imputed point estimate adjusted to −1.14 (−1.69 to −0.60).

#### 5.2.2 Anxiety

##### 5.2.2.1 No Comparator

The analysis identified two missing studies on the left side. The point estimate and 95% CI were −1.43 (−2.05 to −0.81), after applying the trim‐and‐fill method, the imputed point estimate adjusted to −1.87 (−2.68 to −1.06).

##### 5.2.2.2 Comparator Present

The analysis found no missing studies on either side of the mean effect. The point estimate and 95% confidence interval were −1.35 (−1.89 to −0.82). Applying the trim‐and‐fill method did not alter these values.

#### 5.2.3 Depression

##### 5.2.3.1 No Comparator

The analysis identified two missing studies on the left side. The point estimate and 95% CI were −0.92 (−1.47 to −0.37), after applying the trim‐and‐fill method, the imputed point estimate adjusted to −1.37 (−2.10 to −0.64).

##### 5.2.3.2 Comparator Present

The analysis identified two missing studies on the left side. The point estimate and 95% CI were −0.69 (−1.19 to −0.19), after applying the trim‐and‐fill method, the imputed point estimate adjusted to −1.00 (−1.58 to −0.42).

### 5.3 Meta‐Analysis Outcomes

**FIGURE 4:**
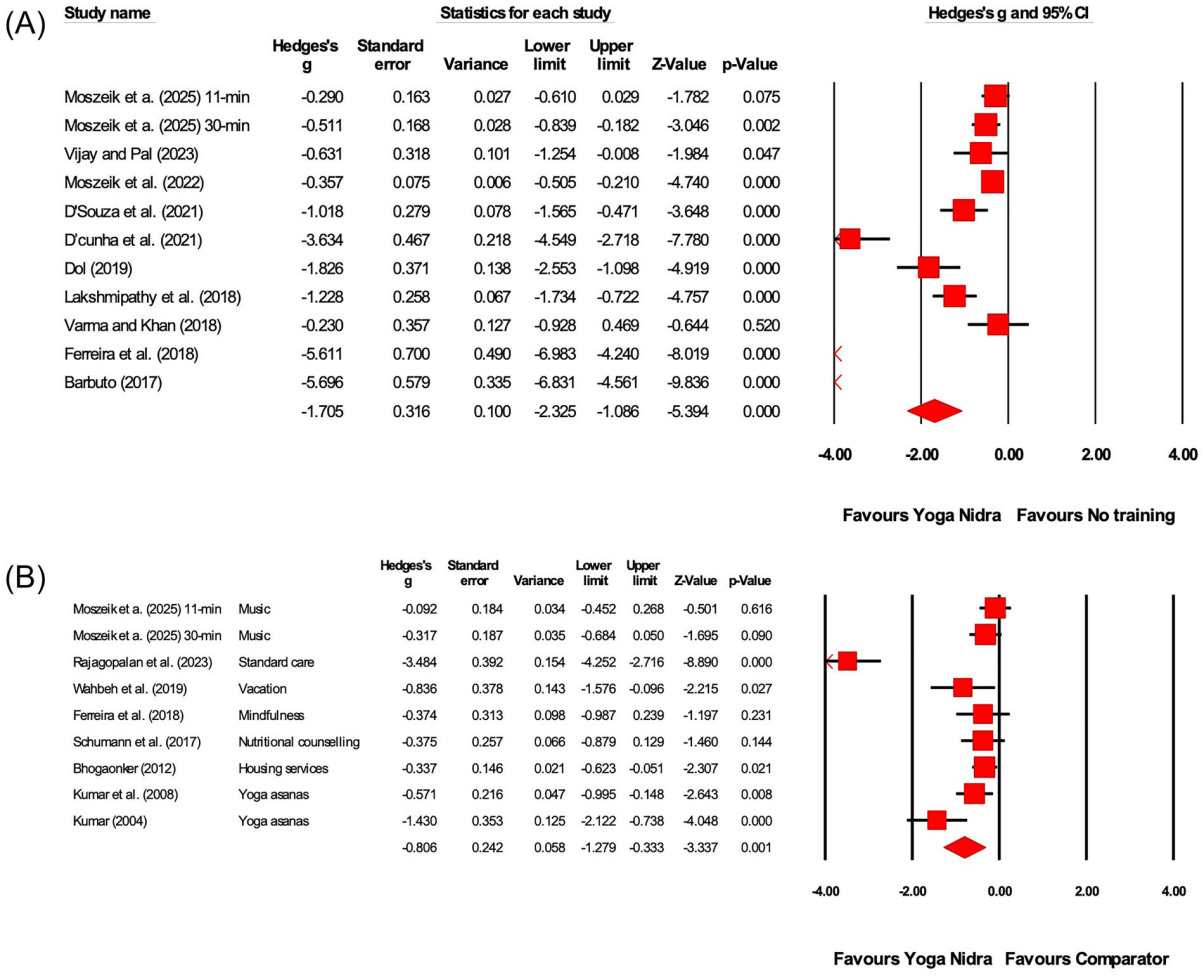
Between‐group meta‐analysis demonstrating the effects of Yoga Nidra compared to (A) no training comparator, (B) comparator on stress outcomes. A list of comparator interventions used by the studies has been provided in (B). Each horizontal line represents the 95% confidence interval (CI) for the effect size (Hedges' *g*) of individual studies. Negative values of Hedges' *g* indicate a favorable effect of Yoga Nidra on reducing stress. The size of the red squares reflects the weight of each study in the meta‐analysis, and the diamond represents the overall effect size and confidence interval.

**FIGURE 5:**
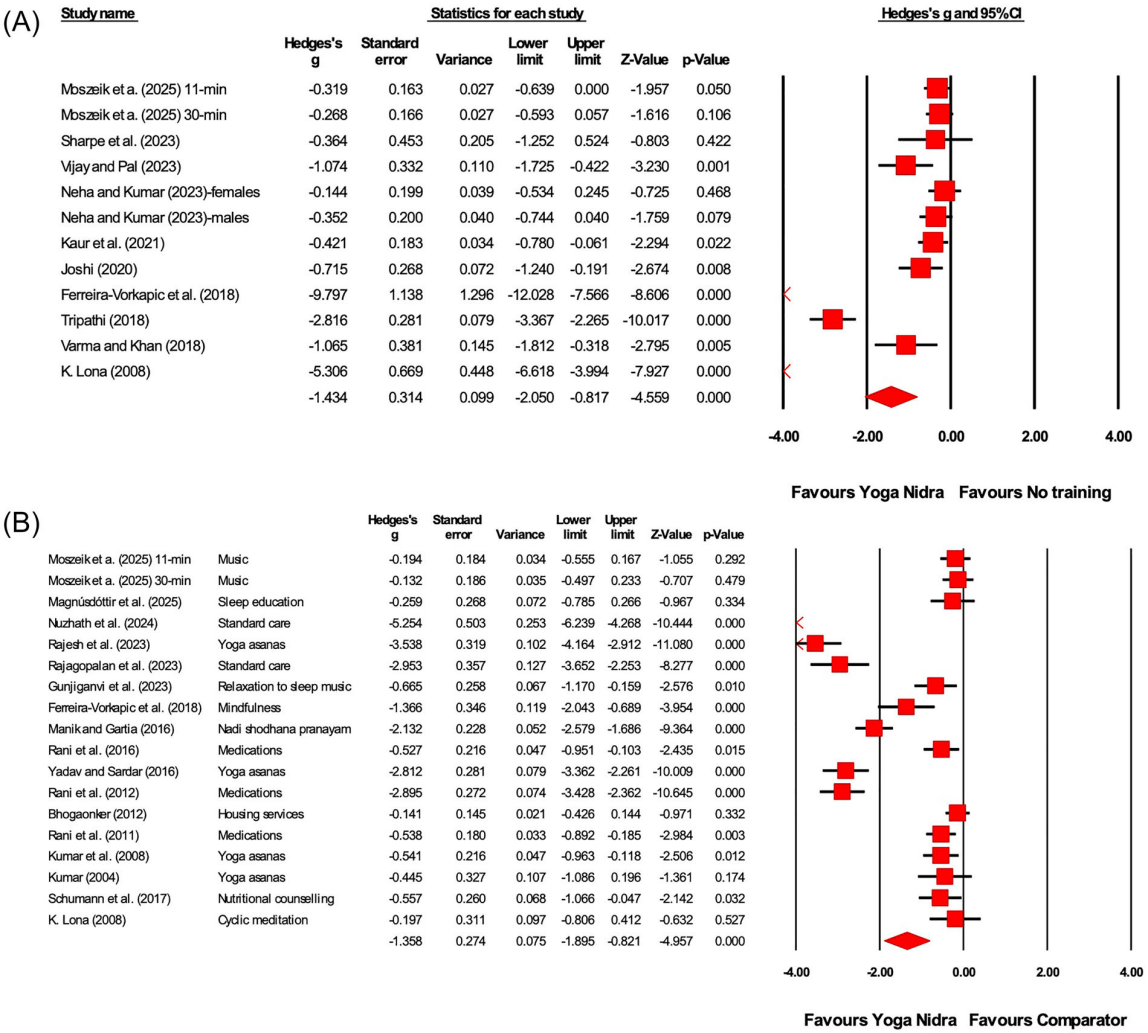
Between‐group meta‐analysis demonstrating the effects of Yoga Nidra compared to (A) no training comparator, (B) comparator on anxiety outcomes. A list of comparator interventions used by the studies has been provided in (B). Each horizontal line represents the 95% confidence interval (CI) for the effect size (Hedges' *g*) of individual studies. Negative values of Hedges' *g* indicate a favorable effect of Yoga Nidra on reducing anxiety. The size of the red squares reflects the weight of each study in the meta‐analysis, and the diamond represents the overall effect size and confidence interval.

**FIGURE 6:**
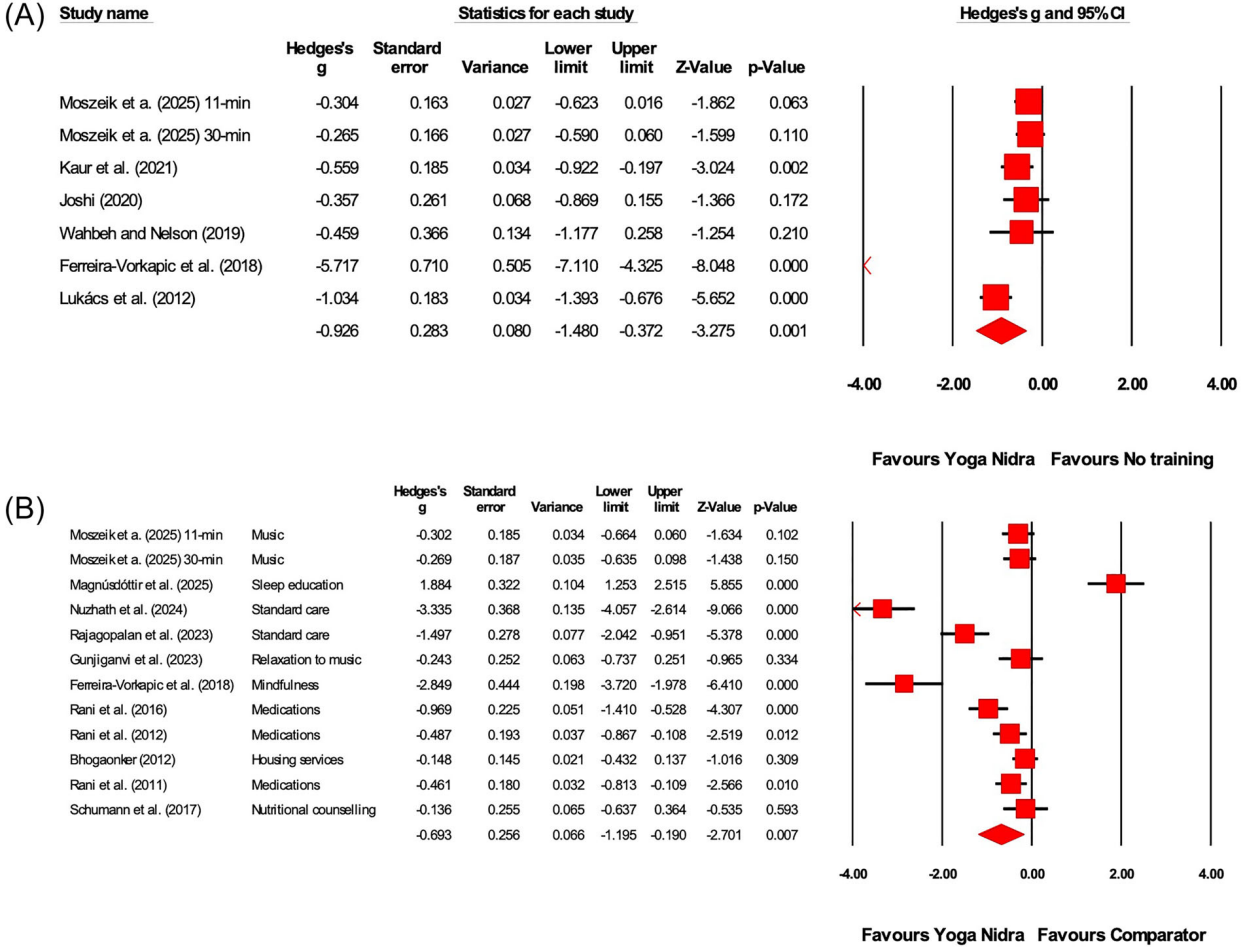
Between‐group meta‐analysis demonstrating the effects of Yoga Nidra compared to (A) no training comparator, (B) comparator on depression outcomes. A list of comparator interventions used by the studies has been provided in (B). Each horizontal line represents the 95% confidence interval (CI) for the effect size (Hedges' *g*) of individual studies. Negative values of Hedges' *g* indicate a favorable effect of Yoga Nidra on reducing depression. The size of the red squares reflects the weight of each study in the meta‐analysis, and the diamond represents the overall effect size and confidence interval.

Tables 4 and 5 present an overview of the meta‐analysis outcomes, highlighting the between‐ and within‐group effects of YN (Figures 4, 5−6). The between‐group analyses separately evaluate studies that compared YN with and without a comparator. Additionally, the results include subgroup analyses based on study design randomization and the health status of the included populations.

**TABLE 4:** Between‐group analysis results.

S. no	Analysis	Number of studies	Hedges' *g* (95% C.I., *p*‐value), *I* ^2^ statistics	Figure
Between group: stress (no comparator)				
1.	Overall	10; [37, 38, 39, 41, 55, 59, 68, 102, 116, 120]	−1.70 (−2.32 to −1.08, *p*<0.001), *I* ^2^: 71.3%	3A
Randomization				
2.	RCT	7; [37, 38, 41, 55, 59, 68, 120]	−1.97 (−2.74 to −1.19, *p*<0.001), *I* ^2^: 75.6%	S2
3.	NRCT	3; [39, 102, 116]	−1.09 (−1.92 to −0.26, *p* = 0.009), *I* ^2^: 14.4%	S3
Population				
4.	Healthy (adults)	6; [38, 39, 41, 59, 68, 102]	−1.92 (−2.71 to −1.14, *p*<0.001) *I* ^2^: 78%	S4
5.	Healthy (adolescents)	2; [55, 120]	−0.84 (−1.26 to 0.43, *p*<0.001), *I* ^2^: 0%	S5
6.	Cancer	1; [37]	—	—
7.	Angina pectoris	1; [116]	—	—

Between group: stress (comparator present)				
8.	Training	8; [38, 40, 62, 68, 70, 82, 100, 101]	−0.80 (−1.27 to −0.33, *p* = 0.001), *I* ^2^: 48%	3B
Randomization				
9.	RCT	5; [38, 40, 62, 68, 70]	−0.86 (−1.62 to −0.11, *p* = 0.024), *I* ^2^: 43.3%	S6
10.	NRCT	3; [82, 100, 101]	−0.69 (−1.21 to −0.17, *p* = 0.009), *I* ^2^: 27.1%	S7
Population				
11.	Healthy (adults)	4; [38, 68, 100, 101]	−0.49 (−0.85 to −0.12, *p* = 0.008), *I* ^2^: 22.7%	S8
12.	IBS	1; [70]	—	—
13.	Depression	1; [40]	—	—
14.	Hypertension	1; [62]	—	—
15.	Homeless	1; [82]	—	—

Between group: Anxiety (no comparator)				
16.	Overall	10;[30, 38, 68, 73, 77, 94, 107, 116, 120, 121]	−1.43 (−2.05 to −0.81, *p*<0.001), *I* ^2^: 77.7%	4A
Randomization				
17.	RCT	5; [30, 38, 68, 120, 121]	−2.32 (−3.53 to −1.11, *p*<0.001), *I* ^2^: 81.5%	S9
18.	NRCT	5; [73, 77, 94, 107, 116]	−0.90 (−1.61 to −0.19, *p* = 0.013), *I* ^2^: 18.1%	S10
Population				
19.	Healthy (adults)	6; [38, 68, 73, 77, 107, 121]	−1.84 (−2.68 to −0.99, *p*<0.001), *I* ^2^: 82.7%	S11
20.	Healthy (adolescents)	2; [94, 120]	−0.85 (−1.26 to −0.44, *p*<0.001), *I* ^2^: 0%	S12
21.	Angina pectoris	1; [116]	—	—
22.	Insomnia	1; [116]	—	—

Between group: Anxiety (comparator present)				
23.	Overall	17; [36, 38, 42, 61−65, 67−70, 74, 82, 100, 101, 121]	−1.35 (−1.89 to −0.82, *p*<0.001), *I* ^2^: 37.4%	4B
Randomization				
24.	RCT	12; [36, 38, 61−65, 67−70, 121]	−1.62 (−2.33 to −0.90, *p*<0.001), *I* ^2^: 33.9%	S13
25.	NRCT	5; [42, 74, 82, 100, 101]	−0.70 (−1.44 to 0.03, *p* = 0.062), *I* ^2^: 0%	S14
Population				
26.	Healthy (adults)	8; [36, 38, 63, 67, 68, 74, 100, 121]	−1.08 (−1.82 to −0.35, *p* = 0.004), *I* ^2^: 17.8%	S15
27.	Menstrual disorders	3; [64, 65, 69]	−1.30 (−2.66 to 0.05, *p* = 0.060), *I* ^2^: 1.6%	S16
28.	Hypertension	2; [62, 74]	−2.49 (−3.29 to −1.69, *p*<0.001), *I* ^2^: 0%	S17
29.	Healthy (adolescents)	1; [42]	—	—
30.	IBS	1; [70]	—	—
31.	Cancer	1; [61]	—	—
32.	Homeless	1; [82]	—	—

Between group: Depression (no comparator)				
33.	Overall	6; [38, 40, 68, 73, 94, 104]	−0.92 (−1.48 to −0.37 *p* = 0.001), *I* ^2^: 77.3%	5A
Randomization				
34.	RCT	3; [38, 40, 68]	−1.38 (−2.46 to −0.29, *p* = 0.013), *I* ^2^: 79.6%	S18
35.	NRCT	3; [73, 94, 104]	−0.67 (−1.06 to −0.28, *p* = 0.001), *I* ^2^: 0%	S19
Population				
36.	Healthy (adults)	4; [38, 68, 73, 104]	−1.18 (−1.91 to −0.45 *p* = 0.001), *I* ^2^: 81.7%	S20
37.	Healthy (adolescents)	1; [94]	—	—
38.	Depression	1; [40]	—	—

Between group: Depression (comparator present)				
39.	Overall	11; [36, 38, 42, 61, 62, 64, 65, 68−70, 82]	−0.69 (−1.19 to −0.19, *p* = 0.007), *I* ^2^: 53.7%	5B
Randomization				
40.	RCT	9; [36, 38, 61, 62, 64, 65, 68−70]	−0.98 (−1.48 to −0.49, *p*<0.001), *I* ^2^: 47.7%	S21
41.	NRCT	2; [42, 82]	0.84 (−1.14 to 2.83, *p* = 0.40), *I* ^2^: 0%	S22
Population				
42.	Menstrual disorder	3; [64, 65, 69]	−0.61 (−0.91 to 0.31, *p*<0.001), *I* ^2^: 5.6%	S23
43.	Healthy (adults)	3; [36, 38, 68]	−0.80 (−1.56 to −0.04, *p* = 0.037), *I* ^2^: 58.7%	S24
44.	Hypertension	1; [62]	—	—
45.	Homeless	1; [82]	—	—
46.	Healthy (adolescents)	1; [42]	—	—
47.	Cancer	1; [61]	—	—
48.	IBS	1; [70]	—	—

Abbreviations: CVD, cardiovascular disease; IBS, irritable bowel syndrome; NRCTs, nonrandomized controlled trials; RCTs, randomized controlled trials.

**TABLE 5:** Within‐group meta‐analysis outcomes.

S. no	Analysis	Number of studies	Hedges' *g* (95% C.I., *p*‐value), *I* ^2^ statistics	Figure
Within group: Stress				
49.	Overall	33; [37, 38, 39, 40, 41, 55, 59, 62, 68, 70−72, 75, 76, 81, 82, 86−88, 92, 102, 103, 108, 109, 112, 114−118, 120, 122, 123]	−1.05 (−1.32 to −0.78, *p*<0.001), *I* ^2^: 72.6%	S25
Randomization				
50.	RCT	11; [37, 38, 40, 41, 55, 59, 62, 68, 70, 109, 120]	−1.79 (−2.46 to −1.13, *p*<0.001), *I* ^2^: 86%	S26
51.	NRCT	22; [39, 71, 72, 75, 76, 81, 82, 86−88, 92, 102, 103, 108, 112, 114−118, 122, 123]	−0.76 (−0.95 to −0.56, *p*<0.001), *I* ^2^: 26.3%	S27
Population				
52.	Healthy (adults)	18; [38, 39, 41, 59, 68, 71, 72, 81, 87, 88, 92, 102, 103, 109, 112, 118, 122, 123]	−0.98 (−1.34 to −0.63, *p*<0.001), *I* ^2^: 84%	S28
53.	Healthy (adolescents)	3; [55, 115, 120]	−1.07 (−1.56 to −0.59, *p*<0.001), *I* ^2^: 7.5%	S29
54.	Hypertension	2; [62, 76]	−2.48 (−5.16 to −0.19, *p* = 0.070), *I* ^2^: 0%	S30
55.	Cancer	2; [37, 108]	−2.20 (−4.39 to −0.02, *p* = 0.048), *I* ^2^: 0%	S31
56.	Homeless	1; [82]	—	—
57.	Angina pectoris	1; [116]	—	—
58.	Depression	1; [40]	—	—
59.	IBS	1; [70]	—	—
60.	PTSD	1; [114]	—	—
61.	With/risk of CVD	1; [117]	—	—
62.	Multiple sclerosis	1; [108]	—	—
63.	Sexual trauma	1; [75]	—	—
64.	Spondylitis and low back pain	1; [86]	—	—

Within group: Anxiety				
65.	Overall	32; [30, 36, 38, 42, 62, 63, 65, 67−70, 73, 75, 77, 78, 80, 82, 83, 89, 92−94, 97, 99, 107, 111, 112, 116, 117, 120, 121, 123]	−1.03 (−1.32 to −0.73, *p*<0.001), *I* ^2^: 64.8%	S32
Randomization				
66.	RCT	12; [30, 36, 38, 62, 63, 65, 67−70, 120, 121]	−1.17 (−1.64 to −0.69, *p*<0.001), *I* ^2^: 82.4%	S33
67.	NRCT	20; [42, 73, 75, 77, 78, 80, 82, 83, 89, 92−94, 97, 99, 107, 111, 112, 116, 117, 123]	−0.94 (−1.32 to −0.56, *p*<0.001), *I* ^2^: 8.6%	S34
Population				
68.	Healthy (adults)	16; [36, 38, 63, 67, 73, 77, 78, 80, 83, 92, 97, 107, 112, 121, 123]	−1.18 (−1.62 to −0.74, *p*<0.001), *I* ^2^: 77.6%	S35
69.	Healthy (adolescents)	3; [42, 94, 120]	−0.76 (−1.09 to −0.43, *p*<0.001), *I* ^2^: 0%	S36
70.	Menstrual disorders	2; [65, 69]	−0.46 (−0.74 to −0.18, *p* = 0.001), *I* ^2^: 0%	S37
71.	Insomnia	2; [30, 111]	−1.18 (−2.99 to 0.62, *p* = 0.198), *I* ^2^: 0%	S38
72.	Hypertension	1; [62]	—	—
73.	With/risk of CVD	1; [117]	—	—
74.	Behavioral dysfunction	1; [93]	—	—
75.	IBS	1; [70]	—	—
76.	Angina pectoris	1; [116]	—	—
77.	Homeless	1; [82]	—	—
78.	Depression	1; [89]	—	—
79.	Pregnant	1; [99]	—	—
80.	Sexual trauma	1; [75]	—	—

Within group: Depression				
81.	Overall	21; [36, 38, 40, 42, 62, 65, 68−70, 73, 75, 82, 87, 89, 92, 94, 104, 112, 117, 122, 123]	−0.71 (−0.99 to −0.42, *p*<0.001), *I* ^2^: 69%	S39
Randomization				
82.	RCT	8; [36, 38, 40, 62, 65, 68−70]	−1.04 (−1.61 to −0.48, *p*<0.001), *I* ^2^: 79.4%	S40
83.	NRCT	13; [42, 73, 75, 82, 87, 89, 92, 94, 104, 112, 117, 122, 123]	−0.53 (−0.84 to −0.22, *p* = 0.001), *I* ^2^: 46.7%	S41
Population				
84.	Healthy (adults)	10; [36, 38, 68, 73, 87, 92, 104, 112, 122, 123]	−0.93 (−1.37 to −0.48, *p*<0.001), *I* ^2^: 79.6%	S42
85.	Healthy (adolescents)	2; [42, 94]	−0.002 (−0.88 to 0.87, *p* = 0.997), *I* ^2^: 0%	S43
86.	Depression	2; [40, 89]	−1.16 (−2.28 to −0.05, *p* = 0.040), *I* ^2^: 0%	S44
87.	Menstrual disorders	2; [65, 69]	−0.49 (−1.11 to 0.12, *p* = 0.116), *I* ^2^: 0%	S45
88.	Hypertension	1; [62]	—	—
89.	Sexual trauma	1; [75]	—	—
90.	With/risk of CVD	1; [117]	—	—
91.	IBS	1; [70]	—	—
92.	Homeless	1; [82]	—	—

Abbreviations: CVD, cardiovascular disease; IBS, irritable bowel syndrome; NRCTs, nonrandomized controlled trials; RCTs, randomized controlled trials.

### 5.4 Sensitivity Analysis

A summarized overview of the sensitivity analysis is presented in Table 6. The leave‐one‐out sensitivity analysis identified specific studies whose removal led to a directional change in the reported *p*‐value. If the original analysis yielded a *p*‐value <0.05, but excluding a particular study resulted in a *p*‐value >0.05, or vice versa, the study's citation was provided along with the adjusted *p*‐value after its removal.

**TABLE 6:** Leave‐one‐out sensitivity analysis.

S. no	Analysis	*p*‐value of meta‐analysis	*I* ^2^ statistics	Removal of studies resulting in *p*‐value change	Change in *p*‐value upon removal	Figure
Between group: Stress (no comparator)						
1.	Overall	<0.001	71.3%	—	No effect	S46
Randomization						
2.	RCT	<0.001	75.6%	No effect	—	S47
3.	NRCT	0.009	14.4%	Dol [39]	0.12	S48
				Lakshmipathy and Easvaradoss [102]	0.19	
4.	Healthy (adults)	<0.001	78%	No effect	—	S49
5.	Healthy (adolescents)	<0.001	0%	No effect	—	S50

Between group: Stress (comparator present)						
6.	Overall	0.001	48%	No effect	—	S51
Randomization						
7.	RCT	0.024	43.3%	Moszeik et al. [68] (11 min) Moszeik et al. [68] (30 min) Rajagopalan et al. [62] Wahbeh and Nelson [40]	0.61 0.12 0.09 0.05	S52
8.	NRCT	0.009	27.1%	Kumar [101]	0.12	S53
Population						
9.	Healthy (adults)	0.008	22.7%	No effect	—	S54

Between group: Anxiety (no comparator)						
10.	Overall	<0.001	77.7%	No effect	—	S55
Randomization						
11.	RCT	<0.001	81.5%	No effect	—	S56
12.	NRCT	0.013	18.1%	No effect	—	S57
Population						
13.	Healthy (adults)	<0.001	82.7%	No effect	—	S58
14.	Healthy (adolescents)	<0.001	0%	No effect	—	S59

Between group: Anxiety (comparator present)						
15.	Overall	<0.001	37.4%	No effect	—	S60
Randomization						
16.	RCT	<0.001	33.9%	No effect	—	S61
17.	NRCT	0.062	0%	Manik and Gartia [74]	0.007	S62
Population						
18.	Healthy (adults)	0.004	17.8%	No effect	—	S63
19.	Menstrual disorder	0.060	1.6%	Rani et al. [65]	<0.001	S64
20.	Hypertension	<0.001	0%	No effect	—	S65

Between group: Depression (no comparator)						
21.	Overall	0.001	77.3%	No effect	—	S66
Randomization						
22.	RCT	0.013	79.6%	Moszeik et al. [68] (11 min) Moszeik et al. [68] (30 min)	0.068 0.064	S67
23.	NRCT	0.001	0%	No effect	—	S68
Population						
24.	Healthy (adults)	0.001	81.7%	No effect	—	S69

Between group: Depression (comparator present)						
25.	Overall	0.007	53.7%	No effect	—	S70
Randomization						
26.	RCT	<0.001	47.7%	No effect	—	S71
27.	NRCT	0.404	0%	Magnúsdóttir et al. [42]	0.309	S72
Population						
28.	Menstrual disorder	<0.001	5.6%	No effect	—	S73
29.	Healthy (adults)	0.037	58.7%	Moszeik et al. [68] (11 min) Moszeik et al. [68] (30 min) Gunjiganvi et al. [36]	0.088 0.082 0.054	S74

Within group: Stress						
30.	Overall	<0.001	72.6%	No effect	—	S75
Randomization						
31.	RCT	<0.001	86%	No effect	—	S76
32.	NRCT	<0.001	26.3%	No effect	—	S77
Population						
33.	Healthy (adults)	<0.001	84%	No effect	—	S78
34.	Healthy (adolescents)	<0.001	7.5%	No effect	—	S79
35.	Hypertension	0.070	0%	Tanna and Khatri [76]	<0.001	S80
				Rajagopalan et al. [62]	<0.001	
36.	Cancer	0.048	0%	No effect	—	S81

Within group: Anxiety						
37.	Overall	<0.001	64.8%	No effect	—	S82
Randomization						
38.	RCT	<0.001	82.4%	No effect	—	S83
39.	NRCT	<0.001	8.6%	No effect	—	S84
Population						
40.	Healthy (adults)	<0.001	77.6%	No effect	—	S85
41.	Healthy (adolescents)	<0.001	0%	No effect	—	S86
42.	Menstrual disorders	0.001	0%	Rani et al. [69]	0.073	S87
43.	Insomnia	0.198	0%	Sharpe et al. [111]	<0.001	S88

Within group: Depression						
44.	Overall	<0.001	69.6%	No effect	—	S89
Randomization						
45.	RCT	<0.001	79.4%	No effect	—	S90
46.	NRCT	0.001	46.7%	No effect	—	S91
Population						
47.	Healthy (adults)	<0.001	79.6%	No effect	—	S92
48.	Healthy (adolescents)	0.997	0%	No effect	—	S93
49.	Depression	0.040	0%	No effect	—	S94
50.	Menstrual disorders	0.116	0%	Rani et al. [65]	<0.001	S95

Abbreviations: NRCTs, nonrandomized controlled trials; RCTs, randomized controlled trials.

## 6 Discussion

This systematic review and meta‐analysis of 73 studies provides comprehensive evidence of the beneficial effects of YN on stress, anxiety, and depression. Notably, the meta‐analysis conducted in this review reports moderate‐to‐large effect size improvements in the between‐group comparisons, both when YN was measured against an active comparator (stress: −0.80, anxiety: −1.35, depression: −0.69) and against no comparator (stress: −1.70, anxiety: −1.43, depression: −0.92). These findings are further supported by within‐group analyses, which also demonstrate moderate‐to‐large effect improvements in stress (−1.05), anxiety, (−1.03), and depression (−0.71). Although leave‐one‐out sensitivity analyses confirmed the robustness of these pooled estimates, these effect sizes should be interpreted with considerable caution, given the generally low methodological quality in most included studies, which suggest a strong likelihood of effect inflation and potential overstatement of YN's true benefits.

### 6.1 Comparison With Previous Literature

To our knowledge, this is the first meta‐analysis to quantitatively synthesize the effects of YN specifically on stress, anxiety, and depression. While several recent reviews have examined the effects of YN on mental health outcomes, including a systematic review [43], a narrative review [124], and an integrative review [125], these studies provided qualitative syntheses without pooled effect size estimates or meta‐analytic quantification. Our meta‐analysis of 73 studies, therefore, represents an advancement in the evidence base, offering the first quantitative assessment of YN's efficacy across these critical mental health domains.

Beyond the YN‐specific literature, our findings align with previous meta‐analyses that examined the effects of modern yoga postures and meditation on these outcomes [126, 127, 128, 129, 130]. However, a key distinction lies in the magnitude of effect, while prior meta‐analyses report small‐to‐large effect size improvements with yoga‐ and meditation‐based interventions in between‐group analyses, our study with YN consistently observed moderate‐to‐large effect size improvements in stress, anxiety, and depression against both active and passive comparators. This apparent discrepancy warrants careful interpretation within the broader context of psychotherapeutic intervention research.

In the field of mental health research, small‐to‐moderate effects are generally considered to be clinically meaningful, as they can generate substantial public health benefits, particularly when implemented on a large scale [131]. For instance, gold‐standard interventions such as cognitive behavioral therapy for depression (Hedges' *g*: = 0.79) [132], and mindfulness‐based stress reduction for psychological distress (SMD: −0.32) [130], demonstrate effect sizes considerably smaller than those observed in our YN meta‐analysis, yet remain cornerstones of evidence‐based mental health treatment [133]. In comparison, while our review demonstrates moderate‐to‐large effect improvements, it also documents several methodological limitations, including a high risk of bias across most of the included studies and substantial heterogeneity in intervention protocols and fidelity reporting. These limitations suggest that the observed effect sizes are likely inflated and do not accurately reflect the true therapeutic effect of YN. Nevertheless, this does not diminish YN's potential clinical value. Even if future, rigorously designed trials reveal more modest effects, comparable to those of established interventions (i.e., in the small‐to‐moderate range), such findings would still represent meaningful therapeutic benefits for an accessible, low‐cost intervention like YN.

### 6.2 Study Designs

An important aspect in this review was that the inclusion criteria were deliberately kept broad regarding the study designs. First, we aimed to incorporate all available evidence on YN's effects to provide a comprehensive overview of the literature. Second, recognizing the relative scarcity of RCTs in this area, we sought to enhance the statistical power of our meta‐analysis by including a wider range of study designs. This approach also enabled well‐powered within‐group analyses, allowing for a more nuanced assessment of YN's overall effects from both intraindividual and interindividual perspectives. Importantly, the findings from these within‐group analyses complemented the between‐group comparisons by helping us assess whether the direction and magnitude of individual‐level changes aligned with those observed in controlled studies. Furthermore, to mitigate potential biases inherent in nonrandomized and quasi‐experimental studies, we conducted subgroup analyses to isolate the intervention's effects within randomized designs, revealing important distinctions based on study methodology.

To illustrate these distinctions, the between‐group subgroup analyses revealed significant reductions across multiple outcomes in RCTs. Specifically, when compared against active controls, these included improvements in stress (−0.86, *p* = 0.024), anxiety (−1.62, *p*<0.001), and depression (−0.98, *p*<0.001). Similar patterns emerged in RCTs using no‐comparator controls, with significant reductions in stress (−1.97, *p*<0.001), anxiety (−2.32, *p*<0.001), and depression (−1.38, *p* = 0.013). These findings were further corroborated by the within‐group analyses, which included a larger pool of studies than the between‐group analyses, potentially increasing statistical power. When stratified by study design, these within‐group comparisons showed that RCT‐based subgroup analyses aligned consistently with the overall pooled analysis for stress (−1.79, *p*<0.001), anxiety (−1.17, *p*<0.001), and depression (−1.04, *p*<0.001), suggesting that the overall outcomes observed for stress, anxiety, and depression remained consistent across both study design stratifications and analysis types (between‐group and within‐group).

However, an important limitation to consider when interpreting these results is the generally poor methodological quality of the included randomized and nonrandomized studies, indicating a high risk of bias. For instance, assessment using the Cochrane ROB2 tool revealed that only five studies were rated as having “some concerns” regarding bias, while the remaining majority (i.e., 77%) of RCTs were classified as having a high risk of bias (Table 1). The key methodological issues identified in the RCTs were related to bias in outcome measurement, which occurred due to inadequate reporting of assessor blinding; bias in the selection of reported results, which arose primarily due to the absence of preregistered protocols; and bias due to deviation from intended interventions, which occurred primarily due to inadequate blinding of participants and intervention providers, all of which could compromise the reliability and validity of findings. Similar limitations have been noted in previous reviews assessing the effectiveness of yoga‐based interventions [127, 128]. Collectively, these methodological weaknesses highlight the need for more rigorous RCTs to establish the true effectiveness of YN.

### 6.3 YN and Different Health Conditions

This review included diverse population groups with varying health conditions to establish clear clinical guidelines for the implementation of YN. Notably, the majority of studies focused on healthy individuals, particularly adults and adolescents, while those with clinical conditions, such as menstrual disorders, cancer, and insomnia, among others, were studied to a lesser extent (Table 3). Even more striking was the limited research on individuals with diagnosed anxiety and depressive disorders, with only two studies evaluating each condition. This disparity in the studies’ target groups also influenced the meta‐analysis. For example, in between‐group analyses of stress, subgroup analysis was only feasible for healthy adults and adolescents. No subgroup analyses were conducted for other populations, such as those with depression or hypertension, due to a lack of multiple studies. Only in the anxiety analyses were subgroup assessments conducted on individuals with hypertension and menstrual disorders, alongside healthy adults. Furthermore, in the within‐group analyses, subgroup analyses were conducted on multiple patient population groups because of a larger pool of included studies. Here, for stress, in addition to healthy adults (−0.98, *p*<0.001) and adolescents (−1.07, *p*<0.001), subgroup analyses showed a significantly large effect improvement in stress for individuals with cancer (−2.20, *p* = 0.048), while a nonsignificant improvement was observed for those with hypertension (−2.48, *p* = 0.07). For anxiety, subgroup analyses of individuals with menstrual disorders (−0.46, *p* = 0.001), along with healthy adults (−1.18, *p*<0.001) and adolescents (−0.76, *p*<0.01), reported significant improvements, whereas no significant effect was observed for those with insomnia (−1.18, *p* = 0.198). For depression, subgroup analyses revealed significant improvements in individuals with diagnosed depressive disorders (−1.16, *p* = 0.04) and healthy adults (−0.93, *p*<0.001). However, no significant effect was observed for individuals with menstrual disorders (−0.49, *p* = 0.116) or healthy adolescents (−0.002, *p* = 0.997).

One study evaluating the effects of YN on depression specifically attributed the observed improvements due to YN on two key factors [40]. First, the authors suggested that the emotional regulation component of YN may have helped participants cultivate positive emotions and recall them even in stressful situations, fostering a more responsive rather than reactive approach to challenges. Second, they proposed that the group‐based nature of the sessions could have promoted a sense of connection and reduced loneliness among the depressed participants. These findings align with a study by Foulkrod et al. [89], which reported reduced depression outcomes when YN was combined with talk therapy. Additionally, a qualitative study by Stankovic [119] on combat veterans with post‐traumatic stress disorder further emphasized the role of emotional regulation in YN. The study suggested that YN might function similarly to exposure therapy by encouraging confrontation and re‐experiencing of traumatic memories. Furthermore, according to the author, visualization exercises focusing on pleasure, strength, and resilience could also have helped counteract states of anxiety, hypervigilance, and rage, which are common in posttraumatic stress disorder. Additionally, interpretations can be drawn from traditional yogic frameworks regarding YN's therapeutic mechanisms. Satyananda [16] explained that by inducing pratyāhāra (i.e., withdrawal of sensory awareness) through techniques such as rotation of consciousness throughout the body, visualization, and breath awareness [30], YN can help in liberating excess energy that would conventionally be bound to external stimuli causing nervous depletion and even breakdown, and by redirecting it toward healing and rejuvenation of over‐taxed physiological systems. From this perspective, the substantial reductions in stress, anxiety, and depression observed in our meta‐analysis may reflect a somatopsychic healing process wherein psychosomatic imbalances are spontaneously restored through the withdrawal of awareness from sensory channels.

Besides patient population groups, an important consideration in the subgroup analysis of the healthy population is the variation in participants’ occupations, which could have influenced their stress, anxiety, and depression levels. For example, studies on healthy adults assessed the effects of YN among healthcare practitioners [79, 92], healthcare workers working during the COVID‐19 pandemic [36], athletes [63], military personnel [113], students [42, 87], teachers [38], and others (see Table S3). While a detailed subgroup analysis within this category was not conducted, the overall findings indicate that YN significantly reduces stress, anxiety, and depression among healthy adults, suggesting its potential as a preventive tool for managing everyday psychological distress. Specifically, Eastman‐Mueller et al. [87] reported significant reductions in stress, depression, and worry among college students, attributing these improvements to enhanced mindfulness skills such as nonreactivity, emotional awareness, and nonjudgment with YN. These improvements may have helped students better regulate their responses to stressful situations, ultimately improving their psychological well‐being. Similarly, Jensen [93] found that YN stabilized respiratory patterns in students with behavioral dysfunction, likely due to its stimulation of the parasympathetic nervous system [134], which may have contributed to improved behavioral outcomes. Additionally, other studies highlighted YN's positive effects on physiological stress markers, such as cortisol [68], as well as on improvements in performance [39], physical recovery [71], sleep [59], and cognitive function [135]. Overall, these findings suggest that the benefits of practicing YN may enhance an individual's ability to manage daily activities more effectively while also promoting their overall psychological well‐being.

### 6.4 Heterogeneity in YN

A key objective of this review was to examine how YN had been delivered in existing literature to help standardize reporting methods, which is essential for both clinical implementation and the generalizability of research findings. We identified significant inconsistencies in implementation protocols across studies. For instance, only 52.8% of studies provided information about the structure of YN interventions, specifically which steps were followed and in what sequence. More concerning was that 30% of studies failed to specify whether they followed a particular established approach to YN, such as outlined by the Bihar School of Yoga or iRest. These omissions raise fundamental questions about the validity of findings and their applicability in research and clinical contexts. Another critical inconsistency was that 30% of studies did not report the presence of a trained instructor to guide YN sessions. As YN is typically an instructor‐guided intervention, the absence of a qualified instructor raises serious concerns about intervention fidelity and effectiveness. Moreover, since YN involves visualization of memories and emotions, improper guidance could potentially have retraumatizing effects [136]. Therefore, the presence of trained instructors well‐versed in guiding practitioners through such experiences is essential for ensuring psychological safety.

Additionally, we observed substantial variability in reporting training dosages. While 90% of studies reported dosage information, the range of training duration varied dramatically, that is, from a single 30‐min session to 4550 min of training delivered across 26 weeks (five 35‐min sessions weekly). Such wide variation complicates standardization and comparability of interventions, making it difficult to establish clear dosage−response relationships and derive clinically meaningful conclusions.

### 6.5 Limitations

Despite its novelty, this study had a few limitations. Our deliberately broad inclusion criteria encompassed diverse research designs, ranging from RCTs to case studies and even a qualitative investigation. This inclusive approach served dual purposes: to present a comprehensive overview of the existing literature (thereby increasing statistical power for between and within‐group meta‐analyses) while thoroughly documenting YN implementation characteristics across various contexts. To address the inherent methodological heterogeneity, we conducted stratified subgroup analyses that separated RCTs from nonrandomized studies, respecting the established hierarchy of evidence. Nevertheless, this methodological diversity may have introduced variability in evidence quality and type, potentially affecting the robustness and generalizability of our findings.

Second, in alignment with this inclusive strategy, we also employed a deliberately broad search approach centered around the intervention (YN) itself rather than a restrictive strategy based on a PICOS‐based framework. This was done to maximize sensitivity and capture all potentially relevant studies, acknowledging the limited and heterogeneous nature of the available literature. Although this approach increased the screening workload, it minimized the risk of omitting pertinent studies that used varied terminology or were situated in diverse contexts. This decision, while necessary for comprehensiveness, may have further contributed to heterogeneity in study quality and outcome reporting. Third, a few of the subgroup meta‐analyses carried out to evaluate the effects of YN had included only two studies. The limited number of studies included in the analysis could raise concerns about the generalizability and reliability of the subgroup results. Nonetheless, in order to ensure the robustness of the results, leave‐one‐out sensitivity analyses were carried out to assess the impact of individual studies on the overall findings. Fourth, a lack of standardization in the outcome measures across the included studies might have also contributed to inconsistencies in the reported findings. For instance, in the evaluation of stress, only there were 21 different outcome indicators involved. This variability in how outcomes were measured could complicate the interpretation of the effects, as different scales capture different aspects of the same construct. Fifth, another limitation was that this study did not ascertain the long‐term effects of YN. Long‐term data are crucial for understanding the sustained impact of interventions and their potential for lasting improvements in participants’ health and well‐being. This was not done specifically because of the dearth of data, as only two studies had reported long‐term follow‐up for between‐group comparisons, that is, for 6 and 12 weeks.

### 6.6 Future Directions

This review highlights important inconsistencies in the current evidence that must be addressed in future research to determine the true clinical implications of YN for managing stress, anxiety, and depression. First, there is a need for high‐quality, adequately powered RCTs evaluating YN's effects on these psychological outcomes. Such rigorous assessment would enable researchers and clinicians to confirm and draw reliable conclusions about its effectiveness.

Second, we recommend that future research evaluate YN effects in populations with clinically diagnosed depression and anxiety conditions. This would facilitate the development of clinical care guidelines for implementing YN in mental health treatment. Third, future investigations should consistently explore long‐term effects of YN while emphasizing thorough reporting of implementation parameters, that is, YN steps, instructor presence, and the specific type of YN used. Such measures would refine our understanding of YN's therapeutic potential and assist in developing evidence‐based protocols for clinical application.

### 6.7 Clinical Relevance

Given the substantial heterogeneity in study quality and the high risk of bias identified across most included studies, clinicians and healthcare practitioners must exercise considerable caution when interpreting these findings for clinical practice. Although the overall moderate‐to‐large effect sizes from our meta‐analyses are encouraging, the pervasive methodological bias and heterogeneity in the included studies suggest these estimates are likely overstated and do not accurately reflect YN's true therapeutic benefits. Consequently, at this stage, the most prudent conclusion for clinical application is that YN may serve as a complementary approach for individuals experiencing mild‐to‐moderate stress, anxiety, or depression, particularly as an adjunct to established interventions. The consistency of positive trends across multiple study designs and populations, despite methodological limitations, provides preliminary support for this cautious recommendation.

That said, YN should not be positioned as a replacement for established first‐line treatments, as the current evidence cannot resolve key uncertainties regarding its comparative efficacy, long‐term durability, or underlying mechanisms. Moreover, the absence of standardized protocols hinders practical implementation, leaving ambiguity about which YN elements (e.g., body scanning, visualization, emotional regulation) are core to its benefits and which are peripheral. Until these deficiencies are rectified through high‐quality research, YN's clinical use should be exploratory and patient‐tailored, incorporating vigilant monitoring and embedding within holistic care frameworks that prioritize evidence‐based therapies where indicated.

## 7 Conclusion

This review examines the evidence supporting YN's beneficial effects on stress, anxiety, and depression. Our overall between‐group findings reveal significant improvements when comparing YN to both active interventions and no comparator controls. Subgroup analyses of RCTs demonstrated either significant improvements or positive trends across all three conditions. Additionally, moderate‐to‐large effect improvements were observed in both healthy individuals and patient populations, suggesting YN's potential applicability across diverse groups. Within‐group analyses further validated these findings, showing significant improvements in stress, anxiety, and depression outcomes.

However, this review also identified important limitations in the current literature, especially concerning methodological quality, the risk of inflated effect sizes, and inconsistent reporting of intervention fidelity. These issues limit the strength and generalizability of the conclusions and hinder our ability to provide firm implementation recommendations.

Taking these limitations into account, the current evidence suggests that YN may hold promise as a complementary approach for reducing stress, anxiety, and depression. Further high‐quality, rigorously controlled studies with transparent reporting of YN protocols are needed to confirm YN's effectiveness and clarify its role in comprehensive treatment plans for individuals seeking relief from psychological disorders.

## Author Contributions

S.G. and P.O. conceptualized the study. S.G. and I.G. independently screened the articles, assessed the methodological quality of the studies, and conducted the meta‐analysis. S.G. drafted the initial manuscript. All authors reviewed and approved the final version.

## Funding

Open access funding was provided by Karlstad University.

## Conflicts of Interest

The authors declare that they have no competing interests.

## Supporting information




**Supplementary material**: nyas70149‐sup‐0001‐SuppMat.docx

**Supplementary material**: nyas70149‐sup‐0002‐SuppMat.pdf
