# Supplementary material for: Effects of Yoga Nidra on Stress, Anxiety, and Depression: A Systematic Review and Meta‐Analysis
Source: Ann N Y Acad Sci. 2025 Dec 1;1556(1):e70149. doi: 10.1111/nyas.70149 (PMC12917937; doi:10.1111/nyas.70149)
Supplement: Supplementary file 1 — Supplementary material: nyas70149‐sup‐0001‐SuppMat.docx [file NYAS-1556-0-s001.docx]

**Effects of Yoga Nidra on Stress, Anxiety, and Depression: A Systematic Review and A Between- and Within-Group Meta-analysis**

**Supplementary file**

Table S1. PRISMA checklist

| **Section and Topic** | **Item #** | **Checklist item** | **Location where item is reported** |
| --- | --- | --- | --- |
| **TITLE** | | |  |
| Title | 1 | Identify the report as a systematic review. | 1 |
| **ABSTRACT** | | |  |
| Abstract | 2 | See the PRISMA 2020 for Abstracts checklist. | 1 |
| **INTRODUCTION** | | |  |
| Rationale | 3 | Describe the rationale for the review in the context of existing knowledge. | 1-2 |
| Objectives | 4 | Provide an explicit statement of the objective(s) or question(s) the review addresses. | 2-3 |
| **METHODS** | | |  |
| Eligibility criteria | 5 | Specify the inclusion and exclusion criteria for the review and how studies were grouped for the syntheses. | 3 |
| Information sources | 6 | Specify all databases, registers, websites, organisations, reference lists and other sources searched or consulted to identify studies. Specify the date when each source was last searched or consulted. | 3 |
| Search strategy | 7 | Present the full search strategies for all databases, registers and websites, including any filters and limits used. | 3, Table S2 |
| Selection process | 8 | Specify the methods used to decide whether a study met the inclusion criteria of the review, including how many reviewers screened each record and each report retrieved, whether they worked independently, and if applicable, details of automation tools used in the process. | 3-4 |
| Data collection process | 9 | Specify the methods used to collect data from reports, including how many reviewers collected data from each report, whether they worked independently, any processes for obtaining or confirming data from study investigators, and if applicable, details of automation tools used in the process. | 5 |
| Data items | 10a | List and define all outcomes for which data were sought. Specify whether all results that were compatible with each outcome domain in each study were sought (e.g. for all measures, time points, analyses), and if not, the methods used to decide which results to collect. | 5 |
|  | 10b | List and define all other variables for which data were sought (e.g. participant and intervention characteristics, funding sources). Describe any assumptions made about any missing or unclear information. | 5 |
| Study risk of bias assessment | 11 | Specify the methods used to assess risk of bias in the included studies, including details of the tool(s) used, how many reviewers assessed each study and whether they worked independently, and if applicable, details of automation tools used in the process. | 5 |
| Effect measures | 12 | Specify for each outcome the effect measure(s) (e.g. risk ratio, mean difference) used in the synthesis or presentation of results. | 5 |
| Synthesis methods | 13a | Describe the processes used to decide which studies were eligible for each synthesis (e.g. tabulating the study intervention characteristics and comparing against the planned groups for each synthesis (item #5)). | 5, Table S3 |
|  | 13b | Describe any methods required to prepare the data for presentation or synthesis, such as handling of missing summary statistics, or data conversions. | 5 |
|  | 13c | Describe any methods used to tabulate or visually display results of individual studies and syntheses. | 5 |
|  | 13d | Describe any methods used to synthesize results and provide a rationale for the choice(s). If meta-analysis was performed, describe the model(s), method(s) to identify the presence and extent of statistical heterogeneity, and software package(s) used. | 5 |
|  | 13e | Describe any methods used to explore possible causes of heterogeneity among study results (e.g. subgroup analysis, meta-regression). | 5 |
|  | 13f | Describe any sensitivity analyses conducted to assess robustness of the synthesized results. | 5 |
| Reporting bias assessment | 14 | Describe any methods used to assess risk of bias due to missing results in a synthesis (arising from reporting biases). | 5 |
| Certainty assessment | 15 | Describe any methods used to assess certainty (or confidence) in the body of evidence for an outcome. | - |
| **RESULTS** | | |  |
| Study selection | 16a | Describe the results of the search and selection process, from the number of records identified in the search to the number of studies included in the review, ideally using a flow diagram. | 6 |
|  | 16b | Cite studies that might appear to meet the inclusion criteria, but which were excluded, and explain why they were excluded. | 6, Figure 1 |
| Study characteristics | 17 | Cite each included study and present its characteristics. | 6-15 |
| Risk of bias in studies | 18 | Present assessments of risk of bias for each included study. | 6-10 |
| Results of individual studies | 19 | For all outcomes, present, for each study: (a) summary statistics for each group (where appropriate) and (b) an effect estimate and its precision (e.g. confidence/credible interval), ideally using structured tables or plots. | 17-26 |
| Results of syntheses | 20a | For each synthesis, briefly summarise the characteristics and risk of bias among contributing studies. | 6-10, 17-23 |
|  | 20b | Present results of all statistical syntheses conducted. If meta-analysis was done, present for each the summary estimate and its precision (e.g. confidence/credible interval) and measures of statistical heterogeneity. If comparing groups, describe the direction of the effect. | 17-26 |
|  | 20c | Present results of all investigations of possible causes of heterogeneity among study results. | 17-26 |
|  | 20d | Present results of all sensitivity analyses conducted to assess the robustness of the synthesized results. | Table 6, 24-26 |
| Reporting biases | 21 | Present assessments of risk of bias due to missing results (arising from reporting biases) for each synthesis assessed. | 15-16, Figure 2 |
| Certainty of evidence | 22 | Present assessments of certainty (or confidence) in the body of evidence for each outcome assessed. | - |
| **DISCUSSION** | | |  |
| Discussion | 23a | Provide a general interpretation of the results in the context of other evidence. | 28 |
|  | 23b | Discuss any limitations of the evidence included in the review. | 28-31 |
|  | 23c | Discuss any limitations of the review processes used. | 31 |
|  | 23d | Discuss implications of the results for practice, policy, and future research. | 31-32 |
| **OTHER INFORMATION** | | |  |
| Registration and protocol | 24a | Provide registration information for the review, including register name and registration number, or state that the review was not registered. | 4 |
|  | 24b | Indicate where the review protocol can be accessed, or state that a protocol was not prepared. | 4 |
|  | 24c | Describe and explain any amendments to information provided at registration or in the protocol. | 4 |
| Support | 25 | Describe sources of financial or non-financial support for the review, and the role of the funders or sponsors in the review. | 33 |
| Competing interests | 26 | Declare any competing interests of review authors. | 33 |
| Availability of data, code and other materials | 27 | Report which of the following are publicly available and where they can be found: template data collection forms; data extracted from included studies; data used for all analyses; analytic code; any other materials used in the review. | 33 |

*From:*  Page MJ, McKenzie JE, Bossuyt PM, Boutron I, Hoffmann TC, Mulrow CD, et al. The PRISMA 2020 statement: an updated guideline for reporting systematic reviews. BMJ 2021;372:n71. doi: 10.1136/bmj.n71

For more information, visit: <http://www.prisma-statement.org/>Table S2. Search strategy used across different databases

| Database | Search string | Number of articles |
| --- | --- | --- |
| Pubmed | "Yoga nidra" OR yoganidra OR "iRest meditation" OR "Integrative restoration" OR "yogic sleep" | 78 |
| CINAHL | "Yoga nidra" OR yoganidra OR "iRest meditation" OR "Integrative restoration" OR "yogic sleep" | 37 |
| Psychinfo | "Yoga nidra" OR yoganidra OR "iRest meditation" OR "Integrative restoration" OR "yogic sleep" | 33 |
| Scopus | "Yoga nidra" OR yoganidra OR "iRest meditation" OR "Integrative restoration" OR "yogic sleep" | 122 |
| SPORTDiscus | "Yoga nidra" OR yoganidra OR "iRest meditation" OR "Integrative restoration" OR "yogic sleep" | 39 |
| Web of Science | "Yoga nidra" OR yoganidra OR "iRest meditation" OR "Integrative restoration" OR "yogic sleep" | 87 |
| ProQuest | "Yoga nidra" OR yoganidra OR "iRest meditation" OR "Integrative restoration" OR "yogic sleep" | 263 |
| Cochrane | "Yoga nidra" OR yoganidra OR "iRest meditation" OR "Integrative restoration" OR "yogic sleep" | 124 |

Table S3. Details of included studies

| Study (year) | Country | Design | Sample details  Male, Female (Age)  Health status | Intervention details | Training dosage | Yoga nidra specifications | Assessment period | Comparator | Outcomes | Result | Quality |
| --- | --- | --- | --- | --- | --- | --- | --- | --- | --- | --- | --- |
| Moszeik et al. (2025) | Germany | RCT | YGN 1: 79F, 18M, 1 diverse (19 to 80)  YGN 2: 68F, 11M (19 to 80)  Control 1: 54F, 17M (19 to 80)  Control 2: 71F, 18M (19 to 80)  Healthy | YGN 1: 11 minutes yoga nidra | YGN 1:  Duration of YGN: 11 minutes  Number of days: 57 | YGN 1: Delivery setting: at-home (audio recordings)  Trained instructor: Yes  Steps: Four steps included: i) brief body scan; ii) observation of breath for approx. 20 seconds; iii) setting a personal intention (Sankalpa); iv) observation of thoughts and feelings | Pre-intervention  Post-intervention  Follow-up (3 months) | Control 1: Music intervention (Santulan Om meditation as described by També (2016))  Control 2: Waitlist control | Trier inventory for chronic stress 12-item  State-Trait Anxiety and Depression inventory  Satisfaction with life scale  Rumination scale from Rumination-Reflection questionnaire  Pittsburg Sleep Quality Index  Five-facet mindfulness questionnaire  Cortical awakening response  Diurnal flattening  Wellbeing cognitive and affective components based on Diener et al. (1999)  Intervention fidelity | **Between-group:**  YGN (11-min) vs. Control 1:  **Post-intervention:**  Significant ↓ in depression (State trait depression inventory) with YGN as compared to compared to Control 1  No difference in stress (Trier inventory for chronic stress) with YGN as compared to compared to Control 1  No difference in anxiety (State trait anxiety inventory) with YGN as compared to compared to Control 1  No differences significant for other outcomes with YGN as compared to Control 1  **3-month follow up:**  Probability that follow-up effect negates interaction below 99.64% suggesting observed effects not maintained at 3-month follow up with YGN as compared to Control 1  YGN (11-min) vs. Control 2:  **Post-intervention:**  Significant ↓ in stress (Trier inventory for chronic stress) with YGN as compared to compared to Control 2  Significant ↓ in anxiety (State trait anxiety inventory) with YGN as compared to compared to Control 2  Significant ↓ in depression (State trait depression inventory) with YGN as compared to compared to Control 2  Significant ↓ in rumination (Rumination reflection questionnaire) with YGN as compared to compared to Control 2  Significant ↑ in non-reactivity and acceptance without judgement subsets of mindfulness with YGN as compared to Control 2.  No differences significant for other outcomes with YGN as compared to Control 2.  **3-month follow up:**  Probability that follow-up effect negates interaction below 9.26% suggesting observed effects maintained at 3-month follow up with YGN as compared to Control 2  Implementation fidelity: Participants that attended YGN training sessions  All 57: 6, 39-56: 16, 21-38: 17, 9-20: 13, 2-8: 7, 1: 3, 0: 0  **Within group:**  **Post-intervention:**  ↓ in stress (Trier inventory for chronic stress) with YGN post-intervention  ↓ in anxiety (State trait anxiety inventory) with YGN post-intervention  ↓ in depression (State trait depression inventory) with YGN post-intervention  **3-month follow-up:**  ↓ in stress (Trier inventory for chronic stress) with YGN at follow-up  ↓ in anxiety (State trait anxiety inventory) with YGN at follow-up  ↓ in depression (State trait depression inventory) with YGN at follow-up | **RoB2: Some concern** |
|  |  |  |  | YGN 2: 30 minutes yoga nidra | YGN 2:  Duration of YGN: 30 minutes  Number of days: 57 | YGN 2: Delivery setting: at-home (audio recordings)  Trained instructor: Yes  Steps: Six steps included: i) extended body scan; ii) extended observation of breath; iii) setting a personal intention (Sankalpa); iv) observation of thoughts and feelings; v) chakra perception; vi) balancing sensations |  |  |  | **Between-group:**  YGN (30-min) vs. Control 1:  **Post-intervention:**  Significant ↓ in depression (State trait depression inventory) with YGN as compared to compared to Control 1  Significant ↓ in stress (Trier inventory for chronic stress) with YGN as compared to compared to Control 1  Significant ↓ in anxiety (State trait anxiety inventory) with YGN as compared to compared to Control 1  Significant ↓ in cortisol awakening response with YGN as compared to compared to Control 1  No differences significant for other outcomes with YGN as compared to Control 1  **3-month follow up:**  Probability that follow-up effect negates interaction below 5.29% for stress, depression and rumination suggesting observed effects maintained at 3-month follow up with YGN as compared to Control 1, however, for cortisol awakening response the probability is 41.5% suggesting that the effect is less stable at follow-up  YGN (30-min) vs. Control 2:  **Post-intervention:**  Significant ↓ in stress (Trier inventory for chronic stress) with YGN as compared to compared to Control 1  Significant ↓ in depression (State trait depression inventory) with YGN as compared to compared to Control 2  Significant ↓ in anxiety (State trait anxiety inventory) with YGN as compared to compared to Control 2  Significant ↓ in rumination (Rumination reflection questionnaire) with YGN as compared to compared to Control 2  Significant ↓ in sleep disturbance with YGN as compared to compared to Control 2  Significant ↓ in cortisol awakening response with YGN as compared to compared to Control 2  Significant ↑ in facet mindfulness with YGN as compared to Control 2  No differences significant for other outcomes with YGN as compared to Control 2  **3-month follow up:**  Probability that follow-up effect negates interaction below 11.87% for stress, anxiety, depression, rumination, sleep disturbance, and cortisol awakening response suggesting observed effects maintained at 3-month follow up with YGN as compared to Control 2  Implementation fidelity: Participants that attended YGN training sessions  All 57: 0, 39-56: 19, 21-38: 10, 9-20: 17, 2-8: 12, 1: 1, 0: 3  **Within group:**  **Post-intervention:**  ↓ in stress (Trier inventory for chronic stress) with YGN post-intervention  ↓ in anxiety (State trait anxiety inventory) with YGN post-intervention  ↓ in depression (State trait depression inventory) with YGN post-intervention  **3-month follow-up:**  ↓ in stress (Trier inventory for chronic stress) with YGN at follow-up  ↓ in anxiety (State trait anxiety inventory) with YGN at follow-up  ↓ in depression (State trait depression inventory) with YGN at follow-up |  |
| Shivaji and Dnyeshwar (2025) | India | Quasi experimental design (pre vs post) | 34F, 16M (≥ 60)  Older adults | YGN: Yoga nidra | Duration of YGN: 20 minutes  Number of days: 15  Number of sessions: 15 | Delivery setting: In-person  Trained instructor: NR  Steps: NR | Pre-intervention  Post-intervention | NA | DASS-21 | **Between-group:** NA  **Within-group:** Significant ↓ in anxiety (DASS-21) with YGN post-intervention | **D&B: Fair** |
| Magnúsdóttir et al. (2025) | Iceland | Non-randomized controlled trial | YGN: 19F, 9M (17 ± 0.1)  Control: 28F, 9M (17.5 ± 0.1)  Healthy (adolscents) | YGN: Yoga nidra + breathing exercises (Nadi shodhna) | YGN:  Duration of YGN: 60 minutes  Number of days per week: 2  Number of weeks: 4  Control: - | Delivery setting: In-person  Trained instructor: Yes  Steps: NR | Pre-intervention  Post-intervention | Control: Sleep education | GAD-7  BDI-II  Epsworth sleepiness index  Insomnia severity index  Morning eveningness questionnaire  Sleep diary  Sleep duration  Sleep (onset, mid-point, conclusion)  Social jetlag  Sleep quality index  Total sleep time  Wake after sleep onset  Rapid eye movement sleep  Unstable/stable sleep  Sleep efficiency  Apnea Hypopnea index  Attrition | **Between-group:**  No difference in anxiety (GAD-7) with YGN as compared to control  No difference in depression (BDI-II) with YGN as compared to control  No difference in Epsworth sleepiness index with YGN as compared to control  No difference in Insomnia severity index with YGN as compared to control  No difference in Morning eveningness questionnaire  with YGN as compared to control  No difference in average sleep duration with YGN as compared to control  Significant ↓ in sleep (onset, mid-point, conclusion) with YGN as compared to control  Significant ↓ in social jetlag with YGN as compared to control  No difference in total sleep time with YGN as compared to control  No difference in wake after sleep onset with YGN as compared to control  No difference in sleep quality index with YGN as compared to control  No difference in sleep efficiency with YGN as compared to control  No difference in stable/unstable with YGN as compared to control  No difference in rapid eye movement sleep with YGN as compared to control  Attrition: drop out YGN (n= 3), control (n= 4), and three additional participants in control group had no data on free-nights  **Within-group:** NR | **D&B: Poor** |
| Panigrahi et al. (2024) | India | Quasi experimental design (pre vs post) | 15F, 30 M (63.1, 55 to 70)  Older adults | Yoga nidra + pranayama (Ujjayi, Nadisodhan, Bhramari, Omkar) | Duration of YGN: 20 minutes  Number of days per week: NR  Number of days: 60  *Total training 50 minutes: Slow paced Ujjayi pranayama: 5 minutes, Nadisodhan pranayama: 5 minutes, Bhramari pranayama: 5 minutes, Omkar pranayama: 5 minutes, YGN: 20 minutes, loosening exercises: 10 minutes) | Delivery setting: In-person, at-home  Trained instructor: Yes  Steps: NR  Guided session given by instructor for 4 days, afterwards participants practiced under supervision, also requested to practice at home | Pre-intervention  Post-intervention | NA | Perceived stress scale  Blood pressure (systolic, diastolic) | **Between-group:** NA  **Within-group:**  Significant ↓ in stress (PSS) with YGN post-intervention  ↓ in blood pressure (systolic and diastolic) with YGN post-intervention (Statistical analysis not reported) | **D&B: Poor** |
| Nuzhath et al. (2024) | India | Randomized controlled trial | YGN: 35F (51.1 ± 10.2)  Control: 35F (53.4 ± 12.0)  Cervical cancer stages IB2 to IVA | YGN: Yoga nidra + standard care | Duration of YGN: 10 + 10 minutes (performed twice daily)  Number of days per week: 5  Number of weeks: 6  *Total training 30 minutes (Breathing exercises: 5 minutes, Pranayama: 15 minutes, YGN: 10 minutes) | Delivery setting: In-person  Trained instructor: Yes  Steps: NR | Pre-intervention  Mid-intervention (week-2, 4)  Post-intervention | Control: Standard care | Hospital anxiety scale  Hospital depression scale | **Between-group:**  Significant ↓ in anxiety (Hospital anxiety scale) with YGN as compared to control group  Significant ↓ in depression (Hospital depression scale) with YGN as compared to control group  **Within-group:**  Significant ↓ in anxiety (Hospital anxiety scale) with YGN post-intervention  Significant ↓ in depression (Hospital depression scale) with YGN post-intervention | **RoB2: High risk** |
| Tanna and Khatri (2024) | India | Quasi experimental design (pre vs post) | 22F, 27M (47.8 ± 10.6)  Hypertension | Yoga nidra | Duration of session: 15 to 20 minutes  Number of days per week: 6  Number of weeks: 2 | Delivery setting: In-person (audio recording)  Trained instructor: Yes  Steps: Six steps included: i) preparedness; ii) resolution; iii) rotation of consciousness; iv) breath awareness; v) feeling and sensation; vi) visualization; vii) resolve; viii) completion | Pre-intervention  Post-intervention | NA | Perceived stress scale  Blood pressure (systolic, diastolic) | **Between-group:** NA  **Within-group:**  Significant ↓ in stress (Perceived stress scale) with YGN post-intervention  Significant ↓ in blood pressure (systolic, diastolic) with YGN post-intervention | **D&B: Fair** |
| Muley et al. (2024) | India | Randomized controlled trial | YGN: 58: ?F, ?M (18 to 5)  Control: 58: ?F, ?M (18 to 25)  Healthy (adults: students) | YGN: Yoga nidra | Duration of session: 35 minutes  Number of sessions per week: 7  Number of weeks: 2 | Delivery setting: In-person (audio recording)  Trained instructor: NR  Steps: NR | Post-intervention | Control: No training | Stress relaxation rating scale (very true, somewhat true, not sure, somewhat untrue, very untrue for 5 categories: felt good, easy to fit in schedule, made me feel relaxed, handled daily chores better than I usually did, easy technique to learn) | **Between-group:**  Significant ↑ in stress relaxation score (Stress relaxation rating scale) reported as very true, and somewhat true reported for all 5 categories with YGN as compared to control  Significant ↓ in stress relaxation score (Stress relaxation rating scale) reported as somewhat untrue, and very untrue for all 5 categories with YGN as compared to control  No difference in stress relaxation score (Stress relaxation rating scale) reported as not sure for all 5 categories with YGN as compared to control  **Within-group:**  NR | **RoB2: High risk** |
| Ravi et al. (2024) | India | Case study | 1F (16)  Idiopathic intracranial hypertension | Yoga nidra | **In-person**:  Duration of session: 45 minutes  Number of days: 10  **Virtual**:  Duration of session: 45 minutes  Number of sessions per week: 3  Number of weeks: 9 | Delivery setting: In-person, virtual  Trained instructor: Yes  Steps: Nine steps included: i) preparation; ii) relaxation; iii) mental affirmation to themselves for personal resolution (Sankalpa); iv) rotation of consciousness; v) awareness of breath; vi) awareness of sensation; vii) inner space; viii) Sankalpa; ix) ending practice | Pre-intervention  Post-intervention | NA | Perceived stress scale  Visual analogue scale for pain  Headache impact test-6  Pittsburg sleep quality index | **Between-group:** NA  **Within-group:**  ↓ in stress (Perceived stress scale) with YGN post-intervention  ↓ in pain (Perceived stress scale) with YGN post-intervention  ↓ in pain headache impact test-6 score with YGN post-intervention  ↓ in Pittsburg sleep quality index with YGN post-intervention | **D&B: Poor** |
| Kumar et al. (2024) | India | Quasi experimental design (pre vs post) | 20F (24 to 35)  Healthy (pregnant women) | Yoga nidra + opening and closing prayers | Duration of session: 20 minutes  Number of days per week:1  Number of weeks: 3 | Delivery setting: NR  Trained instructor: NR  Steps: NR | Pre-intervention  Post-intervention | NA | Sinha comprehensive anxiety test scale | **Between-group:** NA  **Within-group:** Significant ↓ in anxiety (Sinha comprehensive anxiety test scale) with YGN post-intervention | **D&B: Poor** |
| Rajesh et al. (2023) | India | Randomized controlled trial | YGN: 50: ?F, ?M (18 to 24)  Control: 50: ?F, ?M (18 to 24)  Healthy (adults: sports person) | YGN: Yoga nidra | Duration of session: 35 minutes  Number of sessions per week: 7  Number of weeks: 2 | Delivery setting: In-person  Trained instructor: NR  Steps: Followed guidelines outlined by Bihar School of Yoga, individual steps NR | Pre-intervention  Post-intervention | Control: Yoga asanas (Surya namaskar, Paschimotanasana, Dhanurasana, Bhujangasana, Ardhchakrasana, Tadasana) | Ed Fenn Beck anxiety self-rating scale | **Between-group:**  Significant ↓ in anxiety (BAI) with YGN as compared to control  **Within-group:**  Significant ↓ in anxiety (BAI) with YGN post-intervention | **RoB2: High risk** |
| Vijay and Pal (2023) | India | Randomized controlled trial | YGN: 20, ?F, ?M (11 to 16)  Control: 20, ?F, ?M (11 to 16)  Healthy (adolescents: students) | YGN: Yoga nidra | Duration of session: 25 minutes  Number of sessions per week: 7  Number of weeks: 4 | Delivery setting: In-person  Trained instructor: yes  Steps: i) relaxation of whole body; ii) breathing awareness; iii) visualization | Pre-intervention  Post-intervention | Control: No training | Stress scale (by (Puri et al., 2011))  GAD-7  Aggression scale | **Between-group:** NR  **Within-group**:  Significant ↓ in stress (stress scale) with YGN post-intervention  Significant ↓ in anxiety **(**GAD-7) with YGN post-intervention  Significant ↓ in aggression scale with YGN post-intervention | **RoB2: High risk** |
| Kamble et al. (2023) | India | Quasi experimental design (pre vs post) | 125: ?F, ?M (18 to 25)  Healthy (adults: students) | Yoga nidra + yoga asanas | Duration of session: 30 minutes  Number of weeks: 5  Number of weeks:12  *Yoga prayer, asanas were conducted for warm up for 30 minutes in the beginning | Delivery setting: In-person  Trained instructor: Yes  Steps: i) internalization ii) Sankalpa iii) switching of consciousness or awareness; iv) consciousness of natural breath without changing breath flow; v) recalling, intensifying, experiencing of physical or emotional sensations; vi) visualization; vii) externalization of awareness to become aware of external environment | Pre-intervention  Post-intervention | NA | General anxiety disorder-7  Beck anxiety inventory  Pulse rate  Blood pressure (systolic, diastolic)  Respiratory rate | **Between-group:**  NR  **Within-group:**  Significant ↓ in anxiety (general anxiety disorder-7) with YGN post-intervention  Significant ↓ in anxiety (Beck anxiety inventory) with YGN post-intervention  Significant ↓ in pulse rate with YGN post-intervention  Significant ↓ in blood pressure (systolic and diastolic) with YGN post-intervention  Significant ↓ in respiratory rate with YGN post-intervention | **D&B: Poor** |
| di Fronso et al. (2024) | Italy | Case series | Case 1: 1M (36)  Healthy (adult: sports personnel) | Yoga nidra | Duration of session: 30 minutes  Number of sessions per week: 2  Number of weeks: 4 (1 month)  Total sessions: 10 | Delivery setting: In-person (audio recording)  Trained instructor: Yes  Steps: i) Sankalpa (intention); ii) different body part awareness; iii) breathing awareness; iv) awakening of feelings and emotions; v) visualization exercises; vi) Sankalpa  *Bell sounds used to maintain athletes auditory channel receptive | 5 days before intervention  5 days post-intervention  Follow-up: 3 weeks  Daily assessment of total quality recovery and CR-10 | NA | Perceived stress scale (Italian version)  Recovery-stress questionnaire-36  Total quality recovery  CR-10 scale for assessing rate of perceived exertion  Pre-sleep arousal scale  Pittsburg sleep quality scale  Feeling scale  Multidimensional assessment of interoceptive awareness questionnaire  Actigraphy based sleep assessment (latency, sleep efficiency, total sleep time, wake after sleep onset, number of awakenings, average awakening length)  EEG assessment (individual alpha peak frequency during baseline, stress, recovery phase) | **Between-group:**  NR  **Within-subject:**  Case 1:  Post-intervention:  ↑ in stress (perceived stress scale) with YGN  ↑ in frequency score of sleep quality, being in shape, personal accomplishment, self-efficacy (Recovery-stress questionnaire-36) with YGN  No difference frequency score of general, social stress, social recovery, fatigue, disturbed breaks, emotional exhaustion, injury (Recovery-stress questionnaire-36) with YGN  ↓ frequency score of general well-being (Recovery-stress questionnaire-36) with YGN  Good and very good recovery rated on total quality recovery scale with YGN  ↓ cognitive arousal score on pre-sleep arousal scale with YGN  ↑ somatic arousal score on pre-sleep arousal scale with YGN  ↑ somatic arousal score on pre-sleep arousal scale with YGN  ↑ in Pittsburg sleep quality score with YGN  ↓ Latency, total sleep time, wake after sleep onset, number of awakenings with YGN  ↑ in sleep efficiency, average length of awakening with YGN  Improved feelings noted on feeling scale on YGN  ↓ arousal in terms of energy with YGN  ↓ individualized alpha peak frequency at baseline, stress, recovery with YGN  **Follow-up:**  ↓ in Perceived stress scale with YGN  ↑ in frequency score of sleep quality, being in shape, personal accomplishment, self-efficacy (Recovery-stress questionnaire-36) with YGN  No difference frequency score of general, social stress, social recovery, fatigue, general well-being, disturbed breaks, emotional exhaustion, injury (Recovery-stress questionnaire-36) with YGN  Poor and reasonable recovery rated on total quality recovery scale with YGN  No difference in cognitive arousal score on pre-sleep arousal scale with YGN  ↑ somatic arousal score on pre-sleep arousal scale with YGN  ↑ in Pittsburg sleep quality score with YGN  ↓ sleep efficiency, total sleep time with YGN  ↑ in latency, sleep efficiency, wake after sleep onset, number of awakenings, average length of awakening with YGN  ↓ individualized alpha peak frequency at baseline, stress, recovery with YGN | **D&B: Fair** |
|  |  |  | Case 2: 1F (19)  Healthy (adult: sports personnel) |  |  |  |  |  |  | **Within-subject:**  Case 2:  Post-intervention:  ↓ in stress (perceived stress scale) with YGN  ↑ in frequency score of general, social stress, disturbed breaks (Recovery-stress questionnaire-36) with YGN  No difference in frequency score of sleep quality (Recovery-stress questionnaire-36) with YGN  ↓ in frequency score of fatigue, social recovery, general well-being, emotional exhaustion, injury, being in shape, personal accomplishment, self-efficacy (Recovery-stress questionnaire-36) with YGN  Good and very good recovery rated on total quality recovery scale with YGN  ↓ cognitive arousal score on pre-sleep arousal scale with YGN  ↓ in somatic arousal score on pre-sleep arousal scale with YGN  ↓ in Pittsburg sleep quality score with YGN  ↓ Latency, wake after sleep onset, number of awakenings, average length of awakening with YGN  ↑ in total sleep time, sleep efficiency, with YGN  Improved feelings noted on feeling scale on YGN  ↓ arousal in terms of anxiety with YGN  ↓ individualized alpha peak frequency at baseline, recovery with YGN  ↑ individualized alpha peak frequency at stress with YGN  **Follow-up:**  ↑ in Perceived stress scale with YGN  ↑ in frequency score of general, social stress (Recovery-stress questionnaire-36) with YGN  No difference in frequency score of disturbed breaks, sleep quality (Recovery-stress questionnaire-36) with YGN  ↓ in frequency score of fatigue, social recovery, general well-being, emotional exhaustion, injury, being in shape, personal accomplishment, self-efficacy (Recovery-stress questionnaire-36) with YGN  Great variation in recovery on total quality recovery scale with YGN  ↓ in cognitive arousal score on pre-sleep arousal scale with YGN  No difference in somatic arousal score on pre-sleep arousal scale with YGN  ↓ in Pittsburg sleep quality score with YGN  ↓ in latency, sleep efficiency, average length of awakening with YGN  ↑ in total sleep time, wake after sleep onset, number of awakenings with YGN  ↑ individualized alpha peak frequency at baseline, stress, recovery with YGN |  |
| Rajagopalan et al. (2023) | India | Randomized controlled trial | YGN: 19F, 15M (49.1 ± 8.1)  Control: 17F, 14M (43.9 ± 9.2)  Hypertension | YGN: Yoga nidra + Om chanting  *Om chanting performed for 5 minutes/ 15 rounds | Duration of session: 20 minutes  Number of sessions per week: 5  Number of weeks: 8 | Delivery setting: In-person  Trained instructor: Yes  Steps: NR | Pre-intervention  Mid-intervention (30-day)  Post-intervention | Control: standard care (medications) | DASS  Pittsburg sleep quality index  Heart rate variability parameters (heart rate, mean respiratory rate, high and low frequency power, ratio of low to high frequency, total power, standard deviation of all NN intervals, square root of mean of sum of squares of different adjustment NN intervals, % difference between adjacent NN intervals) | **Between group:**  Significant ↓ in anxiety (subscale of DASS) with YGN as compared to control group  Significant ↓ in depression (subscale of DASS) with YGN as compared to control group  Significant ↓ in stress (subscale of DASS) with YGN as compared to control group  Significant ↓ in overall Pittsburg sleep quality index score with YGN as compared to control group  Significant ↑ in heart rate variability variables (total power, standard deviation of all NN intervals, square root of mean of sum of squares of different adjustment NN intervals, % difference between adjacent NN intervals, high frequency power, mean respiratory rate) with YGN as compared to control group  Significant ↓ in heart rate variability variables (heart rate, low frequency power, ratio of low to high frequency) with YGN as compared to control group  **Within group:**  Significant ↓ in anxiety (subscale of DASS) with YGN post-intervention  Significant ↓ in depression (subscale of DASS) with YGN post-intervention  Significant ↓ in stress subscale of DASS with YGN post-intervention  Significant ↓ in overall Pittsburg sleep quality index score with YGN post-intervention  Significant ↑ in heart rate variability variables (total power, standard deviation of all NN intervals, square root of mean of sum of squares of different adjustment NN intervals, % difference between adjacent NN intervals, high frequency power, mean respiratory rate) with YGN post-intervention  Significant ↓ in heart rate variability variables (heart rate, low frequency power, ratio of low to high frequency) with YGN post-intervention | **RoB2: High risk** |
| Sharpe et al. (2023) | USA | Randomized controlled trial | YGN: 6F, 3M (33 ± 7)  Control: 10F, 1M (30 ± 6)  Mild to moderate insomnia | YGN: Yoga nidra | Duration of session: 30 minutes  Total number of sessions: 1 | Delivery setting: In-person (audio clip)  Trained instructor: Yes  Steps: i) introduction; ii) Antar mouna; iii) Visualize body; iv) Awareness of body and space; v) Awareness of meeting points between body and floor; vi) Resolve; vii) Rotation of consciousness; viii) Natural breath and nadi shodhana instructions; ix) Nadi shodhana independent practice; x) Opposites: heaviness; xi) Opposites: lightness; xii) Opposites: cold; xiii) Opposites: hot; xiv) Opposites: pain; xv) Opposites: pleasure; xvi) Checking focus - "Are you awake?"; xvii) Visualizations; xviii) Resolve; xix) Awareness of body/breath; xx) Omkar chanting; xxi) Mental Omkar chanting; xxii) Awareness of surroundings/room; xxiii) Closing | Pre-intervention  Mid-intervention  Post-intervention  *mid-intervention for respiratory rate, EEG | Control: No training (lying quietly) | STAI  Positive negative affect schedule (positive, negative mood)  Physical, mental, emotional relaxation  Sleep production  Respiratory rate  Polysomnography (sleep onset latency, detection of sleep, sleep staging)  Heart rate variability  EEG α, β, δ, θ power at O1 (occipital 1): indicator of drowsiness and meditative state  Acceptability/tolerability of intervention  Dropout rate | **Between-group:**  No difference in anxiety (STAI) with YGN as compared to control  No difference in positive or negative mood (Positive negative affect schedule) with YGN as compared to control  No difference in positive or negative mood (Positive negative affect schedule) with YGN as compared to control  No difference in physical, mental, emotional relaxation with YGN as compared to control  Significant ↓ in respiratory rate with YGN as compared to control (mid-, and post-intervention)  No difference in detection of sleep, sleep onset latency, sleep staging with YGN as compared to control (mid-, and post-intervention)  No difference in heart rate variability with YGN as compared to control  No difference in EEG α, β, δ, θ power at O1 with YGN as compared to control (mid-, and post-intervention)  **Within-group:**  No difference in anxiety (STAI) with YGN post-intervention  No difference in positive or negative mood (Positive negative affect schedule) with YGN post-intervention  No difference in positive or negative mood (Positive negative affect schedule) with YGN post-intervention  No difference in physical, mental, emotional relaxation with YGN post-intervention  No difference sleep-production with YGN post-intervention  No difference in EEG α, β, δ, θ power at O1 with YGN post-intervention  No difference in detection of sleep with YGN post-intervention  YGN was well-tolerated and ↑ perceived benefit ratings reported  Dropout rate: 5% | **RoB2: Some concern** |
| Navarange et al. (2023) | India | Case study | YGN: 1F (51)  Generalized anxiety disorder | YGN: Yoga nidra + Shirodhara | Duration of session: 30+30 minutes (twice daily)  Number of days per week: 7  Number of days: 60  *Shirodhara performed for 45 minutes for 21 days | Delivery setting: NR  Trained instructor: NR  Steps: NR | Pre-intervention  Mid-intervention (30-day)  Post-intervention | NA | Hamilton anxiety rating scale  Serious adverse events | **Within-subject:**  ↓ in anxiety (Hamilton anxiety rating scale) with YGN post-intervention  No serious adverse events reported | **D&B: Poor** |
| Neha and Kumar (2023) | India | Non-randomized controlled trial | YGN: 50M (18 to 25)  Control: 50M (18 to 25)  Healthy (adults: students) | YGN: Yoga nidra + yoga asanas + Pranayama + Shakarma | Duration of session: 30 minutes  Number of sessions per week: 7  Number of days: 15 | Delivery setting: NR  Trained instructor: NR  Steps: NR | Pre-intervention  Post-intervention | Control: No training | Sinha’s comprehensive anxiety test | **Between-group:**  Significant ↓ in anxiety (Sinha’s comprehensive anxiety test) with YGN as compared to control group  **Within-group:** NR | **D&B: Poor** |
|  |  |  | YGN: 50F (18 to 25)  Control: 50F (18 to 25)  Healthy (adults: students) |  |  |  |  | Control: No training |  | **Between-group:**  Significant ↓ in anxiety (Sinha’s comprehensive anxiety test) with YGN as compared to control group  **Within-group:** NR |  |
| Barik (2023) | India | Quasi experimental design (pre vs post) | 34: ?F, ?M (?)  Healthy (adults*: sports persons)  *age not provided adult category ascertained from the context of recruitement | Yoga nidra | Duration of session: 45 minutes  Number of sessions per week: NR  Number of weeks: NR | Delivery setting: NR  Trained instructor: Yes  Steps: i) breathing in and out; ii) place hands aside with palm facing upwards; iii) continued breathing and feeling rise in belly; iv) inhaling and exhaling deeply; v) relaxing mind and body | Pre-intervention  Post-intervention | NA | Perceived stress scale | **Between-group:** NA  **Within-group:**  Significant ↓ in stress (perceived stress scale) with YGN post-intervention | **D&B: Poor** |
| Gunjiganvi et al. (2023) | India | Randomized controlled trial | YGN: 11F, 29M (32.9 ± 6.4)  Control: 11F, 21M (31.8 ± 6.3)  Healthy (adults: Covid-19 healthcare workers) | YGN: Yoga nidra | YGN:  Duration of session: 30 minutes  Number of days per week: 7  Number of days: 15 days  Control: Duration of session: 30 minutes  Number of days per week: 7  Number of days: 15 days | Delivery setting: At-home (video)  Trained instructor: Yes  Steps: Yoga nidra instructional video demonstration given in supine position with eyes closed for beginners by Swami Niranjanananda Saraswati | Pre-intervention  Post-intervention | Control: Relaxation to deep sleep music in supine position | GAD-7  PHQ-9  Insomnia severity index  Rate of recruitment  Completion of study intervention  Satisfaction concerning accessibility of content through YouTube  Relevance of intervention  Recommendation of intervention to peers | **Between-group:**  Significant ↓ in anxiety (GAD-7) with YGN as compared to control  Significant ↓ in depression (PHQ-9) with YGN as compared to control  Significant ↓ in insomnia severity index with YGN as compared to control  **Within-group:**  Significant ↓ in anxiety (GAD-7) with YGN post-intervention  Significant ↓ in depression (PHQ-9) with YGN post-intervention  Significant ↓ in insomnia severity index with YGN post-intervention  56.8% of contacted participants were recruited  78% of participants completed >9 days of intervention  100% participants expressed satisfaction to accessibility of content  100% participants said they would recommend interventions to their peer groups | **RoB2: High risk** |
| Graham (2022) | India | Quasi experimental design (pre vs post) | 21: ?F, ?M (?)  Healthy (adolescents: students) | Yoga nidra | Duration of session: NR  Number of sessions per week: NR  Number of weeks: NR | Delivery setting: In-person, at-home  Trained instructor: Yes  Steps: i) rotation of consciousness; ii) awareness of prana; iii) awareness of feelings and emotion; iv) visualization; v) final step and return to awareness | Pre-intervention  Post-intervention | NA | Self-made questionnaire (analyzing frequency of students in: high stress, low concentration, bad behavior, low happiness) | **Between-group:** NA  **Within-group:**  ↓ in stress (95.2% i.e., no students classified in high stress group post-intervention) with YGN post-intervention  ↓ in low concentration (95% i.e., no students classified in low concentration group post-intervention) with YGN post-intervention  ↓ in bad behavior changes (100% i.e., no students classified in bad behavior group post-intervention) with YGN post-intervention  ↓ in low happiness (100% i.e., no students classified in low happiness group post-intervention) with YGN post-intervention | **D&B: Poor** |
| Moszeik et al. (2022) | Germany | Randomized controlled trial | YGN: 262F, 64M (19 to 71)  Control: 313F, 81M (19 to 71)  Healthy (adults) | YGN: Yoga nidra | Duration of session: 11 minutes  Number of sessions per week: 7  Number of weeks: 4 | Delivery setting: At-home (audio clip)  Trained instructor: Yes  Steps: perception of individual body part (only left and right side), observation of breath (20 seconds), personal intention, observation of thoughts and feeling integrated | Pre-intervention  Post-intervention  Follow-up: 6 weeks | Control: waitlist control | Screening scale for chronic stress  Positive and negative affect schedule (German version)  Satisfaction with life scale (German version)  Pittsburg sleep quality index  Mindful attention and awareness scale  Adherence of yoga nidra meditation  Frequency of yoga nidra meditation after intervention  Experience with yoga nidra (open-ended questions) | **Between-group (post-intervention and follow-up):**  Substantial ↓ in stress (Screening scale for chronic stress) with YGN as compared to control  Substantial ↑ in positive affect (Positive and negative affect schedule) with YGN as compared to control  Substantial ↓ in negative affect (Positive and negative affect schedule) with YGN as compared to control  Substantial ↑ in satisfaction with life with YGN as compared to control  Substantial ↓ in sleep disturbance (Pittsburg sleep quality index) with YGN as compared to control  Substantial ↑ in mindfulness attention and awareness schedule with life with YGN as compared to control  **Within-group (post-intervention and follow-up):**  ↓ in stress (screening scale for chronic stress) with YGN post-intervention and at follow-up  ↑ positive affect (Positive and negative affect schedule) with YGN post-intervention  ↓ positive affect (Positive and negative affect schedule) with YGN at follow-up  ↓ in negative affect (Positive and negative affect schedule) with YGN post-intervention and at follow-up  ↑ in satisfaction with life with YGN post-intervention and at follow-up  ↓ in sleep disturbance (Pittsburg sleep quality index) with YGN post-intervention and at follow-up  ↑ in mindfulness attention and awareness schedule with life with YGN post-intervention and at follow-up  Open-ended: Relaxation, positive affect, satisfaction: 40% at post-intervention, 42.2% at follow-up  Positive effect on sleep quality: 13.7% at post-intervention, 6.7% at follow-up  Positive body awareness: 11.6% at post-intervention, 6.7% at follow-up  Adherence: 10.5% completed all 30 days, 48.1% at least every second day, 21% did YGN 9 to 15 days, 15.7% did YGN 2 to 8 days  Frequency of YGN after intervention: 51.5% continued YGN, 6.2% did YGN almost every day, 9.3% did YGN several times per week, 16.5% did YGN several times a month, 19.6% once or less than a month, 48.5% did not continue | **RoB2: High risk** |
| Gupta et al. (2022) | India | Quasi experimental design (pre vs post) | YGN: 23F, 7M (29.9 ± 6.7)  Healthy (adults: healthcare workers) | Yoga nidra | Duration of session: 30 minutes  Number of days per week: 7  Number of weeks: 1 | Delivery setting: At-home (audio clip)  Trained instructor: NR  Steps: NR | Pre-intervention  Post-intervention  Follow-up: 14, 21, 28 days | NA | DASS-21  Degree of depression, anxiety, stress relief (i.e., no, moderate, significant relief, negative impact) | **Between-group:** NA  **Within-group:**  Significant ↓ in depression (DASS-21) with YGN post-intervention  Significant ↓ in anxiety (DASS-21) with YGN post-intervention  Significant ↓ in stress (DASS-21) with YGN post-intervention  Degree of depression relief: (no relief: n=9, moderate relief: n=21)  Degree of anxiety relief: (no relief: n=6, moderate relief: 2 n=4)  Degree of stress relief: (no relief: n=2, moderate relief: n=18, significant relief: n=6, negative impact: n=4) | **D&B: Poor** |
| Jaiganesh et al. (2022) | India | Randomized controlled trial | YGN: 15: ?F, ?M (12 to 15)  Control 1: 15: ?F, ?M (12 to 15)  Control 2: 15: ?F, ?M (12 to 15)  Healthy (adolescents: students) | YGN: Yoga nidra + Surya namaskar | Duration of session: 20 to 40 minutes  Number of sessions per week: 5  Number of weeks: 6 | Delivery setting: In person  Trained instructor: NR  Steps: NR | Pre-intervention  Post-intervention | Control 1: Surya namaskar (included poses: Pranamasana, Piraiasana, Padha hastasana, Ashwa Sanchalasana, Sethu bandha asana, Astanga namaskara, Bhujangasana, parvathasana)  Control 2: No training | Everly and Girnando Questionnaire for stress | **Between-group:**  Significant ↓ in stress (Everly and Girnando Questionnaire) with YGN as compared to control 2  No difference in stress (Everly and Girnando Questionnaire) with YGN as compared to control 1  **Within-group:** NR | **RoB2: High risk** |
| Singh et al. (2022) | India | Quasi experimental design (pre vs post) | 14F (26 to 45)  Healthy (adults: during Covid-19) | Virtual iRest yoga nidra + prayer | Duration of session: 13 minutes  Number of days per week: 6  Number of weeks: 6  *One session of virtual YGN conducted before training for familiarization and 1 minute prayer performed before and after the yoga nidra session | Delivery setting: At-home (virtual)  Trained instructor: yes  Steps: opening prayer, body screening, breathing awareness, thought process, feelings and emotions, yogic prayers | Pre-intervention  Post-intervention | NA | DASS-21 | **Between-group:** NA  **Within-group:**  Significant ↓ in depression (DASS-21) with YGN post-intervention  Significant ↓ in anxiety (DASS-21) with YGN post-intervention  Significant ↓ in stress (DASS-21) questionnaire with YGN post-intervention | **D&B: Poor** |
| Kalita and Choudhury (2022) | India | Non-randomized controlled trial | YGN: 8F, 7M (?)  Control: 5F, 10M (?)  Chronic kidney disease undergoing hemodialysis | YGN: Yoga nidra | Duration of session: 10+10 minutes (twice daily)  Number of sessions per weeks: 7  Number of days: 30 | Delivery setting: NR  Delivered by: NR  Steps: NR | Pre-intervention  Post-intervention | Control: No training | DASS-21 | **Between-group:** NR  **Within-group:**  Significant ↓ in anxiety (DASS-21) questionnaire with YGN post-intervention  Significant ↓ in depression (DASS-21) question with YGN post-intervention  Significant ↓ in stress (DASS-21) questionnaire with YGN post-intervention | **D&B: Poor** |
| Kannan and Kumar (2021) | India | Case series | Case 1: 1F (23)  Generalized anxiety disorder | Yoga nidra + Pranayama (diaphragmatic breathing) + Bhramari | Duration of session: NR (twice daily)  Number of sessions per week: 7  Number of weeks: 12 | Delivery setting: in-person, at-home  Trained instructor: NR  Steps: Yoga nidra followed Pranayama sessions, steps NR | Pre-intervention  Post-intervention | NA | Beck anxiety inventory  Depression | **Between-group:** NA  **Within-group:**  ↓ in anxiety with YGN post-intervention  ↓ in depression with YGN post-intervention | **D&B: Poor** |
|  |  |  | Case 2: 1M (26)  Severe social anxiety | Yoga nidra + Pranayama (Bahstrika and Kapalbhati) + counselling  *Dietary changes also included | Duration of session: NR  Number of sessions per week: NR  Number of weeks: 26 |  |  |  | Beck anxiety inventory | **Between-group:** NA  **Within-group:**  ↓ in anxiety with YGN post-intervention |  |
|  |  |  | Case 3: 1M (47)  Irritable bowel syndrome and anxiety | Yoga nidra + Pranayama (Bahstrika and Kapalbhati) + counselling  *Dietary changes also included | Duration of session: NR (twice daily)  Number of sessions per week: NR  Total sessions: 6 |  |  |  | Beck anxiety inventory  Recovery from irritable bowel syndrome | **Between-group:** NA  **Within-group:**  ↓ in anxiety with YGN post-intervention  Recovery from irritable bowel syndrome |  |
| D'Souza et al. (2021) | India | Randomized controlled trial | YGN: 20F, 6M (14 to 16)  Control: 18F, 13M (14 to 16)  Healthy (adolescent) | YGN: Yoga nidra | Duration of session: 30 minutes  Number of days per week: 6  Number of weeks: 3 | Setting: In person  Trained instructor: Yes  Steps: i) postural relaxation; ii) resolve; iii) rotation of consciousness; iv) breath awareness; v) image visualization; vi) resolve; vii) finish | Pre-intervention  Post-intervention | Control: No training | Modified adolescent stress questionnaire | **Between-group:**  Significant ↓ in stress (Modified adolescent stress questionnaire) with YGN as compared to control  **Within-group:**  Significant ↓ in stress (Modified adolescent stress questionnaire) with YGN post-intervention | **RoB2: High risk** |
| Dwivedi (2021) | India | Non-randomized controlled trial | YGN: 33F, 66M (?)  Control: 34F, 66M (?)  Healthy (adults) | YGN: Yoga nidra | Duration of session: NR  Number of sessions per week: NR  Number of weeks: NR | Delivery setting: NR  Delivered by: NR  Steps: NR | Pre-intervention  Post-intervention | Control: No training | Stress scale (40-items on a Likert scale) | **Between-group:**  Significant ↓ in stress (stress questionnaire) with YGN as compared to control  **Within-group:**  Significant ↓ in stress (stress questionnaire) with YGN post-intervention | **D&B: Fair** |
| Sharpe et al. (2021) | USA | Quasi experimental design (pre vs post) | 74: ?F, ?M (?)  Insomnia and anxiety (during Covid-19) | Remotely delivered yoga nidra before bed in real-time or asynchronously | Duration of session: NR  Number of sessions per weeks: 1  Total number of sessions: 16 | Delivery setting: At-home (audio clips)  Trained instructor: NR  Steps: NR | Pre-intervention  Post-intervention | NA | State trait anxiety inventory  Sleep onset latency (sleep survey)  Access of asynchronous recording of YGN  Feasibility (adverse events, post-intervention survey completion rate) | **Between-group:** NA  **Within-group:**  Significant ↓ in anxiety (STAI) With YGN post-intervention  ↓ sleep onset latency with YGN post-intervention  Access of asynchronous recording of YGN: 71/74  No adverse events reported  Completion rate of post-intervention survey: 50% | **D&B: Poor** |
| Sullivan et al. (2021) | USA | Case series | 7: ?F, ?M (?)  Post-traumatic stress disorder (active-duty military personnel) | iRest yoga nidra (Group-classes) | Duration of session: NR  Number of sessions per weeks: NR  Total number of sessions: 18  *Prerecorded sessions for home use provided | Delivery setting: In person (group)  Trained instructor: NR  Steps: NR | Pre-intervention  Pre-intervention  Mid-intervention  Post-intervention | NA | Patient health questionnaire-9  Post-traumatic stress disorder checklist (PCL)  In-person attendance rate  Home-practice adherence rate  Attrition rate | **Between group:** NA  **Within-group:**  General trend in ↓ depression (Patient health questionnaire-9) with YGN post-intervention with three participants demonstrating a change of over 5 points  General trend in ↓ post-traumatic stress (PCL) with YGN post-intervention with three participants demonstrating a change of over 5 points  In-person attendance rate: 67%  Home-practice adherence rate: 42%  Attrition rate: 14% | **D&B: Poor** |
| Kalita (2021) | India | Non-randomized controlled trial | YGN: 5F, 10M (?)  Control: 4F, 11M (?)  Chronic kidney disease undergoing hemodialysis | YGN: Yoga nidra | Duration of session: 10 minutes (daily)  Number of sessions per weeks: 7  Number of days: 15 | Delivery setting: NR  Delivered by: NR  Steps: NR | Pre-intervention  Post-intervention | Control: No training | DASS-21 | **Between-group:** NR  **Within-group:**  Significant ↓ in anxiety (DASS-21) questionnaire with YGN post-intervention  Significant ↓ in depression (DASS-21) question with YGN post-intervention  Significant ↓ in stress (DASS-21) questionnaire with YGN post-intervention | **D&B: Poor** |
| Kaur and Sharma (2021) | India | Non-randomized controlled trial | YGN: 60F (18 to 45)  Control: 60F (18 to 45)  Healthy (adults) | YGN: Yoga nidra | Duration of session: 35 minutes  Number of sessions per weeks: 5  Number of weeks: 12 | Delivery setting: In person  Trained instructor: Yes  Steps: i) prayer; ii) internalization; iii) sankalpa; iv) rotation of consciousness; v) breath awareness; vi) experience of opposite sensations; vii) visualization; viii) sankalpa; ix) externalization | Pre-intervention  Post-intervention | Control: No training | Hamilton anxiety scale  Hamilton rating scale for depression | **Between-group:**  Significant ↓ in anxiety (Hamilton anxiety scale) questionnaire with YGN as compared to control  Significant ↓ in depression (Hamilton rating scale for depression) questionnaire with YGN as compared to control  **Within-group: Between-group:**  Significant ↓ in anxiety (Hamilton anxiety scale) questionnaire with YGN post-intervention  Significant ↓ in depression (Hamilton rating scale for depression) question with YGN post-intervention | **D&B: Fair** |
| D’cunha et al. (2021) | India | Randomized controlled trial | YGN: 24F (35 to 74)  Control: 24F (35 to 74)  Cervical cancer (undergoing radiotherapy) | YGN: Yoga nidra (performed during the course of radiotherapy) | Duration of session: 23 minutes  Number of sessions per week: 5  Number of weeks: 4 | Delivery setting: In-person  Trained instructor: Yes  Step: i) relaxation; ii) resolve; iii) rotation of consciousness; iv) breath awareness | Pre-intervention  Post-intervention | Control: No training | Stress questionnaire (psychological, physical, social, and financial problems) | **Between-group:**  Significant ↓ in stress (Stress questionnaire) questionnaire with YGN as compared to control  **Within-group:**  Significant ↓ in stress (Stress questionnaire) questionnaire with YGN post-intervention | **RoB2: High risk** |
| Joshi (2020) | India | Non-randomized controlled trial | YGN: 28M (16 to 19 years)  Control: 30M (16 to 19 years)  Healthy (adolescents: students) | YGN: Yoga nidra | Duration of session: 30 minutes  Number of sessions per week: 7  Number of weeks: 5  *Procedure repeated initially 5 to 6 times, empty stomach, till participants learn to regulate breathing | Delivery setting: In-person  Trained instructor: Yes  Steps: i) internalization/ relaxation; ii) affirmation (Sankalpa); iii) rotation of consciousness; iv) respiratory awareness; v) manifestation of opposites; vi) creative visualization; vii) affirmation (Sankalpa); viii) return to full awareness | Pre-intervention  Post-intervention | Control: No training | State, trait, and free-floating anxiety scale  Beck depression inventory (Hindi-version) | **Between-group:**  No difference in anxiety (State, trait, and free-floating anxiety scale) between YGN and control group  No difference in depression (BDI) between YGN and control  **Within-group:**  Significant ↓ in anxiety (State, trait, and free-floating anxiety scale) with YGN post-intervention  Significant ↓ in depression (BDI) with YGN post-intervention | **D&B: Poor** |
| De Jesus et al. (2019) | USA | Quasi experimental design (pre vs post) | 16F (64, 50 to 82)  With/at-risk of CVD | YGN: Chair-based yoga nidra | Duration of session: 45 minutes  Number of sessions per week: 1  Number of weeks: 24 | Delivered: In-person (group)  Trained instructor: Yes  Steps: NR | Pre-intervention  Post-intervention | NA | PHQ-9  GAD-7  Perceived stress scale  Daily dietary change patterns  Drop-out rate | **Between-group:** NA  **Within-group:** ↑ in depression (PHQ-9) with YGN post-intervention  ↓ in anxiety (GAD-7) with YGN post-intervention  ↓ in stress (PSS) with YGN post-intervention  Non-statistical reduction in trend reported in how often participants ate foods that were low and/or high in saturated fat with YGN post-intervention  Drop-out rate: 37.5% (10/15 completed) | **D&B: Poor** |
| Wahbeh and Nelson (2019) | USA | Randomized controlled trial | YGN: 12F, 3M (65.7 ± 5.9)  Control: 10F, 4M (64.8 ± 6.7)  Depression (older adults) | YGN: iRest yoga nidra at retreat cener | YGN:  Duration of session (in-person): 14 hours across 2 days (Day 1: 3+4+1 hours, Day 2: 3+3 hours)  Total sessions: 2  At-home:  Duration of session: 20 minutes  Number of days per week: 7  Number of weeks: 6  *Participants in YGN received an iPod with iMINDr application that administered iRest yoga nidra for 6-weeks at home | Delivered: In-person (group), at-home  Trained instructor: Yes  Steps: included i) inner resource; ii) intention; iii) heart felt desire; iv) body sensing/scan; v) breath awareness; vi) awareness for physical sensations; vii) sensing emotions, thoughts, beliefs; viii) witnessing; ix) felt sense of joy; x) integration and actions | Pre-intervention  Post-intervention (in-person, at-home) | Control: No training, hear favorite music and stayed at retreat center | Center for epidemiologic studies depressions scale-5 score  Positive and negative affect schedule-10  Perceived stress scale  Pittsburg sleep quality scale  Pain numerical ration scale (pain intensity, severity)  Brief resilience scale  Spirituality involvement and beliefs scale  Five-factor mindfulness questionnaire  Applied mindfulness process scale  Voice stress analysis (temper, valence, arousal)  Heart rate  Heart rate variability  Credibility and expectancy questionnaire  Enrollment rate  Completion rate  Number of home-practice days (frequency)  Mean practice time at home  Client satisfaction questionnaire | **Between-group:**  **Post-intervention (in-person):**  No difference in positive and negative mood (Positive and negative affect schedule-10) between YGN and control  No difference in pain intensity and severity (Pain numerical ration scale) between YGN and control  No difference in perceived stress (Perceived stress scale) between YGN and control  No difference in resilience (Brief resilience scale) between YGN and control  No difference in applied mindfulness (Applied mindfulness process scale) between YGN and control  No difference in voice stress analysis (temper, valence, arousal) between YGN and control  No difference in heart rate between YGN and control  No difference in heart rate variability between YGN and control  **Post-intervention (home):**  No difference in depression (Center for epidemiologic studies depressions scale-5 score) between YGN and control  No difference in positive and negative mood (Positive and negative affect schedule-10) between YGN and control  No difference in pain intensity and severity (Pain numerical ration scale) between YGN and control  Significant ↓ in sleep disturbance (Pittsburg sleep quality index) with YGN as compared to control  No difference in perceived stress (Perceived stress scale) between YGN and control  No difference in resilience (Brief resilience scale) between YGN and control  No difference in applied mindfulness (Applied mindfulness process scale) and mindfulness (Five-factor mindfulness questionnaire) between YGN and control  No difference in spirituality (Spirituality involvement and belief scale) between YGN and control  Significant ↑ in credibility and expectancy between YGN and control  No difference in the total days practiced at home between YGN and control  No difference in the mean practiced time at home between YGN and control  Enrollment rate: 39%  Completion rate: 97%  Number of home practice days  **Within-group:**  **Post-intervention (in-person):**  No difference in positive and negative mood (Positive and negative affect schedule-10) with YGN post-intervention  No difference in pain intensity and severity (Pain numerical ration scale) with YGN post-intervention  No difference in perceived stress (Perceived stress scale) with YGN post-intervention  No difference in resilience (Brief resilience scale) with YGN post-intervention  No difference in applied mindfulness (Applied mindfulness process scale) with YGN post-intervention  No difference in voice stress analysis (temper, valence, arousal) with YGN post-intervention  No difference in heart rate with YGN post-intervention  No difference in heart rate variability with YGN post-intervention  Positive participant satisfaction (client satisfaction questionnaire) with YGN post-intervention  **Post-intervention (home):**  Significant ↓ in depression (Center for epidemiologic studies depressions scale-5 score) with YGN post-intervention  No difference in positive and negative mood (Positive and negative affect schedule-10) with YGN post-intervention  No difference in pain intensity and severity (Pain numerical ration scale) with YGN post-intervention  ↓ in sleep disturbance (Pittsburg sleep quality index) with YGN post-intervention  No difference in perceived stress (Perceived stress scale) with YGN post-intervention  No difference in resilience (Brief resilience scale) with YGN post-intervention  No difference in applied mindfulness (Applied mindfulness process scale) and mindfulness (Five-factor mindfulness questionnaire) with YGN post-intervention  No difference in spirituality (Spirituality involvement and belief scale) with YGN post-intervention  Positive participant satisfaction (client satisfaction questionnaire)  with YGN post-intervention | **RoB2:** S**ome concern** |
| Dol (2019) | South Korea | Non-randomized controlled trial | YGN: 19F, 1M (22 ± 0.1)  Control: 17F, 3M (21.6 ± 0.2)  Healthy (adults: students) | YGN: Yoga nidra | Duration of session: 60 minutes  Number of sessions per week: 2  Number of weeks: 8 | Delivered: In-person  Trained instructor: Yes  Steps: i) preparing the practice; ii) resolve; iii) rotation of consciousness; iv) awareness of breath; v) feeling and sensation; vi) image visualization; vii) resolve; viii) ending the practice. | Pre-intervention  Post-intervention | Control: No training | Life stress intensity level assessed by visual analog scale  Rosenberg’s self-esteem scale  Side effects | **Between-group:** Significant ↓ in stress (life stress intensity scale) in YGN as compared to control  Significant ↑ in Rosenberg self-esteem scale in YGN as compared to control  **Within-group:**  Significant ↓ in stress (life stress intensity scale) with YGN post-intervention  Significant ↑ in Rosenberg self-esteem scale with YGN post-intervention  Except sleeping (n=20) no side effect reported with YGN post-intervention | **D&B: Fair** |
| Livingston and Collette-Merrill (2018) | USA | Quasi experimental design (pre vs post) | 12F, 3M (44 ± 14)  Healthy (adults: Health care workers) | iRest yoga nidra | Duration of session: 60 minutes  Number of sessions per week: 1  Number of weeks: 8  *Audio recordings with compact discs provided for home training | Delivered: In-person  Trained instructor: Yes  Steps: i) inner resource; ii) intention; iii) heart felt desire; iv) body sensing/scan, v) breath awareness; vi) awareness for physical sensations; vii) sensing emotions; thoughts, beliefs; viii) witnessing; ix) felt sense of joy; x) integration and actions | Pre-intervention  Post-intervention | NA | Department of defense/veteran’s administration pain supplemental questionnaire (activity, mood, sleep, stress)  Five-facet mindfulness questionnaire  Epworth sleepiness scale  Study completion rate  Adherence rate (attended iRest sessions, practices at home) | **Between-group:** NA  **Within-group:**  No difference on stress subscale of the pain supplemental questionnaire with YGN post-intervention  No difference on activity subscale of the pain supplemental questionnaire with YGN post-intervention  No difference on mood subscale of the pain supplemental questionnaire with YGN post-intervention  No difference on sleep subscale of the pain supplemental questionnaire with YGN post-intervention  Significant ↓ in Department of defense/ veterans administration pain supplemental questionnaire score with YGN post-intervention  Significant ↑ in five-facet mindfulness questionnaire with YGN post-intervention  Significant ↓ in Epworth sleepiness scale with YGN post-intervention  Study completion rate: 15/22 (80%)  Adherence: attended iRest sessions (6 ± 1.4)  Practices home training: 7/15 (47%) | **D&B: Poor** |
| Ferreira-Vorkapic et al. (2018) | Brazil | Randomized controlled trial | YGN: 20 ?F, ?M (49.9, SE: 9.2)  Mindfulness 1: 20 ?F, ?M (47.3, SE: 7.5)  Waitlist: 20 ?F, ?M (46.7, SE: 8.1)  Healthy (adults: Professors) | YGN: Yoga nidra | YGN: Duration of session: 45 to 50 minutes  Number of sessions per week: 2  Number of weeks: 13  Mindfulness: (dosage: ?) | Delivered: In-person  Trained instructor: Yes  Steps: YGN developed by Swami Satyananda Saraswati | Pre-intervention  Post-intervention | Mindfulness control: Mindfulness-based stress reduction program prescribed by Kabat-Zinn  Waitlist control: Waitlist control | Beck anxiety inventory  Beck depression inventory  Body sensation questionnaire  Body sensation questionnaire  Hamilton anxiety rating scale (not considered as values in figures were wrongly labelled)  Lipp’s stress symptom inventory for adults  Total general score (feedback questionnaire) | **Between-group:**  Significant ↓ in anxiety (Beck anxiety inventory) with YGN as compared to waitlist control  Significant ↓ in stress (Lipp’s stress syndrome inventory) with YGN as compared to waitlist control  Significant ↓ in body sensation questionnaire with YGN as compared to waitlist control  Significant ↓ in total general score with YGN as compared to waitlist control  No significant difference in depression (Beck depression inventory) as compared to waitlist control  No difference in anxiety (Beck anxiety inventory) with YGN as compared to mindfulness control  No difference in stress (Lipp’s stress syndrome inventory) with YGN as compared to mindfulness control  No difference in body sensation questionnaire with YGN as compared to mindfulness control  No difference in total general score with YGN as compared to mindfulness control  No difference in depression (Beck depression inventory) as compared to mindfulness control  **Within-group:**  Significant ↓ in anxiety (Beck anxiety inventory) with YGN post-intervention  Significant ↓ in Body sensation questionnaire with YGN post-intervention  Significant ↓ in stress (Lipp’s stress symptoms inventory) with YGN post-intervention  No significant difference in depression (Beck depression inventory) with YGN post-intervention | **RoB2: High risk** |
| Vaishnav et al. (2018) | India | Quasi experimental design (pre vs post) | 16F, 20M (13 to 15)  Healthy (adolescents) | Yoga nidra | Duration of session: 30 minutes  Number of sessions per week: 3  Number of weeks: 4 | Delivered: In-person  Trained instructor: Yes  Steps: NR | Pre-intervention  Post-intervention | NA | Visual analog scale (perceived stress)  Cantril’s self-anchoring ladder scale (quality of life)  Faces scale for happiness  Psychological general well-being index-short  Experiential effects of Yoga nidra (enthusiasm, alertness, active, inspired, quietude, clarity of thought, stability, control over anger, self-confidence, and self-awareness)  Qualitative:  Daily diary for observations and experiences  Summary description of experiences through interviews  Narration of reflections in a letter  Feedback from parents and teachers regarding change in participant behavior | **Between-group:** NA  **Within-group:** Significant ↓ in stress (visual analogue scale) with YGN post-intervention  Significant ↑ in quality of life (Cantril’s self-anchoring ladder scale) with YGN post-intervention  Significant ↑ in happiness (i.e., ↓ in Faces scale for happiness) with YGN post-intervention  Significant ↑ in general well-being (i.e., Psychological general well-being index-short) with YGN post-intervention  Significant ↑ in experiential effects of yoga nidra (i.e., enthusiasm, quietude, inspired, alertness, self-confidence, clarity of thoughts, control over anger) with YGN post-intervention  No difference on experiential effects of yoga nidra (i.e., active, self-awareness, stability, self, observation) with YGN post-intervention  Qualitative:  Emotional: all participants reported increased happiness, peace, and vitality  Cognitive: Participants noted improved focus, enhanced enjoyment in learning, and better recall during exams | **D&B: Poor** |
| Tripathi (2018) | India | Non-randomized controlled trial | YGN: 50M (35 to 45)  Control: 50M (35 to 45)  Healthy (adults) | YGN: Yoga nidra | Duration of session: 60 minutes  Number of sessions per week: 5  Number of weeks: 12 | Delivered: In-person  Trained instructor: Yes  Steps: i) preparation; ii) relaxation; iii) resolve; iv) rapid shifting of consciousness; v) awareness of breath; vi) awareness of feelings and sensation; vii) moving visualization of scenarios; viii) finish | Pre-intervention  Post-intervention | Control: No training | Anxiety scale  Self-concept rating scale  Blood pressure (systolic, diastolic)  Heart rate  Vital capacity  Breath holding capacity (positive, negative) | **Between-group:**  Significant ↓ in anxiety (Anxiety scale) with YGN as compared to control  Significant ↑ in self-concept (Self-concept rating scale) with YGN as compared to control  Significant ↓ in blood pressure (systolic, diastolic) with YGN as compared to control  Significant ↑ in vital capacity with YGN as compared to control  Significant ↑ in breath holding capacity (positive, negative) with YGN as compared to control  **Within-group:**  NR | **D&B: Fair** |
| Lakshmipathy and Easvaradoss (2018) | India | Non-randomized controlled trial | YGN: 35F (21.8 ± 0.86)  Control: 35F (21.9 ± 0.83)  Healthy (adults: students) | YGN: Yoga nidra | Duration of session: 90 minutes  Number of sessions per week: 3  Number of weeks: NR  Total sessions: 20 | Delivered: In-person  Trained instructor: Yes  Steps: NR | Pre-intervention  Post-intervention | Control: No training | Perceived stress scale  Self-regulation questionnaire | **Between-group:**  Significant ↓ in stress (perceived stress scale) with YGN as compared to control  No difference in self-regulation (self-regulation questionnaire) with YGN as compared to control  **Within-group:**  Significant ↓ in stress (perceived stress scale) with YGN post-intervention  No difference in self-regulation (self-regulation questionnaire) with YGN post-intervention | **D&B: Poor** |
| Schumann et al. (2018) | Germany | Randomized controlled trial | YGN: 28F, 2M (56.3 ± 8.5)  Control: 24F; 5M (53.5 ± 10.4)  Irritable bowel syndrome | YGN: Yoga nidra + yoga asanas + pranayamas + mantra meditation  *video recordings provided for at-home training | Duration of session: 15 minutes  Number of sessions per week: 1  Number of weeks: 12  *1 session per week was dedicated to yoga nidra and meditation and another session to yoga asanas | Delivery setting: In-person (trainer), at-home (video recordings)  Trained instructor: Yes  Steps: NR | Pre-intervention  Post-intervention  Follow-up: 12 weeks | Control: Nutritional counselling | HADS (anxiety, depression)  Perceived stress questionnaire  Cohen perceived stress scale  Body awareness questionnaire  Body responsiveness scale  Patient global impression of change scale  Irritable bowel syndrome-quality of life scale  Irritable bowel syndrome-symptom severity scale  Short form-36 (Physical, mental component) | **Between-group:**  **Post-intervention:**  Significant ↓ in anxiety (HADS) with YGN as compared to control  No difference in depression (HADS) with YGN as compared to control  No difference in stress (perceived stress questionnaire) with YGN as compared to control  No difference in stress (Cohen’s perceived stress scale) with YGN as compared to control  No difference in body awareness questionnaire with YGN as compared to control  No difference in body responsiveness-1 scale with YGN as compared to control  Significant ↑ in body responsiveness-2 scale with YGN as compared to control  No difference in SF-36 scores (physical, mental) with YGN as compared to control  No difference in irritable bowel syndrome-symptom severity scale with YGN as compared to control  No difference in irritable bowel syndrome-quality of life with YGN as compared to control  **Follow-up:**  No difference in anxiety (HADS) with YGN as compared to control  No difference in depression (HADS) with YGN as compared to control  No difference in stress (perceived stress questionnaire) with YGN as compared to control  No difference in stress (Cohen’s perceived stress scale) with YGN as compared to control  Significant ↑ in body awareness questionnaire with YGN as compared to control  No difference in body responsiveness-1, 2 scale with YGN as compared to control  Significant ↑ in SF-36 scores (physical) with YGN as compared to control  No difference in SF-36 scores (mental) with YGN as compared to control  No difference in irritable bowel syndrome-symptom severity scale with YGN as compared to control  No difference in irritable bowel syndrome-quality of life with YGN as compared to control  **Within-group:**  **Post-intervention:**  Significant ↓ in anxiety (HADS) with YGN post-intervention  Significant ↓ in depression (HADS) with YGN post-intervention  Significant ↓ in stress (perceived stress questionnaire) with YGN post-intervention  Significant ↓ in stress (Cohen’s perceived stress scale) with YGN post-intervention  No difference in body awareness questionnaire with YGN as compared to control  No difference in body responsiveness-1, 2 scale with YGN as compared to control  Significant ↑ in SF-36 scores (physical) with YGN post-intervention  No difference in SF-36 scores (mental) with YGN as compared to control  Significant ↓ in irritable bowel syndrome-symptom severity scale with YGN post-intervention  No difference in irritable bowel syndrome-quality of life with YGN post-interventions  **Follow-up:**  Significant ↓ in anxiety (HADS) with YGN at follow-up  No difference in depression (HADS) with YGN at follow-up  Significant ↓ in stress (perceived stress questionnaire) with YGN at follow-up  Significant ↓ in stress (Cohen’s perceived stress scale) with YGN at follow-up  Significant ↑ in body awareness questionnaire with YGN at follow-up  No difference in body responsiveness-1, 2 scale with YGN at follow-up  Significant ↑ in SF-36 scores (physical) with YGN at follow-up  No difference in SF-36 scores (mental) with YGN at follow-up  Significant ↓ in irritable bowel syndrome-symptom severity scale with YGN at follow-up  No difference in irritable bowel syndrome-quality of life with YGN at follow-up | **RoB2: Some concern** |
| Varma and Khan (2018) | India | Non-randomized controlled trial | YGN: 15: ?F, ?M (28 to 65)  Control: 15: ?F, ?M (28 to 65)  Angina pectoris | YGN: Yoga nidra + pranayama + meditation  Control: No training | Duration of session: NR  Number of sessions: NR  Number of days: 10 | Delivery setting: In-person  Trained instructor: NR  Steps: NR | Pre-intervention  Post-intervention |  | State trait anxiety inventory  Hassles and Uplifts scale for stress | **Between-group:**  Significant ↓ in anxiety (State trait anxiety inventory) questionnaire with YGN as compared to control  Significant ↓ in stress (Hassles and Uplifts scale) questionnaire with YGN as compared to control  **Within-group:**  Significant ↓ in anxiety (State trait anxiety inventory) questionnaire with YGN post-intervention  Significant ↓ in stress (Hassles and Uplifts scale) questionnaire with YGN post-intervention | **D&B: Poor** |
| Anderson et al. (2017) | USA | Quasi experimental design (pre vs post) | 9: ?F, ?M (24 to 49)  Healthy (adults: psychiatric nurses) | Yoga nidra | Duration of session: 90 minutes  Number of weeks: 4  Total number of sessions: 6 | Delivery setting: In-person  Trained instructor: Yes  Steps: NR | Pre-intervention  Post-intervention  Follow-up (4-week) | NA | Stress (Likert scale)  Areas of muscular tension in body (Likert scale)  Sleep patterns (Likert scale) | **Between-group:** NA  **Within-group:**  ↓ in stress (Likert scale) with YGN post-intervention  ↓ in muscle tension (Likert scale) with YGN post each session and post-intervention  Appreciable change in sleep found with YGN post-intervention | **D&B: Poor** |
| Barbuto (2017) | USA | Randomized controlled trial | YGN: 30: ?F, ?M (19 to 56)  Control: 30: ?F, ?M (19 to 56)  Healthy (adults: workers) | YGN: iRest yoga nidra | Duration of session (in-person): 45 minutes + 10 minutes discussion of the 10 steps protocol  Number of sessions per week: 1  Duration of session (at-home): 45 minutes  Number of sessions per week: 5  Number of weeks: 4 | Delivery setting: in-person, at-home  Trained instructor: Yes  Steps: 10 steps beginning with initial relaxation I) setting an intention; ii) heartfelt desire; iii) inner resource; iv) body sensing; v) breath sensing; vi-a) opposite feelings; vi-b) opposite emotion; vii) opposite thoughts/beliefs; viii) joy and well-being; ix) witnessing and pure awareness; x) wholeness and integration | Pre-intervention  Post-intervention | Control: no training | Perceived stress scale  Post-intervention feedback survey  iRest home CD practise diary | **Between-group:**  No difference in stress (Perceived stress scale) between YGN and control group  **Within-group:**  Significant ↓ in stress (Perceived stress scale) with YGN post-intervention  Post-intervention feedback survey:  Participants found iRest to be ‘relatively’ beneficial  70.4% recommended iRest use  45.8% preferred monthly refresher classes  100% reported instructor helped them feel comfortable and addressed questions  iRest home CD practise diary. 20/30 participants used CD  17/30 participants reported iRest CD was helpful and used it 2.2 (0 to 6 days) per week | **RoB2: High risk** |
| Foulkrod et al. (2016) | USA | Case series | 4F (39.5 ± 1.7)  Depression | Yoga nidra + yoga asanas + Pranayamas + processing of thoughts and emotions + talk therapy | Duration of session: 20 minutes  Number of sessions per week: 1  Number of weeks: 6  *70 minutes additional training of yoga asanas, pranayama, processing of thoughts and emotions | Delivery setting: In-person  Trained instructor: Yes  Steps: followed the steps outlined by Satyananda Saraswati, individual steps NR | Pre-intervention  Post-intervention | NA | PHQ-9  GAD-7  Mindfulness attention awareness scale  Self-compassion scale | **Between-group:** NA  **Within-group:**  Significant ↓ in depression (PHQ-9) with YGN post-intervention  No difference in anxiety (GAD-7) with YGN post-intervention  No difference in mindfulness attention awareness scale with YGN post-intervention  Significant ↑ in self-compassion scale with YGN post-intervention | **D&B: Poor** |
| Ferguson (2016) | USA | Quasi experimental design (pre vs post) | 9F, 2M (43.3 ± 11.7)  Healthy (adults: mental health clinicians) | Yoga nidra | Duration of session: 20 minutes  Number of sessions per week: 1  Number of weeks: 6  *Participants provided with audio clip for performing yoga nidra at home | Delivery setting: In-person, at-home  Trained instructor: Yes  Steps: i) preperation; ii) sankalpa; iii) rotation of awareness; iv) breath awareness; v) visualization; vi) repetition of sankalpa and ending practice | Pre-intervention  Post-intervention | NA | Perceived stress scale | **Between-group:** NA  **Within-group:** Significant ↓ in stress (Perceived stress scale) with YGN post-intervention | **D&B: Poor** |
| Genovese and Fondran (2016) | USA | Quasi experimental design (pre vs post) | 50F, 11M (18 to 67)  Healthy (adults: students) | Yoga nidra + yoga asanas + Pranayama | Duration of session: 50 minutes  Number of sessions per week: 2  Number of weeks: 16 | Delivery setting: NR  Trained instructor: Yes  Steps: NR | Pre-intervention  Post-intervention | NA | DASS-21 (stress, anxiety, depression) | **Between-group:** NR  **Within-group:**  Significant ↓ in stress (DASS-21) with YGN post-intervention  Significant ↓ in anxiety (DASS-21) with YGN post-intervention  Significant ↓ in depression (DASS-21) with YGN post-intervention | **D&B: Poor** |
| Manik and Gartia (2016) | India | Non-randomized controlled trial | YGN: 46F, 14M (35.3 ± 11.3)  Control: 44F, 16M (45.3 ± 31.8)  Essential hypertension | YGN: Yoga nidra | YGN: Duration of session: 60 minutes  Number of sessions per week: 7  Number of weeks: 4  Control:  Duration of session: 30 minutes  Number of sessions per week: 7  Number of weeks: 4 | Delivery setting: NR  Trained instructor: NR  Steps: NR | Pre-intervention  Post-intervention | Control: Nadi Shodhana pranayam | Hamilton anxiety rating scale  Blood pressure (systolic, diastolic)  Pulse rate  Breath rate  Body mass index | **Between-group:**  *Analysis unclear (correlation reported between outcomes)  **Within-group:**  Significant ↓ in anxiety (Hamilton anxiety ratings cale) with YGN post-intervention  Significant ↓ in blood pressure (systolic, diastolic) with YGN post-intervention  Significant ↓ in pulse rate with YGN post-intervention  Significant ↓ in breath rate with YGN post-intervention  Significant ↓ in body mass index with YGN post-intervention | **D&B: Fair** |
| Singh and Adhikari (2016) | India | Quasi experimental design (pre vs post) | 60 M (?)  Healthy (adults: army personnel-shooters) | Yoga nidra + prayer + asanas + pragya yogasanas + shavasana + pranayama | Duration of session: 35 minutes  Number of sessions per week: 7  Number of weeks: 4  *145 minutes of additional yoga asanas and prayer | Delivery setting: In-person  Trained instructor: Yes  Steps: NR | Pre-intervention  Post-intervention | NA | Occupational stress index (low, moderate, high classification) | **Between-group:** NA  **Within-group:**  Significant ↓ in number of participants classified in the high occupational stress category from 20% to 10% with YGN post-intervention  Significant ↓ in number of participants classified in the moderate occupational stress category from 46.7% to 40% with YGN post-intervention  Significant ↑ in number of participants classified in the low occupational stress category from 33.3% to 50% with YGN post-intervention | **D&B: Poor** |
| Chaudhary and Pal (2016) | India | Quasi experimental design (pre vs post) | 20 ?F, ?M (?)  Spondylitis and backache | Yoga nidra | Duration of session: 30 minutes  Number of sessions per week: 7  Number of days: 45 | Delivery setting: NR  Trained instructor: NR  Steps: NR | Pre-intervention  Post-intervention | NA | Stress scale by M. Singh (reference not available) | **Between-group:** NA  **Within-group:**  Significant ↓ in stress (Stress scale) with YGN post-intervention | **D&B: Poor** |
| Rani et al. (2016) | India | Randomized controlled trial | YGN: 45F (26.5 ± 7.4)  Control healthy: 42F (25.5 ± 6.2)  Menstrual disorders | YGN: Yoga nidra + medications | YGN:  Duration of session: 30 to 35 minutes  Number of sessions per week: 5  Number of weeks: 24  *In-person training: 3 months  Home training: 3 months  Control: - | Delivery setting: In-person, at-home  Trained instructor: Yes  Steps: Deep relaxation technique guided by a yoga instructor. YGN steps according to Swami Satyananda Saraswati, individual steps NR | Pre-intervention  Post-intervention | Control: medications | Psychological general well-being tool evaluating anxiety, depression, positive wellbeing, self-control, general healthy, vitality  Adherence to intervention  Laboratory assessment (thyroid stimulating hormone, follicle stimulating hormone, luteinizing hormone, prolactin, progesterone, testosterone. estradiol, dehyroepiandrostone sulfate) | **Between-group:**  Significant ↓ in anxiety (Psychological general well-being tool) in YGN as compared to control  Significant ↓ in depression (Psychological general well-being tool) in YGN as compared to control  Significant ↑ in positive wellbeing (Psychological general well-being tool) in YGN as compared to control  No difference in self-control (Psychological general well-being tool) with YGN as compared to control  Significant ↑ in general health (Psychological general well-being tool) in YGN as compared to control  Significant ↑ in vitality (Psychological general well-being tool) in YGN as compared to control  Significant ↓ in thyroid stimulating hormone, follicle stimulating hormone, luteinizing hormone, prolactin in YGN as compared to control  No difference in progesterone, testosterone. estradiol, dehyroepiandrostone sulfate with YGN as compared to control  **Within-group:**  Significant ↓ in anxiety (Psychological general well-being tool) in YGN post-intervention  Significant ↓ in depression (Psychological general well-being tool) in YGN post-intervention  Significant ↑ in positive wellbeing (Psychological general well-being tool) in YGN post-intervention  No difference in self-control (Psychological general well-being tool) with YGN post-intervention  Significant ↑ in general health (Psychological general well-being tool) in YGN post-intervention  Significant ↑ in vitality (Psychological general well-being tool) in YGN post-intervention  Significant ↓ in thyroid stimulating hormone, follicle stimulating hormone, luteinizing hormone, prolactin in YGN post-intervention  No difference in progesterone, testosterone. estradiol, dehyroepiandrostone sulfate with YGN post-intervention  45/50 recruited participants completed 80% of yoga nidra sessions | **RoB2: High risk** |
| Yadav and Sardar (2016) | India | Randomized controlled trial | YGN: 50 (?F, ?M) (18 to 24)  Control: 50 (?F, ?M) (18 to 24)  Healthy (adults: sports persons) | YGN: Yoga nidra | YGN: Duration of session: 35 minutes  Number of sessions per week: 7  Number of weeks: 2  Control: Duration of session: 35 minutes  Number of sessions per week: 7  Number of weeks: 2 | Delivery setting: In-person  Trained instructor: NR  Steps: YGN steps outlined by Swami Satyananda Saraswati, individual steps NR | Pre-intervention  Post-intervention | Control: practice of yoga asanas (i.e., postures) including Surya namskar, Paschimotanasana, Dhanurasana, Bhujangasana, Ardhchakrasana, Tadasana | EdFenn Beck Anxiety Self Rating Scale (Beck anxiety inventory) | **Between-group:**  Significant ↓ in anxiety (Beck Anxiety inventory) in YGN as compared to control  **Within-group:**  Significant ↓ in anxiety (Beck anxiety inventory) with YGN post-intervention | **RoB2: High risk** |
| Pence et al. (2014) | USA | Quasi experimental design (pre vs post) | 15F (56, 32 to 82)  Sexual trauma | iRest yoga nidra | Duration of session: 90 minutes  Number of sessions per week: 2  Number of weeks: 10  *Total sessions: 19 | Delivery setting: in-person  Trained instructor: Yes  Steps: i) inner resource; ii) intention; iii) heart felt desire; iv) body sensing/scan; v) breath awareness; vi) awareness for physical sensations; vii) sensing emotions, thoughts, beliefs; viii) witnessing; ix) felt sense of joy; x) integration and actions | Pre-intervention  Post-intervention | NA | Brief symptom inventory (depression, anxiety, somatization)  Posttraumatic cognitions inventory  Posttraumatic stress disorder checklist  Toleration of iRest  Attrition rate  Qualitative (informal conversations)  Adverse event | **Between-group:** NA  **Within-group:**  Significant ↓ in depression (Brief symptom inventory) with YGN post-intervention  Significant ↓ in overall posttraumatic stress disorder checklist with YGN post-intervention  No difference in anxiety (Brief symptom inventory) with YGN post-intervention  No difference in somatization (Brief symptom inventory) with YGN post-intervention  No difference in posttraumatic cognitions inventory with YGN post-intervention  Toleration: iRest well tolerated with no adverse responses recorded  Attrition: 5/15 participants dropped out  Qualitative: Participants reported increased sense of well-being, joy, ability to be intrusive about thoughts, emotions, memories, improved quality of sleep, improved ease of breathing, improved ability to relax, overall reduction in physical tension, and improved ability to manage life’s stressors  No adverse events reported | **D&B: Fair** |
| Rani et al. (2013) | India | Quasi experimental design (pre vs post) | 42F, 8M (17 to 25)  Healthy (adults: nursing students) | Yoga nidra | Duration of session: 48 minutes  Number of sessions per week: 6  Number of days: 20 | Delivery setting: In person (audio-recording)  Trained instructor: Yes  Steps: i) preparation; ii) sankalpa; iii) rotation of consciousness; iv) breath awareness; v) opposite feelings and sensations; vi) visualization; vii) sankalpa; viiii) ending the practices | Pre-intervention  Post-intervention | NA | Modified stress assessment scale by M. Singh  Comparison of stress level among students (severe, high, moderate, low, very low) | **Between-group:** NA  **Within-group:**  Significant ↓ in stress (perceived stress scale) with YGN post-intervention  Stress level: Number of participants with very low stress increased from 46% to 82% with YGN post-intervention | **D&B: Poor** |
| Chowdhary (2013) | India | Non-randomized controlled trial | YGN: 30 ?F, ?M (17 to 22)  Control 1: 30 ?F, ?M (17 to 22)  Control 2: 30 ?F, ?M (17 to 22)  Healthy (adults: students, tribal) | YGN: Yoga nidra | Duration of session: 20 to 40 minutes  Number of sessions per week: 5  Number of weeks: 12 | Delivery setting: In-person  Trained instructor: NR  Steps: NR | Pre-intervention  Post-intervention | Control 1: Pranayamas (Kapalbhayi, Bhramari, Nadi Sodhan)  Control 2: No training | Stress and social adjustment scale | **Between-group:**  Significant ↓ in stress (Stress and social adjustment scale) with YGN as compared to control 2  No difference in stress (Stress and social adjustment scale) with YGN as compared to control 1  Significant ↑ in social adjustment (Stress and social adjustment scale) with YGN as compared to control 2  No difference in social adjustment (Stress and social adjustment scale) with YGN as compared to control 1  **Within-group:** NR | **D&B: Poor** |
| Eastman-Mueller et al. (2013) | USA | Quasi experimental design (pre vs post) | 50F, 16M (18 to 56)  Healthy (adults: students) | iRest yoga nidra | Duration of session: 45 minutes  Number of sessions per week: 1  Number of weeks: 8 | Delivery setting: In-person, at-home  Trained instructor: Yes  Steps: i) orientation regarding overview, history, and significant of iRest yoga nidra; ii) y review of posture; iii) identification of inner resource, heartfelt desire, and intention; iv) body sensing and breath awareness exercises; v) working with conflicting emotions or beliefs; vi) experiencing inner strength and joy  *Auditory recordings of practice provided and journalling of experiences was recommended | Quantitative: Pre-intervention  Post-intervention  Qualitative (week 4 and week 8) | NA | Perceived stress scale  BDI  Penn state worry questionnaire  Five facet mindfulness questionnaire (non-react, actaware, observe, describe, nonjudge)  Qualitative perceptions of participants | **Between-group:** NA  **Within-group:**  Significant ↓ in stress (perceived stress scale) with YGN post-intervention  Significant ↓ in depression (BDI) with YGN post-intervention  Significant ↓ in Penn state worry questionnaire with YGN post-intervention  Significant ↑ in five facet mindfulness questionnaires (all subscales: non-react, actaware, observe, describe, nonjudge) in YGN post-intervention  Participants felt relaxed, calmer, better able to sleep, increased body awareness, increased ability to gain perspective and manage emotions, and increased feelings of acceptance | **D&B: Poor** |
| Jensen et al. (2012) | Australia | Case series | 7M (12.6 ± 2)  Behavioral dysfunction | Yoga nidra + asanas + breathing exercises | Duration of session: 20 minutes  Number of sessions per week: 2 to 3  Number of weeks: 13  Total sessions: 17.3 ± 1.9 | Delivery setting: In person (group)  Trained instructor: Yes  Steps: i) correct body positioning; ii) awareness of environmental sounds; iii) affirmation; iv) breath awareness; v) systematic awareness of body parts; vi) listening to sounds, re-orientation to external awareness | Pre-intervention  Mid-intervention  Post-intervention | NA | STAI  STAI-children  Self-description questionnaire 1 and 2  Conner’s teachers rating scale revised-long version (anxious/shy, hyperactive, inattentive, emotional ability)  Respiratory pattern | **Between-group:** NA  **Within-group:**  No difference in anxiety (STAI) with YGN post-intervention  No difference in anxiety (STAI-children) with YGN post-intervention  No difference in self-description questionnaire 1 and 2 with YGN post-intervention  No difference in Conner’s teachers rating scale revised-long version (anxious/shy, hyperactive, inattentive, emotional ability) with YGN post-intervention  Majority of participants (n=6) showed a stable breathing pattern with YGN post-intervention | **D&B: Poor** |
| Lukács et al. (2012) | Hungary | Non-randomized controlled trial | YGN: 48F, 19M (20.5 ± 0.7)  Control: 51F, 24M (20.0 ± 0.5)  Healthy (adults: students) | YGN: Yoga nidra  Control: No training | Duration of session: 20 to 25 minutes  Number of sessions per week: 1  Number of weeks: 14  *Also asked practice at home | Delivery setting: In person, at-home  Trained instructor: Yes  Steps: performed in prone position, individual steps not reported | Pre-intervention  Post-intervention |  | BDI  Qualitative experience with yoga nidra | **Between-group:**  Significant ↓ in depression (BDI) with YGN as compared to control  **Within-group:**  Significant ↓ in depression (BDI) with YGN post-intervention  Qualitative:  Short-term: Students reported being calm, relaxed, felt more energetic, more balanced with YGN  Long-term effect: Positive effect reported on sleep, self-confidence, courage, mental performance (i.e., efficient learning, concentration) with YGN  Participants (n=3) reported becoming sleepy and languorous after deep relaxation |  |
| Rani et al. (2012) | India | Randomized controlled trial | YGN: 56F (27.6 ± 7.8)  Control: 54F (26.5 ± 6.8)  Menstrual disorders | YGN: Yoga nidra + medications | YGN: Duration of session: 35 minutes  Number of sessions per week: 5  Number of weeks: 26  Control: - | Delivery setting: in-person  Trained instructor: Yes  Steps: YGN developed by Swami Satyananda Saraswati with deep relaxation technique | Pre-intervention  Post-intervention | Control: medications | Hamilton anxiety rating scale for anxiety  Hamilton rating scale for depression  Drop out | **Between-group:**  Significant ↓ in anxiety (Hamilton anxiety rating scale for anxiety) in YGN as compared to control  Significant ↓ in depression (Hamilton rating scale for depression) with YGN as compared to control  **Within-group:**  Significant ↓ in anxiety (Hamilton anxiety rating scale for anxiety) with YGN post-intervention  Significant ↓ in depression (Hamilton rating scale for depression) with YGN post-intervention  Participants (n=10) dropped out in YGN group  Participants (n=14) dropped out in control group | **RoB2: High risk** |
| Bhogaonker (2012) | USA | Non-randomized controlled trial | YGN: 117: ?F, ?M (18 to 66)  Control: 79: ?F, ?M (18 to 66)  Homeless adults | YGN: iRest yoga nidra | Duration of session: 30 to 45 minutes  Number of sessions per week: 1  Number of weeks: 4 | Delivery setting: in-person (group)  Trained instructor: Yes  Steps: i) development of intention; ii) body scan; awareness of breath and energy; iii) systematic neutralization of negative physical and emotional cues, beliefs, and memories; iv) embodied experience of joy and wellness; v) freedom from sense of separateness or ego | Pre-intervention  Post-intervention | Control: treatment as usual received housing and services only | Perceived stress scale  Positive and negative affect schedule  Kellner symptom questionnaire (anxiety, depression, hostility-irritability, somatization and four sub-scales of well-being)  Quality of life satisfaction scale  Dropout rate | **Between-group:**  No between-group differences reported in pre-test values between YGN and control group  **Within-group:**  Significant ↓ in stress (perceived stress scale) with YGN post-intervention  Significant ↓ in negative affect (Positive and negative affect schedule) with YGN post-intervention  Significant ↑ in quality of life (quality of life satisfaction scale) with YGN post-intervention  No effect on positive affect (Positive and negative affect schedule) with YGN post-intervention  Significant ↓ in anxiety (Kellner symptom questionnaire) with YGN post-intervention  Significant ↓ in depression (Kellner symptom questionnaire) with YGN post-intervention  Significant ↓ in somatic symptoms (Kellner symptom questionnaire) with YGN post-intervention  Significant ↓ in hostility (Kellner symptom questionnaire) with YGN post-intervention  Drop out:  YGN: 51/117  Control: 27/79 | **D&B: Poor** |
| Anuja (2011) | India | Quasi experimental design (pre vs post) | 30F (20 to 25 years)  Healthy (adults: students) | Yoga nidra | Duration of session: 30 minutes  Number of sessions per week: 7  Number of weeks: 4 | Delivery setting: in-person  Trained instructor: NR  Steps: i) preparation; ii) relaxation; iii) resolve; iv) rotation of consciousness; v) breathing; vi) image visualization; vii) repetition of resolve; viii) finish | Pre-intervention  Post-intervention | NA | Sinha’s comprehensive anxiety test | Between-group:  NA  **Within-group:**  Significant ↓ in anxiety (Sinha’s comprehensive anxiety test) with YGN post-intervention | **D&B: Poor** |
| Stankovic (2011) | USA | Qualitative case series | 16M (41 to 66)  Combat-related post-traumatic stress disorder (combat veterans) | iRest yoga nidra  *also receiving concurrent individual or group counselling | Duration of session: 40 minutes  Number of sessions per week: 1  Number of weeks: 8  *iRest was part of a 120 minutes session where questionnaires were filled  **Participants were provided with audio recordings through CD for at-home practice | Delivery setting: in-person (group), at-home  Trained instructor: Yes  Steps: Beginner practitioners: i) detailed body scan; ii) breath awareness and/or breath counting; iii) focus on simple sensations such as warmth, heaviness, pleasure  Advanced practitioners: i) sense impact of specific emotions, images, or memories in the body; ii) investigate subtle experiences such as self-sense or “I-thought” | Pre-intervention  Post-intervention  Follow-up: 1 year | NA | Qualitative: self-rating data about post-traumatic stress disorder symptoms  Responses to and challenges with iRest yoga nidra  Feedback on class series (protocol, at-home training, symptom changes attributed to iRest yoga nidra)  Permanency of changes in symptoms (1 to 5)  Participant attendance  Frequency of home practices | **Between-group:**  NR  **Within-group:**  **Symptom change:**  Participants reported minor ↓ in mental stress, physical pain, anxiety, anger, emotional volatility, blood pressure, self-judgement with YGN  Participants reported ↑ ability to relax, feel positive emotions, spiritually connected with YGN  **Participant experience:**  Participants reported positive experiences, valued group YGN sessions and reported greater sense of safety and opportunity to learn from one another  (N= 9) participants liked at-home YGN sessions, however preferred the duration to be 30 minutes instead of 40 minutes  Participants developed conditioned relaxation responses to CD recordings and played in background while carrying out ADLs  Participants preferred CD recordings that emphasized on sensation of body and breath and included simplest guided movements of attention  Participants initially had problems distinguishing in their bodies between simple feelings and complex sensations of emotions but gradually felt sensations more readily (reported increased awareness of energy flows, sensations of tension, pleasure, relaxation, changes in physical mobility) with YGN training  At 1-year follow up: (n=5) participants continued at-home YGN, weekly, once a month  Extent of changes:  Helpful: (n=4)  Moderately helpful: (n= 4)  Little bit helpful: (n=3)  Did not help at all: (n= 0)  Part of iRest YGN that was most helpful:  Body sensing: 74%  Awareness: 60%  Bliss, inner strength: 57%  Beliefs: 26% (least)  Average helpfulness rating for inner resources: 46%  Permanency of changes: 3.27 or semi-permanent  Participants reported YGN was helpful in physical and emotional improvements and stronger feelings of self-efficacy and greater spiritual peace  Challenges:  In-person session: distractions, loosing focus and falling asleep (while working with beliefs), found it difficult to stay mindful long enough to unfold and resolve, working with experiential opposites, following all instructions within 40 minutes session  Drop-out: 31.2%  Participant attendance: 93%  At-home practice: 2.36 times per week | **MMAT: High quality** |
| Rani et al. (2011) | India | Randomized controlled trial | YGN: 75F (28.5 ± 7.0)  Control healthy: 75F (27.6 ± 7.7)  Menstrual disorders | YGN: Yoga nidra + medications | YGN:  Duration of session: 35 minutes  Number of sessions per week: 5  Number of weeks: 26  Control: - | Deliver setting: In-person  Trained instructor: Yes  Steps: Deep relaxation technique guided including i) preparation; ii) relaxation; iii) resolve; iv) rotation of consciousness; v) breathing; vi) breathing; vii) image visualization; viii) resolve; ix) finish. YGN developed by Swami Satyananda Saraswati | Pre-intervention  Post-intervention | Control: medications | Psychological general well-being tool (evaluating: anxiety, depression, positive wellbeing, self-control, general healthy, vitality)  Adherence to intervention | **Between-group:** Significant ↓ in anxiety (Psychological general well-being tool) in YGN as compared to control  Significant ↓ in depression (Psychological general well-being tool) in YGN as compared to control  Significant ↑ in positive wellbeing (Psychological general well-being tool) in YGN as compared to control  Significant ↑ in self-control (Psychological general well-being tool) in YGN as compared to control  Significant ↑ in general health (Psychological general well-being tool) in YGN as compared to control  Significant ↑ in vitality (Psychological general well-being tool) in YGN as compared to control  65/75 recruited participants completed 80% of yoga nidra sessions  **Within-group:** NR | **RoB2: High risk** |
| Birdsall et al. (2011) | USA | Quasi experimental design (pre vs post) | 22 (?F, ?M)  (?)  Healthy (adults: school counsellors) | iRest yoga nidra | Duration of session: 90 minutes  Number of sessions per week: 1  Number of weeks: 6  Home: 1 session/day  *Audio recordings with compact discs provided for home training | Delivery setting: In person, at-home  Trained instructor: NR  Steps: included body scan meditation, breath work, exploration of sensations, emotions and thought patterns, moving back and forth between feelings and witnessing, and sitting in awareness | Pre-intervention  Post-intervention | NA | Perceived stress scale  Profile of moods scale (depression, tension, anger, vigor, confusion, fatigue) | **Between-group:** NA  **Within group:**  Significant ↓ in stress (perceived stress scale) with YGN post intervention  Significant ↓ in fatigue (profile of moods scale) with YGN post intervention  No difference in depression (profile of moods scale) with YGN post intervention  No difference in tension (profile of moods scale) with YGN post intervention  No difference in anger (profile of moods scale) with YGN post intervention  No difference in vigor (profile of moods scale) with YGN post intervention  No difference in confusion (profile of moods scale) with YGN post intervention  No difference in overall profile of moods scale with YGN post intervention | **D&B: Poor** |
| Pritchard et al. (2010) | USA | Quasi experimental design (pre vs post) | 7 (?F, ?M) (?)  Cancer | iRest yoga nidra | Duration of session: 90 minutes  Number of sessions per week: 1  Number of weeks: 6  Home: 1 session/day  *Audio recordings with compact discs provided for home training | Delivery setting: In person, at-home  Trained instructor: NR  Steps: included body scan meditation, breath work, exploration of sensations, emotions and thought patterns, moving back and forth between feelings and witnessing, and sitting in awareness | Pre-intervention  Post-intervention | NA | Perceived stress scale | **Between-group:** NA  **Within-group:**  Significant ↓ in stress (perceived stress scale) with YGN post intervention in individuals with cancer | **D&B: Poor** |
|  |  |  | 9 (?F, ?M) (?)  Multiple sclerosis |  |  |  |  |  |  | **Between-group:** NA  **Within-group:**  Significant ↓ in stress (perceived stress scale) with YGN post intervention in individuals with multiple sclerosis |  |
| Deuskar (2010) | India | Randomized controlled trial | YGN: 42F (30 to 45)  Control healthy: 53F (30 to 45)  Healthy (mothers) | YGN: Yoga nidra | YGN: 30 minutes session  Number of days: 45 days  Total: 30 sessions  Control: - | Delivery setting: In person  Trained instructor: NR  Steps: Audio recording of Yoga nidra including instructions for: resolve making, deep muscle relaxation, observation of breath, evoking sensations of warmth and cold, pleasure and pain, heaviness and lightness in quick successions, creative visualization, recalling the resolve and gradual awakening | Pre-intervention  Post-intervention | Control: no training | Smith stress symptoms inventory (worry and negative emotion, autonomic arousal and anxiety, attention deficit, depression, striated muscle tension, interpersonal conflict and anger)  Blood pressure (systolic, diastolic)  Hemoglobin  Variance in blood glucose scores | **Between-group:**  Significant ↓ in subscale variables of Smith stress inventory (autonomic arousal and anxiety, attention deficit, depression, striated muscle tension) in YGN as compared to control  No difference on subscale variables of Smith stress inventory (worry and negative emotion, interpersonal conflict and anger) between YGN and control  Significant ↓ in blood pressure (systolic, diastolic) in YGN as compared to control  **Within-group**: NR  Significant ↑ in hemoglobin in YGN as compared to control  Significant ↓ in variance in blood glucose scores in YGN as compared to control | **RoB2: High risk** |
| Kumar (2008) | India | Non-randomized controlled trial | YGN: 40F, 40M (20 to 30 years)  Control: 15F, 15M (20 to 30 years)  Healthy (adults: students) | YGN: Yoga nidra + practice of asanas, pranayamas, shatkarmas | YGN:  Duration of session: 30 minutes  Number of sessions per week: 6  Number of weeks: 24  Control: ? | Delivery setting: In person  Trained instructor: Yes  Steps: involved flat lying in shavasana, included: resolve, body part awareness, breath awareness, and visualization. YGN developed by Swami Satyananda Saraswati | Post-intervention | Control: practice of asanas (i.e., postures), pranayamas, shatkarmas | Eight-state questionnaire (anxiety and stress) | **Between-group:**  Significant ↓ in anxiety (Eight-state questionnaire) with YGN as compared to control  Significant ↓ in stress (Eight-state questionnaire) with YGN as compared to control  **Within-group:** NR | **D&B: Poor** |
| K. (2008) | India | Randomized controlled trial | YGN 1: 10F, 10M (17 to 25)  YGN 2: 10F, 10M (17 to 25)  Control 1: 10F, 10M (17 to 25)  Control 2: 10F, 10M (17 to 25)  Healthy (adults: students) | YGN 1: Yoga nidra  YGN 2: Yoga nidra + cyclic meditation (alternatively)  *Cyclic meditation (Relaxation techniques: instant, quick, deep + seven yoga asanas) | YGN1, YGN 2: Duration of session: 45 minutes  Number of sessions per week: 3  Number of weeks: 12  *same training duration for cyclic meditation | Delivery setting: In person  Trained instructor: Yes  Steps: Delivered in supine position, i) preparation; ii) relaxation; iii) resolve; iv) rotation of consciousness; v) awareness of breath, feeling and sensations; vi) image visualization; vii) resolve; vii) finish | Pre-intervention  Post-intervention | Control 1: Cyclic meditation  Control 2: no training  *Cyclic meditation (Relaxation techniques: instant, quick, deep + seven yoga asanas) | Competition State Anxiety Inventory-2 questionnaire (cognitive, somatic)  Inventory of sports aggression  Self-confidence scale  Body temperature  Blood pressure (systolic, diastolic)  Respiratory rate  Pulse rate | **Between-group:**  Significant ↓ in cognitive, somatic anxiety (Competition State Anxiety Inventory) with YGN 1 and YGN 2 as compared to control 2  No difference in cognitive, somatic anxiety (Competition State Anxiety Inventory) with YGN 1 and as compared to control 1  Significant ↑ in self-confidence with YGN 1 and YGN 2 as compared to control 2  No difference in self-confidence with YGN 1 and as compared to control 1  No difference in instrumental aggression with YGN 1, YGN 2 and as compared to control 1 and control 2  Significant ↓ in hostile aggression with YGN 1 as compared to control 2  No difference in hostile aggression between YGN 1 and control 1  No difference in hostile aggression between YGN 2 and control 1 and 2  Significant ↓ in body temperature with YGN 1 and YGN 2 as compared to control 2  No difference in body temperature with YGN 1 and as compared to control 1  Significant ↓ blood pressure (systolic, diastolic) with YGN 1 and YGN 2 as compared to control 2  No difference in blood pressure (systolic, diastolic) with YGN 1 and as compared to control 1  Significant ↓ respiratory rate with YGN 1 and YGN 2 as compared to control 2  Significant ↓ respiratory rate with YGN 2 as compared to control 1  No difference in respiratory rate with YGN 1 as compared to control 2  Significant ↓ pulse rate with YGN 1 and YGN 2 as compared to control 2  Significant ↓ pulse rate with YGN 2 as compared to control 1  No difference in pulse rate with YGN 1 as compared to control 2  **Within-group:**  Significant ↓ in cognitive, somatic anxiety (Competition State Anxiety Inventory) with YGN 1 and YGN 2 post-intervention  Significant ↓ body temperature with YGN 1 and YGN 2 post-intervention  Significant ↑ self-confidence with YGN 1 and YGN 2 post-intervention  Significant ↑ in instrumental aggression with YGN 1 and YGN 2 post-intervention  Significant ↓ in hostile aggression with YGN 1 and YGN 2 post-intervention  Significant ↓ blood pressure (systolic, diastolic) with YGN 1 and YGN 2 post-intervention  Significant ↓ respiratory rate with YGN 1 and YGN 2 post-intervention  Significant ↓ pulse rate with YGN 1 and YGN 2 post-intervention | **RoB2: High risk** |
| Kumar (2004) | India | Non-randomized controlled trial | YGN: 20F, 20M (20 to 30 years)  Control: 6F, 6M (20 to 30 years)  Healthy (adults: students) | YGN: Yoga nidra + practice of asanas, pranayamas, shatkarmas | YGN:  Duration of session: 30 minutes  Number of sessions per week: 6  Number of weeks: 24  Control: ? | Delivery setting: In-person  Trained instructor: Yes  Steps: Practice of yoga nidra involved flat lying in shavasana, steps included: resolve, body part awareness, breath awareness, and visualization. YGN developed by Swami Satyananda Saraswati | Post-intervention | Control: practice of asanas (i.e., postures), pranayamas, shatkarmas | Eight-state questionnaire (anxiety and stress)  PGI general well-being measure | **Between-group:**  Significant ↓ in anxiety (Eight-state questionnaire) with YGN as compared to control  Significant ↓ in stress (Eight-state questionnaire) with YGN as compared to control  Significant ↑ in general well-being (PGI general well-being measure) with YGN as compared to control  **Within-group:** NR | **D&B: Poor** |
| Bhushan and Sinha (2001) | India | Quasi experimental design (pre vs post) | 7F, 20M (19 to 50)  Healthy (adults: students) | Yoga nidra | Duration of session: 60 minutes  Number of sessions per week: 7  Number of days: 15 | Delivery setting: In-person  Trained instructor: Yes  Steps: i) preparation; ii) samkalpa; iii) Rotation of consciousness; iv) breath awareness; v) creating opposite feelings; vi) visualisation; vii) viii) repetition of samkalpa; ix) ending the practice | Pre-intervention  Post-intervention | NA | State trait anxiety inventory  Anxiety scale of Derogatis’s symptomatic checklist  Hostility scale of Derogatis’s symptomatic checklist | **Between-group:** NA  **Within-group:**  Significant ↓ in anxiety (state trait anxiety inventory) with YGN post-intervention  Significant ↓ in anxiety (anxiety scale of Derogatis’s symptomatic checklist) with YGN post-intervention  Significant ↓ in hostility (hostility scale of Derogatis’s symptomatic checklist) with YGN post-intervention  Significant ↓ in anxiety (state trait anxiety inventory) in middle-anxiety scoring group with YGN post-intervention  No difference in anxiety (state trait anxiety inventory) in low-, high-anxiety scoring group with YGN post-intervention  Significant ↓ in anxiety (anxiety scale of Derogatis’s symptomatic checklist) in high-, middle-anxiety scoring group with YGN post-intervention  No difference in anxiety (anxiety scale of Derogatis’s symptomatic checklist) in low-anxiety scoring group with YGN post-intervention  Significant ↓ in hostility (hostility scale of Derogatis’s symptomatic checklist) in high-, middle-anxiety scoring group with YGN post-intervention  No difference in hostility (hostility scale of Derogatis’s symptomatic checklist) in low-anxiety scoring group with YGN post-intervention | **D&B: Poor** |
| Mishra and Sinha (2001) | India | Quasi experimental design (pre vs post) | 12F, 10M (22 to 70)  Gastro-intestinal disorder | Yoga nidra + asanas, pranayama, satkarmas | YGN:  Duration of session: 60 minutes  Number of sessions per week: 7  Number of weeks: 2  Total number of sessions: 15  *yoga nidra part of meditation session that lasted for 60 minutes | Delivery setting: In person  Trained instructor: Yes  Steps: performed under guidance of Swami Niranjananda Saraswati was a part of meditation session that also included, antar mouna, ajapajapa  Participants adopted yogic lifestyle while living in an ashram and also received sattvic food (simple, seasonal diet) | Pre-intervention  Post-intervention | NA | STAI  Depression scale of symptomatic check list | **Between-group:**  NA  **Within-group:**  Significant ↓ in anxiety (STAI) with YGN post intervention  No difference in depression (depression scale of symptomatic check list) with YGN post intervention | **D&B: Poor** |
| BAI: Beck Anxiety Inventory, BDI: Beck Depression Inventory, DASS: Depression anxiety stress scale, GAD-7: Generalized Anxiety Disorder-7 item, STAI: State Trait Anxiety Inventory, NA: Not applicable, NR: Not reported | | | | | | | | | | | |


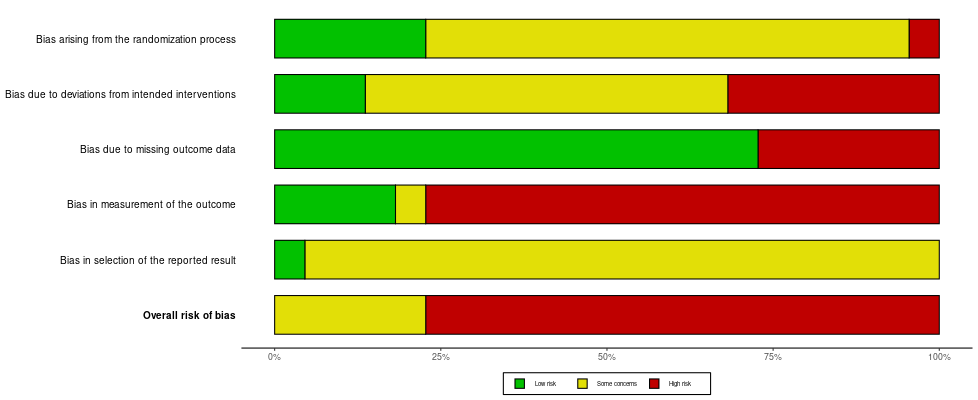


Figure S1. Risk of bias illustration for the randomized controlled trials with by Cochrane Risk of Bias 2 tool

**Between-group analyses**

Figure S2. Forest plot showing the effects of yoga nidra compared to no training on stress outcomes (randomized controlled trials). Each horizontal line represents the 95% confidence interval (CI) for the effect size (Hedges's g) of individual studies. Negative values of Hedges's g indicate a favorable effect of yoga nidra. The size of the red squares reflects the weight of each study in the meta-analysis, and the diamond represents the overall effect size and confidence interval

Figure S3. Forest plot showing the effects of yoga nidra compared to no training on stress outcomes (non-randomized controlled trials). Horizontal lines: 95% CI, red squares: Hedges's g of individual studies, diamond: overall effect size and confidence interval

Figure S4. Forest plot showing the effects of yoga nidra compared to no training on stress outcomes among healthy adults. Horizontal lines: 95% CI, red squares: Hedges's g of individual studies, diamond: overall effect size and confidence interval


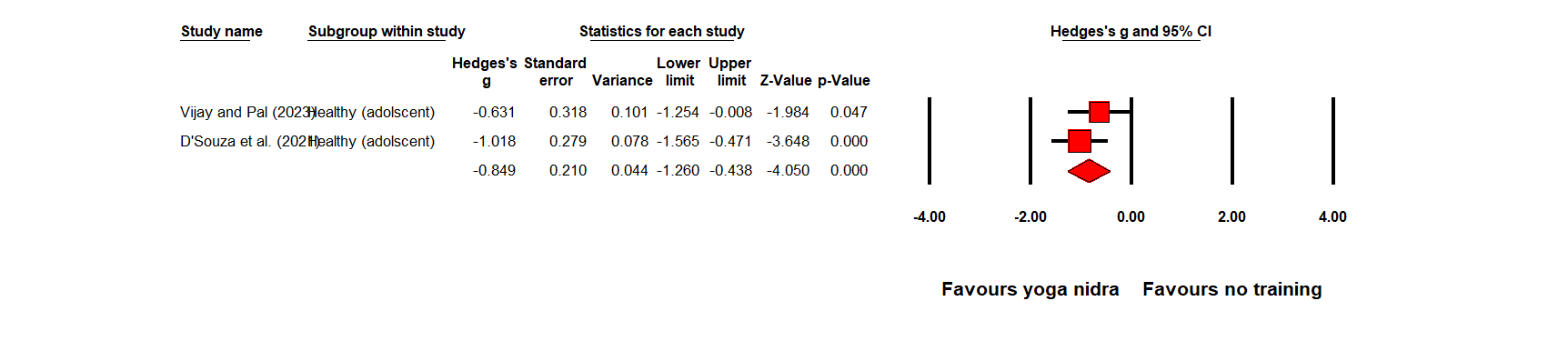


Figure S5. Forest plot showing the effects of yoga nidra compared to no training on stress outcomes among healthy adolescents. Horizontal lines: 95% CI, red squares: Hedges's g of individual studies, diamond: overall effect size and confidence interval

Figure S6. Forest plot showing the effects of yoga nidra compared to a comparator on stress outcomes (randomized controlled trials). Horizontal lines: 95% CI, red squares: Hedges's g of individual studies, diamond: overall effect size and confidence interval

Figure S7. Forest plot showing the effects of yoga nidra compared to a comparator on stress outcomes (non-randomized controlled trials). Horizontal lines: 95% CI, red squares: Hedges's g of individual studies, diamond: overall effect size and confidence interval

Figure S8. Forest plot showing the effects of yoga nidra compared to a comparator on stress outcomes among healthy adults. Horizontal lines: 95% CI, red squares: Hedges's g of individual studies, diamond: overall effect size and confidence interval

Figure S9. Forest plot showing the effects of yoga nidra compared to no training on anxiety outcomes (randomized controlled trials). Horizontal lines: 95% CI, red squares: Hedges's g of individual studies, diamond: overall effect size and confidence interval

Figure S10. Forest plot showing the effects of yoga nidra compared to no training on anxiety outcomes (non-randomized controlled trials). Horizontal lines: 95% CI, red squares: Hedges's g of individual studies, diamond: overall effect size and confidence interval

Figure S11. Forest plot showing the effects of yoga nidra compared to no training on anxiety outcomes among healthy adults. Horizontal lines: 95% CI, red squares: Hedges's g of individual studies, diamond: overall effect size and confidence interval

Figure S12. Forest plot showing the effects of yoga nidra compared to no training on anxiety outcomes among healthy adolescents. Horizontal lines: 95% CI, red squares: Hedges's g of individual studies, diamond: overall effect size and confidence interval

Figure S13. Forest plot showing the effects of yoga nidra compared to comparator on anxiety outcomes (randomized controlled trials). Horizontal lines: 95% CI, red squares: Hedges's g of individual studies, diamond: overall effect size and confidence interval

Figure S14. Forest plot showing the effects of yoga nidra compared to comparator on anxiety outcomes (non-randomized controlled trials). Horizontal lines: 95% CI, red squares: Hedges's g of individual studies, diamond: overall effect size and confidence interval

Figure S15. Forest plot showing the effects of yoga nidra compared to comparator on anxiety outcomes among healthy adults. Horizontal lines: 95% CI, red squares: Hedges's g of individual studies, diamond: overall effect size and confidence interval

Figure S16. Forest plot showing the effects of yoga nidra compared to comparator on anxiety outcomes among adults with menstrual disorders. Horizontal lines: 95% CI, red squares: Hedges's g of individual studies, diamond: overall effect size and confidence interval

Figure S17. Forest plot showing the effects of yoga nidra compared to comparator on anxiety outcomes among adults with hypertension. Horizontal lines: 95% CI, red squares: Hedges's g of individual studies, diamond: overall effect size and confidence interval

Figure S18. Forest plot showing the effects of yoga nidra compared to no training on depression outcomes (randomized controlled trials). Horizontal lines: 95% CI, red squares: Hedges's g of individual studies, diamond: overall effect size and confidence interval

Figure S19. Forest plot showing the effects of yoga nidra compared to no training on depression outcomes (non-randomized controlled trials). Horizontal lines: 95% CI, red squares: Hedges's g of individual studies, diamond: overall effect size and confidence interval

Figure S20. Forest plot showing the effects of yoga nidra compared to no training depression outcomes among healthy adults. Horizontal lines: 95% CI, red squares: Hedges's g of individual studies, diamond: overall effect size and confidence interval

Figure S21. Forest plot showing the effects of yoga nidra compared to comparator on depression outcomes (randomized controlled trials). Horizontal lines: 95% CI, red squares: Hedges's g of individual studies, diamond: overall effect size and confidence interval


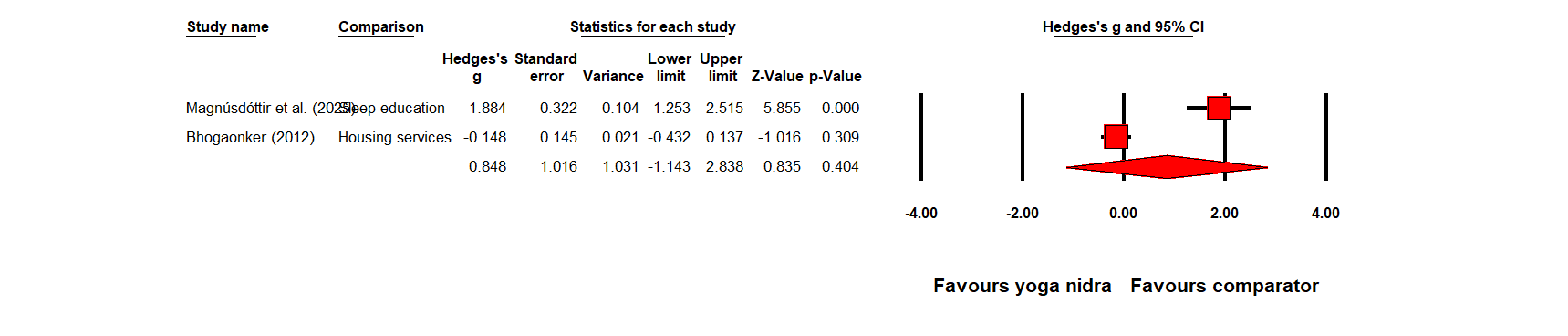


Figure S22. Forest plot showing the effects of yoga nidra compared to comparator on depression outcomes (non-randomized controlled trials). Horizontal lines: 95% CI, red squares: Hedges's g of individual studies, diamond: overall effect size and confidence interval


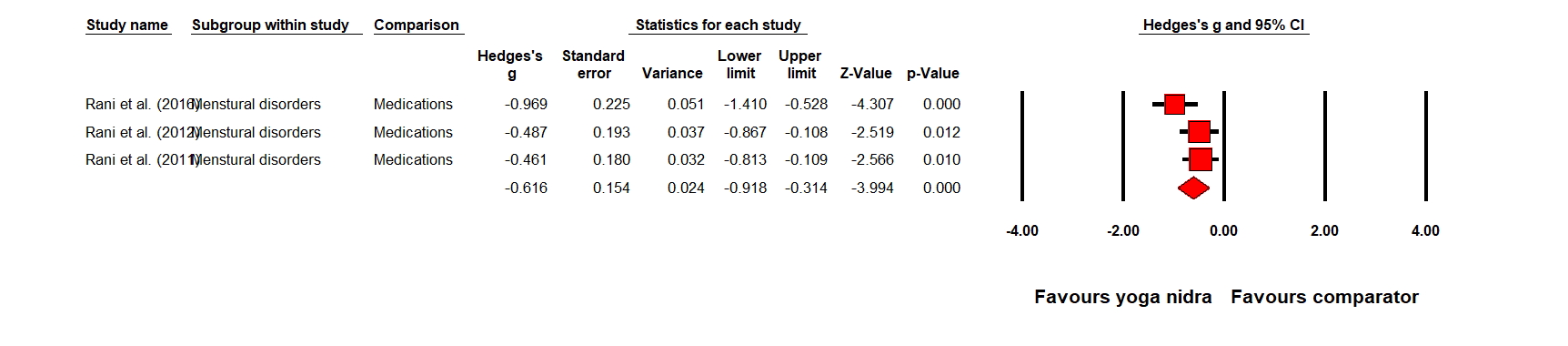


Figure S23. Forest plot showing the effects of yoga nidra compared to comparator on depression outcomes among adults with menstural disorders. Horizontal lines: 95% CI, red squares: Hedges's g of individual studies, diamond: overall effect size and confidence interval

Figure S24. Forest plot showing the effects of yoga nidra compared to comparator on depression outcomes among healthy adults. Horizontal lines: 95% CI, red squares: Hedges's g of individual studies, diamond: overall effect size and confidence interval

**Within-group analyses**

Figure S25. Forest plot showing the within group effects (pre vs. post) of yoga nidra on stress outcomes. Horizontal lines: 95% CI, red squares: Hedges's g of individual studies, diamond: overall effect size and confidence interval

Figure S26. Forest plot showing the within group effects (pre vs. post) of yoga nidra on stress outcomes (randomized controlled trials). Horizontal lines: 95% CI, red squares: Hedges's g of individual studies, diamond: overall effect size and confidence interval

Figure S27. Forest plot showing the within group effects (pre vs. post) of yoga nidra on stress outcomes (non-randomized controlled trials). Horizontal lines: 95% CI, red squares: Hedges's g of individual studies, diamond: overall effect size and confidence interval

Figure S28. Forest plot showing the within group effects (pre vs. post) of yoga nidra on stress outcomes in healthy adults. Horizontal lines: 95% CI, red squares: Hedges's g of individual studies, diamond: overall effect size and confidence interval

Figure S29. Forest plot showing the within group effects (pre vs. post) of yoga nidra on stress outcomes in healthy adolescents. Horizontal lines: 95% CI, red squares: Hedges's g of individual studies, diamond: overall effect size and confidence interval

Figure S30. Forest plot showing the within group effects (pre vs. post) of yoga nidra on stress outcomes in adults with hypertension. Horizontal lines: 95% CI, red squares: Hedges's g of individual studies, diamond: overall effect size and confidence interval

Figure S31. Forest plot showing the within group effects (pre vs. post) of yoga nidra on stress outcomes in adults with cancer. Horizontal lines: 95% CI, red squares: Hedges's g of individual studies, diamond: overall effect size and confidence interval

Figure S32. Forest plot showing the within group effects (pre vs. post) of yoga nidra on anxiety outcomes. Horizontal lines: 95% CI, red squares: Hedges's g of individual studies, diamond: overall effect size and confidence interval

Figure S33. Forest plot showing the within group effects (pre vs. post) of yoga nidra on anxiety outcomes (randomized controlled trials). Horizontal lines: 95% CI, red squares: Hedges's g of individual studies, diamond: overall effect size and confidence interval

Figure S34. Forest plot showing the within group effects (pre vs. post) of yoga nidra on anxiety outcomes (non-randomized controlled trials). Horizontal lines: 95% CI, red squares: Hedges's g of individual studies, diamond: overall effect size and confidence interval

Figure S35. Forest plot showing the within group effects (pre vs. post) of yoga nidra on anxiety outcomes in healthy adults. Horizontal lines: 95% CI, red squares: Hedges's g of individual studies, diamond: overall effect size and confidence interval

Figure S36. Forest plot showing the within group effects (pre vs. post) of yoga nidra on anxiety outcomes in healthy adolescents. Horizontal lines: 95% CI, red squares: Hedges's g of individual studies, diamond: overall effect size and confidence interval

Figure S37. Forest plot showing the within group effects (pre vs. post) of yoga nidra on anxiety outcomes in adults with menstrual disorders. Horizontal lines: 95% CI, red squares: Hedges's g of individual studies, diamond: overall effect size and confidence interval

Figure S38. Forest plot showing the within group effects (pre vs. post) of yoga nidra on anxiety outcomes in adults with insomnia. Horizontal lines: 95% CI, red squares: Hedges's g of individual studies, diamond: overall effect size and confidence interval

Figure S39. Forest plot showing the within group effects (pre vs. post) of yoga nidra on depression outcomes. Horizontal lines: 95% CI, red squares: Hedges's g of individual studies, diamond: overall effect size and confidence interval

Figure S40. Forest plot showing the within group effects (pre vs. post) of yoga nidra on depression outcomes (randomized controlled trials). Horizontal lines: 95% CI, red squares: Hedges's g of individual studies, diamond: overall effect size and confidence interval

Figure S41. Forest plot showing the within group effects (pre vs. post) of yoga nidra on depression outcomes (non-randomized controlled trials). Horizontal lines: 95% CI, red squares: Hedges's g of individual studies, diamond: overall effect size and confidence interval

Figure S42. Forest plot showing the within group effects (pre vs. post) of yoga nidra on depression outcomes in healthy adults. Horizontal lines: 95% CI, red squares: Hedges's g of individual studies, diamond: overall effect size and confidence interval

Figure S43. Forest plot showing the within group effects (pre vs. post) of yoga nidra on depression outcomes in healthy adolescents. Horizontal lines: 95% CI, red squares: Hedges's g of individual studies, diamond: overall effect size and confidence interval

Figure S44. Forest plot showing the within group effects (pre vs. post) of yoga nidra on depression outcomes in adults with depression. Horizontal lines: 95% CI, red squares: Hedges's g of individual studies, diamond: overall effect size and confidence interval

Figure S45. Forest plot showing the within group effects (pre vs. post) of yoga nidra on depression outcomes in adults with menstrual disorders. Horizontal lines: 95% CI, red squares: Hedges's g of individual studies, diamond: overall effect size and confidence interval

**Leave one out sensitivity analysis**

Figure S46. Forest plot showing the leave-one-out sensitivity analysis of the between group effects of yoga nidra (vs. no training) on stress outcomes. Horizontal lines: 95% CI, red squares: Hedges's g of individual studies, diamond: overall effect size and confidence interval

Figure S47. Forest plot showing the leave-one-out sensitivity analysis of the between group effects of yoga nidra (vs. no training) on stress outcomes (randomized controlled trials). Horizontal lines: 95% CI, red squares: Hedges's g of individual studies, diamond: overall effect size and confidence interval

Figure S48. Forest plot showing the leave-one-out sensitivity analysis of the between group effects of yoga nidra (vs. no training) on stress outcomes (non-randomized controlled trials). Horizontal lines: 95% CI, red squares: Hedges's g of individual studies, diamond: overall effect size and confidence interval

Figure S49. Forest plot showing the leave-one-out sensitivity analysis of the between group effects of yoga nidra (vs. no training) on stress outcomes in healthy adults. Horizontal lines: 95% CI, red squares: Hedges's g of individual studies, diamond: overall effect size and confidence interval


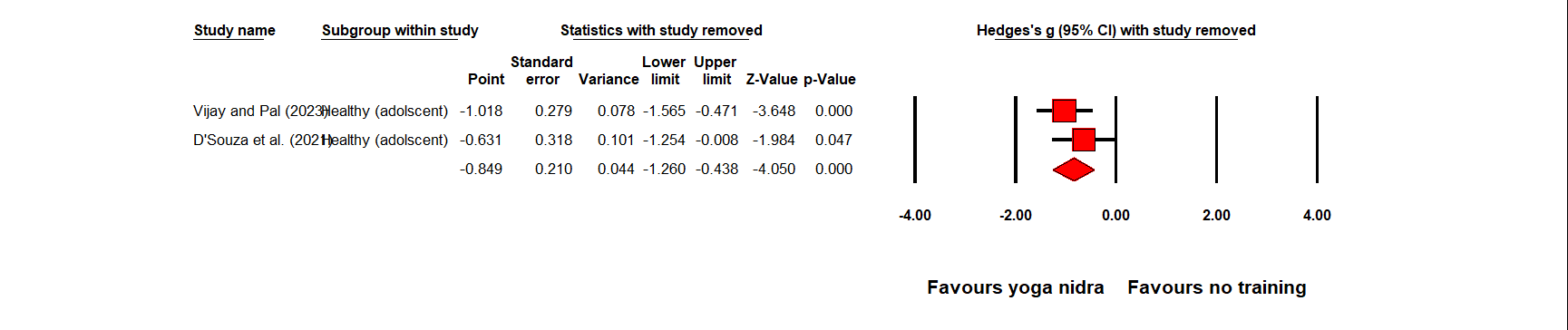


Figure S50. Forest plot showing the leave-one-out sensitivity analysis of the between group effects of yoga nidra (vs. no training) on stress outcomes in healthy adolescents. Horizontal lines: 95% CI, red squares: Hedges's g of individual studies, diamond: overall effect size and confidence interval

Figure S51. Forest plot showing the leave-one-out sensitivity analysis of the between group effects of yoga nidra (vs. comparator) on stress outcomes. Horizontal lines: 95% CI, red squares: Hedges's g of individual studies, diamond: overall effect size and confidence interval

Figure S52. Forest plot showing the leave-one-out sensitivity analysis of the between group effects of yoga nidra (vs. comparator) on stress outcomes (randomized controlled trials). Horizontal lines: 95% CI, red squares: Hedges's g of individual studies, diamond: overall effect size and confidence interval

Figure S53. Forest plot showing the leave-one-out sensitivity analysis of the between group effects of yoga nidra (vs. comparator) on stress outcomes (non-randomized controlled trials). Horizontal lines: 95% CI, red squares: Hedges's g of individual studies, diamond: overall effect size and confidence interval

Figure S54. Forest plot showing the leave-one-out sensitivity analysis of the between group effects of yoga nidra (vs. comparator) on stress outcomes in healthy adults. Horizontal lines: 95% CI, red squares: Hedges's g of individual studies, diamond: overall effect size and confidence interval

Figure S55. Forest plot showing the leave-one-out sensitivity analysis of the between group effects of yoga nidra (vs. no training) on anxiety outcomes. Horizontal lines: 95% CI, red squares: Hedges's g of individual studies, diamond: overall effect size and confidence interval

Figure S56. Forest plot showing the leave-one-out sensitivity analysis of the between group effects of yoga nidra (vs. no training) on anxiety outcomes (randomized controlled trials). Horizontal lines: 95% CI, red squares: Hedges's g of individual studies, diamond: overall effect size and confidence interval

Figure S57. Forest plot showing the leave-one-out sensitivity analysis of the between group effects of yoga nidra (vs. no training) on anxiety outcomes (non-randomized controlled trials). Horizontal lines: 95% CI, red squares: Hedges's g of individual studies, diamond: overall effect size and confidence interval

Figure S58. Forest plot showing the leave-one-out sensitivity analysis of the between group effects of yoga nidra (vs. no training) on anxiety outcomes in healthy adults. Horizontal lines: 95% CI, red squares: Hedges's g of individual studies, diamond: overall effect size and confidence interval

Figure S59. Forest plot showing the leave-one-out sensitivity analysis of the between group effects of yoga nidra (vs. no training) on anxiety outcomes in healthy adolescents. Horizontal lines: 95% CI, red squares: Hedges's g of individual studies, diamond: overall effect size and confidence interval

Figure S60. Forest plot showing the leave-one-out sensitivity analysis of the between group effects of yoga nidra (vs. comparator) on anxiety outcomes. Horizontal lines: 95% CI, red squares: Hedges's g of individual studies, diamond: overall effect size and confidence interval

Figure S61. Forest plot showing the leave-one-out sensitivity analysis of the between group effects of yoga nidra (vs. comparator) on anxiety outcomes (randomized controlled trials). Horizontal lines: 95% CI, red squares: Hedges's g of individual studies, diamond: overall effect size and confidence interval

Figure S62. Forest plot showing the leave-one-out sensitivity analysis of the between group effects of yoga nidra (vs. comparator) on anxiety outcomes (non-randomized controlled trials). Horizontal lines: 95% CI, red squares: Hedges's g of individual studies, diamond: overall effect size and confidence interval

Figure S63. Forest plot showing the leave-one-out sensitivity analysis of the between group effects of yoga nidra (vs. comparator) on anxiety outcomes in healthy adults. Horizontal lines: 95% CI, red squares: Hedges's g of individual studies, diamond: overall effect size and confidence interval

Figure S64. Forest plot showing the leave-one-out sensitivity analysis of the between group effects of yoga nidra (vs. comparator) on anxiety outcomes in adults with menstrual disorders. Horizontal lines: 95% CI, red squares: Hedges's g of individual studies, diamond: overall effect size and confidence interval

Figure S65. Forest plot showing the leave-one-out sensitivity analysis of the between group effects of yoga nidra (vs. comparator) on anxiety outcomes in adults with hypertension. Horizontal lines: 95% CI, red squares: Hedges's g of individual studies, diamond: overall effect size and confidence interval

Figure S66. Forest plot showing the leave-one-out sensitivity analysis of the between group effects of yoga nidra (vs. no training) on depression outcomes. Horizontal lines: 95% CI, red squares: Hedges's g of individual studies, diamond: overall effect size and confidence interval

Figure S67. Forest plot showing the leave-one-out sensitivity analysis of the between group effects of yoga nidra (vs. no training) on depression outcomes (randomized controlled trials). Horizontal lines: 95% CI, red squares: Hedges's g of individual studies, diamond: overall effect size and confidence interval

Figure S68. Forest plot showing the leave-one-out sensitivity analysis of the between group effects of yoga nidra (vs. no training) on depression outcomes (non-randomized controlled trials). Horizontal lines: 95% CI, red squares: Hedges's g of individual studies, diamond: overall effect size and confidence interval

Figure S69. Forest plot showing the leave-one-out sensitivity analysis of the between group effects of yoga nidra (vs. no training) on depression outcomes in healthy adults. Horizontal lines: 95% CI, red squares: Hedges's g of individual studies, diamond: overall effect size and confidence interval

Figure S70. Forest plot showing the leave-one-out sensitivity analysis of the between group effects of yoga nidra (vs. comparator) on depression outcomes. Horizontal lines: 95% CI, red squares: Hedges's g of individual studies, diamond: overall effect size and confidence interval

Figure S71. Forest plot showing the leave-one-out sensitivity analysis of the between group effects of yoga nidra (vs. comparator) on depression outcomes (randomized controlled trials). Horizontal lines: 95% CI, red squares: Hedges's g of individual studies, diamond: overall effect size and confidence interval


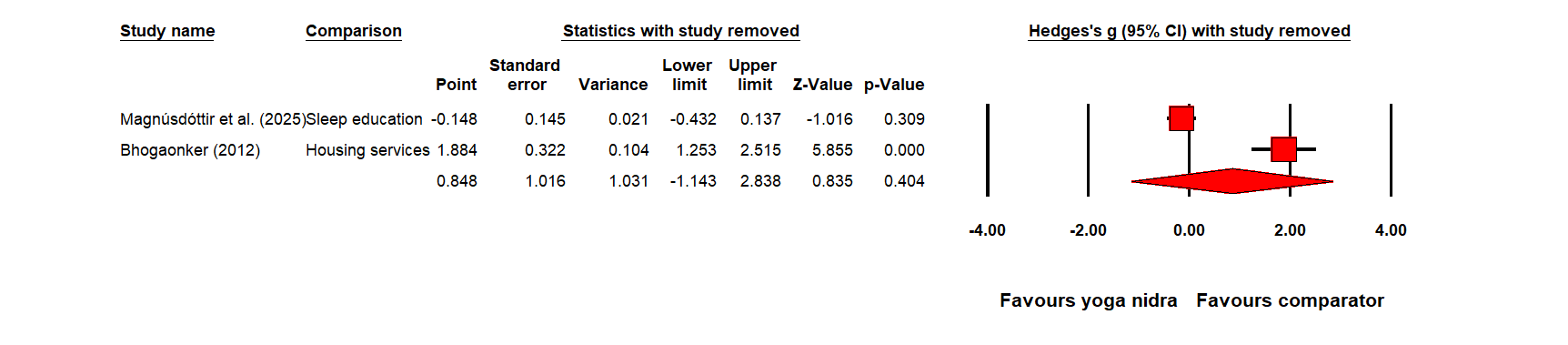


Figure S72. Forest plot showing the leave-one-out sensitivity analysis of the between group effects of yoga nidra (vs. comparator) on depression outcomes (non-randomized controlled trials). Horizontal lines: 95% CI, red squares: Hedges's g of individual studies, diamond: overall effect size and confidence interval


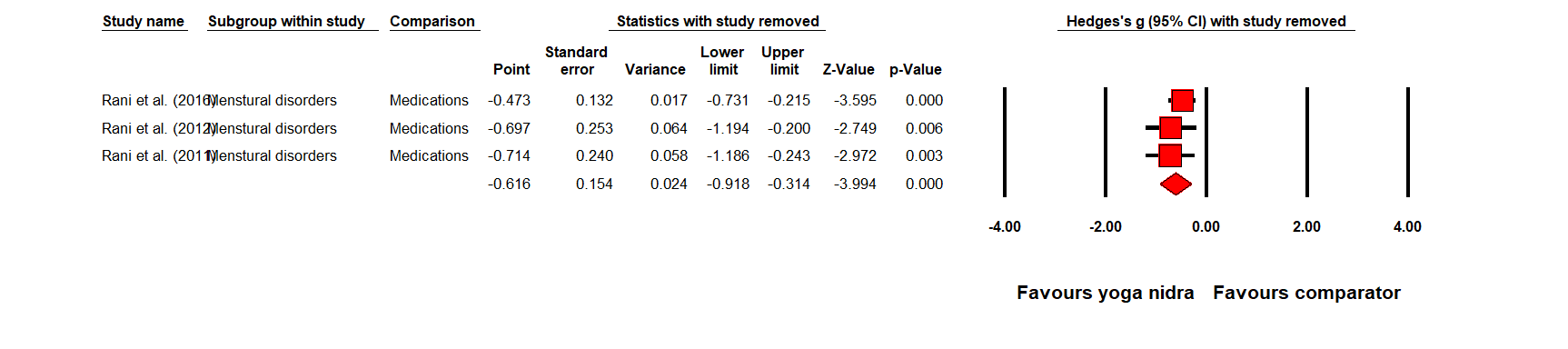


Figure S73. Forest plot showing the leave-one-out sensitivity analysis of the between group effects of yoga nidra (vs. comparator) on depression outcomes in adults with menstrual disorders. Horizontal lines: 95% CI, red squares: Hedges's g of individual studies, diamond: overall effect size and confidence interval

Figure S74. Forest plot showing the leave-one-out sensitivity analysis of the between group effects of yoga nidra (vs. comparator) on depression outcomes in healthy adults. Horizontal lines: 95% CI, red squares: Hedges's g of individual studies, diamond: overall effect size and confidence interval

Figure S75. Forest plot showing the leave-one-out sensitivity analysis of the within group (pre vs. post) effects of yoga nidra on stress outcomes. Horizontal lines: 95% CI, red squares: Hedges's g of individual studies, diamond: overall effect size and confidence interval

Figure S76. Forest plot showing the leave-one-out sensitivity analysis of the within group (pre vs. post) effects of yoga nidra on stress outcomes (randomized controlled trials). Horizontal lines: 95% CI, red squares: Hedges's g of individual studies, diamond: overall effect size and confidence interval

Figure S77. Forest plot showing the leave-one-out sensitivity analysis of the within group (pre vs. post) effects of yoga nidra on stress outcomes (non-randomized controlled trials). Horizontal lines: 95% CI, red squares: Hedges's g of individual studies, diamond: overall effect size and confidence interval

Figure S78. Forest plot showing the leave-one-out sensitivity analysis of the within group (pre vs. post) effects of yoga nidra on stress outcomes in healthy adults. Horizontal lines: 95% CI, red squares: Hedges's g of individual studies, diamond: overall effect size and confidence interval

Figure S79. Forest plot showing the leave-one-out sensitivity analysis of the within group (pre vs. post) effects of yoga nidra on stress outcomes in healthy adolescents. Horizontal lines: 95% CI, red squares: Hedges's g of individual studies, diamond: overall effect size and confidence interval

Figure S80. Forest plot showing the leave-one-out sensitivity analysis of the within group (pre vs. post) effects of yoga nidra on stress outcomes in adults with hypertension. Horizontal lines: 95% CI, red squares: Hedges's g of individual studies, diamond: overall effect size and confidence interval

Figure S81. Forest plot showing the leave-one-out sensitivity analysis of the within group (pre vs. post) effects of yoga nidra on stress outcomes in adults with cancer. Horizontal lines: 95% CI, red squares: Hedges's g of individual studies, diamond: overall effect size and confidence interval

Figure S82. Forest plot showing the leave-one-out sensitivity analysis of the within group (pre vs. post) effects of yoga nidra on anxiety outcomes. Horizontal lines: 95% CI, red squares: Hedges's g of individual studies, diamond: overall effect size and confidence interval

Figure S83. Forest plot showing the leave-one-out sensitivity analysis of the within group (pre vs. post) effects of yoga nidra on anxiety outcomes (randomized controlled trials). Horizontal lines: 95% CI, red squares: Hedges's g of individual studies, diamond: overall effect size and confidence interval

Figure S84. Forest plot showing the leave-one-out sensitivity analysis of the within group (pre vs. post) effects of yoga nidra on anxiety outcomes (non-randomized controlled trials). Horizontal lines: 95% CI, red squares: Hedges's g of individual studies, diamond: overall effect size and confidence interval

Figure S85. Forest plot showing the leave-one-out sensitivity analysis of the within group (pre vs. post) effects of yoga nidra on anxiety outcomes in healthy adults. Horizontal lines: 95% CI, red squares: Hedges's g of individual studies, diamond: overall effect size and confidence interval

Figure S86. Forest plot showing the leave-one-out sensitivity analysis of the within group (pre vs. post) effects of yoga nidra on anxiety outcomes in healthy adolescents. Horizontal lines: 95% CI, red squares: Hedges's g of individual studies, diamond: overall effect size and confidence interval

Figure S87. Forest plot showing the leave-one-out sensitivity analysis of the within group (pre vs. post) effects of yoga nidra on anxiety outcomes in adults with menstrual disorders. Horizontal lines: 95% CI, red squares: Hedges's g of individual studies, diamond: overall effect size and confidence interval

Figure S88. Forest plot showing the leave-one-out sensitivity analysis of the within group (pre vs. post) effects of yoga nidra on anxiety outcomes in adults with insomnia. Horizontal lines: 95% CI, red squares: Hedges's g of individual studies, diamond: overall effect size and confidence interval

Figure S89. Forest plot showing the leave-one-out sensitivity analysis of the within group (pre vs. post) effects of yoga nidra on depression. Horizontal lines: 95% CI, red squares: Hedges's g of individual studies, diamond: overall effect size and confidence interval

Figure S90. Forest plot showing the leave-one-out sensitivity analysis of the within group (pre vs. post) effects of yoga nidra on depression (randomized controlled trials). Horizontal lines: 95% CI, red squares: Hedges's g of individual studies, diamond: overall effect size and confidence interval

Figure S91. Forest plot showing the leave-one-out sensitivity analysis of the within group (pre vs. post) effects of yoga nidra on depression (non-randomized controlled trials). Horizontal lines: 95% CI, red squares: Hedges's g of individual studies, diamond: overall effect size and confidence interval

Figure S92. Forest plot showing the leave-one-out sensitivity analysis of the within group (pre vs. post) effects of yoga nidra on depression in healthy adults. Horizontal lines: 95% CI, red squares: Hedges's g of individual studies, diamond: overall effect size and confidence interval

Figure S93. Forest plot showing the leave-one-out sensitivity analysis of the within group (pre vs. post) effects of yoga nidra on depression in healthy adolescents. Horizontal lines: 95% CI, red squares: Hedges's g of individual studies, diamond: overall effect size and confidence interval

Figure S94. Forest plot showing the leave-one-out sensitivity analysis of the within group (pre vs. post) effects of yoga nidra on depression in adults with depression. Horizontal lines: 95% CI, red squares: Hedges's g of individual studies, diamond: overall effect size and confidence interval

Figure S95. Forest plot showing the leave-one-out sensitivity analysis of the within group (pre vs. post) effects of yoga nidra on depression in adults with menstrual disorders. Horizontal lines: 95% CI, red squares: Hedges's g of individual studies, diamond: overall effect size and confidence interval

**References**

Anderson, R., Mammen, K., Paul, P., Pletch, A., & Pulia, K. (2017). Using yoga nidra to improve stress in psychiatric nurses in a pilot study. *The Journal of Alternative and Complementary Medicine*, *23*(6), 494-495.

Anuja. (2011). To Study the Effect of Yoga Nidra on Anxiety. *Quest - The Journal of UGC - ASC Nainital*, *5*(2), 288-291. <http://doi.org/10.5958/j.0974-5041.5.2.031>

Barbuto, I. G. (2017). *Effects of Integrative Restoration iRest on Perceived Stress in Workers* Northwest University].

Barik, C. (2023). Immediate effect of yoga nidra technique for physiological variable resting pulse rate, blood pressure, hemoglobin, vital capacity, breath holding, physical fitness index and recovery periods. *International Journal of Science and Research*, *12*(2). <https://doi.org/DOI>: 10.21275/SR23203152048

Bhogaonker, P. (2012). *Impact of brief meditation training on stress, distress, and quality of life for homeless adults*. California Institute of Integral Studies.

Bhushan, S., & Sinha, P. (2001). Yoganidra and management of anxiety and hostility. *Journal of Indian Psychology*, *19*(1/2), 44-49.

Birdsall, B., Pritchard, M., Elison-Bowers, P., & Spann, J. (2011). *Does integrative restoration (iRest) meditation decrease perceived stress levels and negative moods in school counselors*. <http://counselingoutfitters.com/vistas/vistas11/Article_84.pdf>

Chaudhary, N., & Pal, V. K. (2016). A study on the effect of Yoga Nidra on stress level of the patients suffering with spondolitis and backache. *International Journal of Yoga and Allied Sciences*, *5*(1), 24-26.

Chowdhary, B. (2013). Effect of Yoga Nidra and Pranayama on Stress and Social Adjustment Capacity of Tribal Students. *International Journal of Physical and Social Sciences*, *3*(11), 87-95.

D’cunha, R., Pappachan, B., D’souza, O. L., Tonse, R., Saldanha, E., & Baliga, M. S. (2021). Effectiveness of yoga nidra in mitigating stress in women undergoing curative radiotherapy for cervical cancer. *Middle East Journal of Cancer*, *12*(1), 117-127.

D'Souza, O. L., Jose, A. E., Suresh, S., & Baliga, M. S. (2021). Effectiveness of Yoga Nidra in reducing stress in school going adolescents: An experimental study. *Complementary Therapies in Clinical Practice*, *45*, 101462. <https://doi.org/https://doi.org/10.1016/j.ctcp.2021.101462>

De Jesus, S., Schultz, E., & Bond, R. M. (2019). The Yoga–Meditation Heart Connection: A Pilot Study Looking to Improve Women's Heart Health. *Annals of Clinical Cardiology*, *1*(1), 24-29.

Deuskar, M. (2010, 2010). Stress reduction through yoga Nidra. Yoga in Science – Future and Perspectives, Belgrade.

di Fronso, S., Robazza, C., Pompa, D., & Bertollo, M. (2024). Dreaming while awake: The beneficial effects of yoga Nidra on mental and physical recovery in two elite karate athletes. *Heliyon*, *10*(1).

Diener, E., Suh, E. M., Lucas, R. E., & Smith, H. L. (1999). Subjective well-being: three decades of progress. *Psychological bulletin*, *125*(2), 276.

Dol, K. S. (2019). Effects of a yoga nidra on the life stress and self-esteem in university students. *Complementary Therapies in Clinical Practice*, *35*, 232-236. <https://doi.org/https://doi.org/10.1016/j.ctcp.2019.03.004>

Dwivedi, M. K. (2021). Mitigation of stress through yoga nidra (meditation) intervention. *The Journal of Mental Health Training, Education and Practice*, *16*(4), 300-312.

Eastman-Mueller, H., Wilson, T., Jung, A.-K., Kimura, A., & Tarrant, J. (2013). iRest Yoga-Nidra on the College Campus: Changes in Stress, Depression, Worry, and Mindfulness. *International Journal of Yoga Therapy*, *23*(2), 15-24. <https://doi.org/10.17761/ijyt.23.2.r8735770101m8277>

Ferguson, K. L. (2016). *The effects of a yoga nidra practice on mental health clinicians' perceived stress* Smith College]. Northampton, MA. <https://scholarworks.smith.edu/theses/1702>

Ferreira-Vorkapic, C., Borba-Pinheiro, C. J., Marchioro, M., & Santana, D. (2018). The impact of yoga nidra and seated meditation on the mental health of college professors. *International journal of yoga*, *11*(3), 215-223.

Foulkrod, K., Griesemer, S., Banneyer, K. N., & Caemmerer, J. M. (2016). Yoga Plus Talk Therapy for Depression: A Case Study of a Six Week Group. *International Journal of Integrative Psychotherapy*, *7*.

Genovese, J. E. C., & Fondran, K. M. (2016). Depression and anxiety decline after participation in a semester long yoga class. *Psychology and Education: An Interdisciplinary Journal*, 52-54.

Graham, M. (2022). Effect of Yoga Nidra on Reducing Stress, Increasing Attention, Changing Behavior and Increasing Happiness of School Students. *Journal of Innovation and Social Science Research 9*(4), 239-245. <https://doi.org/DOI>: 10.53469/jissr.2022.09(04).49

Gunjiganvi, M., Rai, S., Awale, R., Mishra, P., Gupta, D., & Gurjar, M. (2023). Efficacy of Yoga Nidra on depression, anxiety, and insomnia in frontline COVID-19 healthcare workers: a pilot randomized controlled trial. *International Journal of Yoga Therapy*, *33*(2023).

Gupta, P., Deshpande, K., & Shamrao, K. S. (2022). Study of the Effect of Yoga-Nidra on the Psychology of Healthcare Workers in Terms of Depression, Anxiety and Stress Scale. *Bull. Env. Pharmacol. Life Sci*, *12*, 160-169.

Jaiganesh, D. K., Parthasarathy, D. S., & Duraisamy, M. (2022). Effect of Suryanamaskar Practices with and Without Yoga Nidra On Stress Among High School Students-A Randomized Controlled Trial.(2022). *International Journal of Life science and Pharma Research*, *12*(6), L28-33.

Jensen, P. S., Stevens, P. J., & Kenny, D. T. (2012). Respiratory patterns in students enrolled in schools for disruptive behaviour before, during, and after yoga nidra relaxation. *Journal of Child and Family Studies*, *21*, 667-681.

Joshi, R. (2020). Anxiety and depression related to yog nidra among professional students. *Yoga Mimamsa*, *52*(1). <https://journals.lww.com/yomi/fulltext/2020/52010/anxiety_and_depression_related_to_yog_nidra_among.6.aspx>

K., B. L. (2008). *Effect of Cyclic Meditation and Yoga Nidra on Selected Physiological and Psychological Variables in College Level Athletes* University of Calicut]. Kerala.

Kalita, J. (2021). Effect of yoga-nidra programme on depression, anxiety, and stress among patients with chronic kidney disease receiving haemodialysis. *International Journal of Health Sciences and Research*, *11*(3), 38-43.

Kalita, J., & Choudhury, P. (2022). A pilot study on effect of yoga-nidra programme on

depression, anxiety, and stress among patients with

chronic kidney disease receiving haemodialysis. *International Journal of Applied Research*, *8*(3), 346-349.

Kamble, P., Daulatabad, V. S., Tandra, H., Singhal, A., Madhusudhan, U., & John, N. A. (2023). Yoganidra to Alleviate Anxiety: An Interventional Study. *Cureus*, *15*(9). <https://doi.org/http://doi.org/10.7759/cureus.45083>

Kannan, S. D., & Kumar, R. (2021). Case Study Report – Combination Effect of Yoga Nidra and Pranayama in Managing the Anxiety Disorder. *International Journal of Indian Psychology*, *9*(3), 1202-1209. <https://doi.org/10.25215/0903.112>

Kaur, R., & Sharma, S. (2021). Effect of Yoganidra on Anxiety and Depressive Symptoms in Working Women. *International Research Journal of Ayurveda and Yoga*, *4*(11), 49-52.

Kumar, A., Sahu, M., & Yadav, A. (2024). A study on the effect of Yoga Nidra on Anxiety in Pregnant Women of different Trimester. *Journal of Ayurveda and Integrated Medical Sciences*, *9*(5), 21-24.

Kumar, K. (2004). Yoga nidra and its impact on student’s well being. *Yoga Mimamsha, Kaivalyadhama, Lonavla*, *36*(1).

Kumar, K. (2008). A study on the impact on stress and anxiety through Yoga nidra [Journal article]. *Indian Journal of Traditional Knowledge*, *7*(3), 401–404.

Lakshmipathy, S., & Easvaradoss, V. (2018). Impact of Yoga Nidra Meditation on Perceived Stress and Self Regulation in Teacher Trainees. *International Journal of Indian Psychology*, *6*(3), 89-95.

Livingston, E., & Collette-Merrill, K. (2018). Effectiveness of integrative restoration (iRest) yoga nidra on mindfulness, sleep, and pain in health care workers. *Holistic nursing practice*, *32*(3), 160-166. <https://www.ingentaconnect.com/content/wk/hnp/2018/00000032/00000003/art00008;jsessionid=pe25vh4i3e2y.x-ic-live-03>

Lukács, A., Mayer, K., Szalkai, I., & Barkai, L. (2012). Impact of yoga nidra on students' wellbeing. In M. Egyetem (Ed.), *microCAD 2012, S section* (Vol. S10). Miskolc. <https://real.mtak.hu/22425/1/yoga_nidra_u_141106.874023.pdf>

Magnúsdóttir, I., Magnúsdóttir, S., Gunnlaugsdóttir, A. K., Hilmisson, H., Hrólfsdóttir, L., & Eiriksdóttir, A. E. (2025). Efficacy of brief behavioral and sleep hygiene education with mindfulness intervention on sleep, social jetlag and mental health in adolescence: a pilot study. *Sleep and Breathing*, *29*(1), 81. <https://doi.org/10.1007/s11325-024-03238-3>

Manik, R., & Gartia, R. (2016). A comparative study of Yoga Nidra and Nadisodhana Pranayam on essential hypertension. *Advanced Science Letters*, *22*(2), 437-441. <https://doi.org/https://doi.org/10.1166/asl.2016.6834>

Mishra, M., & Sinha, R. K. (2001). Effect of Yogic Practices on Depression and Anixety. *SIS Journal of Projective Psychology & Mental Health*, *3*(1), 23.

Moszeik, E. N., Rohleder, N., & Renner, K. H. (2025). The Effects of an Online Yoga Nidra Meditation on Subjective Well‐Being and Diurnal Salivary Cortisol: A Randomised Controlled Trial. *Stress and Health*, *41*(3), e70049. <https://onlinelibrary.wiley.com/doi/pdfdirect/10.1002/smi.70049?download=true>

Moszeik, E. N., von Oertzen, T., & Renner, K.-H. (2022). Effectiveness of a short Yoga Nidra meditation on stress, sleep, and well-being in a large and diverse sample. *Current Psychology*, *41*(8), 5272-5286. <https://doi.org/https://doi.org/10.1007/s12144-020-01042-2>

Muley, P. P., Muley, P. A., Mandlik, V. V., Deshpande, V. P., & Bandre, G. (2024). Effect of 2 Weeks of Yoga Nidra on Stress Relaxation Rating Scale in Medical Graduates. *Journal of Datta Meghe Institute of Medical Sciences University*, *19*(3), 591-594.

Navarange, S., Wasnik, V., & Jain, S. (2023). Effect of Shirodhara and Yoga Nidra in Generalised Anxiety Disorder: A Single Case Study. *Journal of Ayurveda and Integrated Medical Sciences*, *8*(3), 183-187.

Neha, & Kumar, Y. (2023). Effect of yoga nidra on anxiety among male and female college students. *International Journal of Scientific Development and Research*, *8*(1), 1094-1098.

Nuzhath, F. J., Patil, N. J., Sheela, S. R., Manjunath, G. N., & Nuzhath, F. J. (2024). A randomized controlled trial on pranayama and Yoga Nidra for anxiety and depression in patients with cervical cancer undergoing standard of care. *Cureus*, *16*(3).

Panigrahi, M., Shree, P., Swain, D. P., & Biswas, M. (2024). Effect of Yoga Nidra with Some Selective Pranayama’s on Stress Level of Senior Citizens. *African Journal of Biomedical Research*, *27*, 2315-2320. <https://doi.org/https://doi.org/10.53555/AJBR.v27i3S.2632>

Pence, P., Katz, L., Huffman, C., & Cojucar, G. (2014). Delivering Integrative Restoration-Yoga Nidra Meditation (iRest®) to Women with Sexual Trauma at a Veteran's Medical Center: A Pilot Study. *International Journal of Yoga Therapy*, *24*, 53-62. <https://doi.org/10.17761/ijyt.24.1.u7747w56066vq78u>

Pritchard, M., Elison-Bowers, P., & Birdsall, B. (2010). Impact of integrative restoration (iRest) meditation on perceived stress levels in multiple sclerosis and cancer outpatients. *Stress and Health*, *26*(3), 233-237. <https://doi.org/https://doi.org/10.1002/smi.1290>

Puri, P., Kaur, T., & Mehta, M. (2011). *Manual for stress scale (for students)*. Agra Psychological Research Cell.

Rajagopalan, A., Krishna, A., & Mukkadan, J. K. (2023). Effect of Om chanting and Yoga Nidra on depression anxiety stress, sleep quality and autonomic functions of hypertensive subjects – a randomized controlled trial. *Journal of Basic and Clinical Physiology and Pharmacology*, *34*(1), 69-75. <https://doi.org/https://doi.org/10.1515/jbcpp-2022-0122> (Journal of Basic and Clinical Physiology and Pharmacology)

Rajesh, S., Sathiyabama, N., & Kumaran, S. S. (2023). Yoga asana and yoga Nidra's different impacts on volleyball players anxiety levels. *International Journal of Research in Special Education*, *3*(1), 16-17.

Rani, K., Tiwari, S. C., Kumar, S., Singh, U., Prakash, J., & Srivastava, N. (2016). Psycho-biological changes with add on yoga nidra in patients with menstrual disorders: a randomized clinical trial. *Journal of caring sciences*, *5*(1), 1. <https://pmc.ncbi.nlm.nih.gov/articles/PMC4794540/pdf/jcs-5-1.pdf>

Rani, K., Tiwari, S. C., Singh, U., Agrawal, G. G., Ghildiyal, A., & Srivastava, N. (2011). Impact of Yoga Nidra on psychological general wellbeing in patients with menstrual irregularities: A randomized controlled trial. *International journal of yoga*, *4*(1), 20-25.

Rani, K., Tiwari, S. C., Singh, U., Singh, I., & Srivastava, N. (2012). Yoga Nidra as a complementary treatment of anxiety and depressive symptoms in patients with menstrual disorder. *International journal of yoga*, *5*(1), 52-56.

Rani, R., Kumar, A., & Sharma, P. (2013). Effect of yoga nidra on stress level among b. sc nursing first year students. *Nursing & Midwifery Research Journal*, *9*(2), 47-55.

Ravi, P., Boopalan, D., Manickam, A., Vijayakumar, V., & Kuppusamy, M. (2024). Yoga Nidra as an Adjunctive Therapy in Idiopathic Intracranial Hypertension: A Case Study. *Integrative and Complementary Therapies*, *30*(4), 166-169.

Schumann, D., Langhorst, J., Dobos, G., & Cramer, H. (2018). Randomised clinical trial: yoga vs a low‐FODMAP diet in patients with irritable bowel syndrome. *Alimentary pharmacology & therapeutics*, *47*(2), 203-211.

Sharpe, E., Butler, M., Hanes, D., & Ryan, B. (2021). 241 Remotely Delivered Yoga Nidra for Insomnia and Anxiety during COVID-19. *Sleep*, *44*, A96.

Sharpe, E., Butler, M. P., Clark-Stone, J., Soltanzadeh, R., Jindal, R., Hanes, D., & Bradley, R. (2023). A closer look at yoga nidra- early randomized sleep lab investigations. *Journal of Psychosomatic Research*, *166*, 111169. <https://doi.org/https://doi.org/10.1016/j.jpsychores.2023.111169>

Shivaji, P., & Dnyeshwar, P. (2025). Efficacy of Yoga-Nidra on Anxiety among Elderly People in Selected Old Age Homes. *Journal of Neonatal Surgery*, *14*, 20. <https://doi.org/10.52783/jns.v14.1427>

Singh, V., Krishna, N. R., Bhutia, T. N., & Singh, H. (2022). Effects of virtual iRest Yoga Nidra programme on depression, anxiety, and stress of sedentary women during the second outbreak of COVID-19. *Journal of Positive School Psychology*, *6*(3), 3716-3722.

Singh, V. K., & Adhikari, R. (2016). Impact of Yoga Practices on Occupational Stress among Indian Army Personnel. *International Journal of Yoga and Allied Sciences*, *5*(2), 108-112.

Stankovic, L. (2011). Transforming trauma: a qualitative feasibility study of integrative restoration (iRest) yoga Nidra on combat-related post-traumatic stress disorder. *International Journal of Yoga Therapy*, *21*(1), 23-37.

Sullivan, M., Lopez, S., Nault, D., Moonaz, S., & Miller, R. (2021). Yoga meditation for active duty military members with post-traumatic stress disorder: Results and discussion of a landmark initial study. *The Journal of Alternative and Complementary Medicine*, *27*(6), 522-524. <https://www.liebertpub.com/doi/10.1089/acm.2020.0466?url_ver=Z39.88-2003&rfr_id=ori%3Arid%3Acrossref.org&rfr_dat=cr_pub++0pubmed>

També, S. B. (2016). *OM–Die Ursprache der Seele: Durch Klang und Meditation das Gehirn neu programmieren–auf Heilung, Glück und das höchste Selbst-Mit Musik und Chants für das Meditationsprogramm*. arkana.

Tanna, K., & Khatri, S. (2024). Effect of Yoga Nidra on Perceived Stress in Individuals with High Blood Pressure: A Quasi-experimental Study. *Journal of Clinical & Diagnostic Research*, *18*(1).

Tripathi, R. C. (2018). *Effects of Effects of Yoga Nidra Yoga Nidra on Physical and Psychological Health* Venture into cross-cultural psychology: Proceedings from the 23rd Congress of the International Association for Cross-Cultural Psychology,

Vaishnav, B. S., Vaishnav, S. B., Vaishnav, V. S., & Varma, J. R. (2018). Effect of Yoga-nidra on Adolescents Well-being: A Mixed Method Study. *International journal of yoga*, *11*(3). <https://journals.lww.com/ijoy/fulltext/2018/11030/effect_of_yoga_nidra_on_adolescents_well_being__a.12.aspx>

Varma, P., & Khan, W. (2018). Efficacy of yoga and meditation in managing hassles and anxiety among angina pectoris patients. *Indian Journal of Community Psychology*, *14*(1).

Vijay, C., & Pal, R. (2023). The Efficacy of Yoga Nidra on Stress, Anxiety, and Aggression Levels in School-Going Children. *Int J Clin Exp Physiol*, *10*(2), 36-39.

Wahbeh, H., & Nelson, M. (2019). iRest meditation for older adults with depression symptoms: A pilot study. *International Journal of Yoga Therapy*, *29*(1), 9-17. <https://doi.org/http://doi.org/10.17761/2019-00036>

Yadav, M., & Sardar, S. (2016). Comparative effect of yoga asana and yoga Nidra on the anxiety level of inter collegiate level football players. *International Journal of Physical Education, Sports and Health*, *3*(2), 148-150.
