# Supplementary material for: Effects of Yoga Nidra on Stress, Anxiety, and Depression: A Systematic Review and Meta‐Analysis
Source: Ann N Y Acad Sci. 2025 Dec 1;1556(1):e70149. doi: 10.1111/nyas.70149 (PMC12917937; doi:10.1111/nyas.70149)
Supplement: Supplementary file 2 — Supplementary material: nyas70149‐sup‐0002‐SuppMat.pdf [file NYAS-1556-0-s002.pdf]

Supplementary file 2

RoB2 assessment

Legend	
Y	Yes
N	No
PY	Probably yes
PN	Probably no
NI	No information
NA	Not applicable

Domain 1

Study ID	1.1: Was the allocation sequence random?	1.2: Was the allocation sequence concealed until participants were enrolled and assigned to interventions?	1.3: Did baseline differences between intervention groups suggest a problem with the randomization process?	1.0 Algorithm's result	1.0 Assessor's judgement
Schumann et al. (2018)	Y	PY	PN	Low	Low
Barbuto (2017)	Y	NI	NI	Some concerns	Some concerns
Rani et al. (2016)	Y	NI	NI	Some concerns	Some concerns
Yadav and Sardar (2016)	NI	NI	PN	Some concerns	Some concerns
Deuskar	NI	PN	NI	High	High
K. (2008)	NI	NI	PN	Some concerns	Some concerns

Wahbeh and Nelson (2019)	NI	PY	N	Low	Low
Rajagopalan et al. (2023)	Y	Y	N	Low	Low
Sharpe et al. (2023)	NI	Y	N	Low	Low
Moszeik et al. (2025)	NI	NI	NI	Some concerns	Some concerns
Ferreira-Vorkapic et al. (2018)	Y	PY	PN	Low	Low
Muley et al. (2024)	NI	NI	NI	Some concerns	Some concerns
Rajesh et al. (2023)	NI	NI	NI	Some concerns	Some concerns
Vijay and Pal (2023)	Y	NI	NI	Some concerns	Some concerns
Nuzhath et al. (2024)	Y	NI	NI	Some concerns	Some concerns
Gunjiganvi et al. (2023)	Y	NI	NI	Some concerns	Some concerns
Moszeik et al. (2022)	Y	NI	NI	Some concerns	Some concerns
Jaiganesh et al. (2022)	Y	NI	NI	Some concerns	Some concerns
D’cunha et al. (2021)	Y	NI	NI	Some concerns	Some concerns
Rani et al. (2012)	Y	NI	PN	Some concerns	Some concerns
Rani et al. (2011)	Y	NI	PN	Some concerns	Some concerns
D'Souza et al. (2021)	Y	NI	N	Some concerns	Some concerns

Domain 2

Study ID	2.1: Were participants aware of their assigned intervention during the trial?	2.2: Were carers and people delivering the interventions aware of participants' assigned intervention during the trial?	2.3: Were there deviations from the intended intervention that arose because of the trial context?	2.4: Were these deviations likely to have affected the outcome?	2.5: Were these deviations from intended intervention balanced between groups?	2.6: Was an appropriate analysis used to estimate the effect of assignment to intervention?	2.7: Was there potential for a substantial impact (on the result) of the failure to analyse participants in the group to which they were randomized?	2.0 Algorithm's result	2.0 Assessor's judgement
Schumann et al. (2018)	PY	PY	PN	NA	NA	Y	NA	Low	Low
Barbuto (2017)	PY	PY	PN	NA	NA	NI	NI	High	High
Rani et al. (2016)	Y	Y	NI	NA	NA	Y	NA	Some concerns	Some concerns
Yadav and Sardar (2016)	Y	Y	NI	NA	NA	PY	NA	Some concerns	Some concerns
Deuskar	PY	PY	PN	NA	NA	NI	NI	High	High
K. (2008)	PY	PY	NI	NA	NA	PY	NA	Some concerns	Some concerns
Wahbeh and Nelson (2019)	Y	NI	PN	NA	NA	PY	NA	Low	Low
Rajagopalan et al. (2023)	PN	NI	NI	NA	NA	NI	NI	High	High

Sharpe et al. (2023)	PY	N	NI	NA	NA	Y	NA	Some concerns	Some concerns
Moszeik et al. (2025)	Y	NI	NI	NA	NA	Y	NA	Some concerns	Some concerns
Ferreira-Vorkapic et al. (2018)	PY	PY	NI	NA	NA	PY	NA	Some concerns	Some concerns
Muley et al. (2024)	Y	PY	NI	NA	NA	NI	NI	High	High
Rajesh et al. (2023)	PY	PY	NI	NA	NA	PY	NA	Some concerns	Some concerns
Vijay and Pal (2023)	PY	PY	NI	NA	NA	PY	NA	Some concerns	Some concerns
Nuzhath et al. (2024)	PY	PY	NI	NI	NI	PY	NA	Some concerns	Some concerns
Gunjiganvi et al. (2023)	PY	NI	NI	NA	NA	NI	NI	High	High
Moszeik et al. (2022)	PY	PY	NI	NA	NA	Y	NA	Some concerns	Some concerns
Jaiganesh et al. (2022)	PY	PY	NI	NA	NA	PY	NA	Some concerns	Some concerns
D’cunha et al. (2021)	Y	Y	PN	NA	NA	PY	NA	Low	Low
Rani et al. (2012)	PY	PY	NI	NA	NA	PY	NA	Some concerns	Some concerns
Rani et al. (2011)	PY	PY	PN	NA	NA	NI	NI	High	High
D'Souza et al. (2021)	PY	PY	PN	NA	NA	NI	NI	High	High

Domain 3

Study ID	3.1: Were data for this outcome available for all, or nearly all, participants randomized?	3.2: Is there evidence that the result was not biased by missing outcome data?	3.3: Could missingness in the outcome depend on its true value?	3.4: Is it likely that missingness in the outcome depended on its true value?	3.0 Algorithm's result	3.0 Assessor's judgement
Schumann et al. (2018)	N	Y	NA	NA	Low	Low
Barbuto (2017)	N	PN	NI	NI	High	High
Rani et al. (2016)	Y	NA	NA	NA	Low	Low
Yadav and Sardar (2016)	Y	NA	NA	NA	Low	Low
Deuskar	NI	N	NI	NI	High	High
K. (2008)	PY	NA	NA	NA	Low	Low
Wahbeh and Nelson (2019)	Y	NA	NA	NA	Low	Low
Rajagopalan et al. (2023)	N	N	NI	NI	High	High
Sharpe et al. (2023)	Y	NA	NA	NA	Low	Low
Moszeik et al. (2025)	N	Y	NA	NA	Low	Low
Ferreira-Vorkapic et al. (2018)	Y	NA	NA	NA	Low	Low
Muley et al. (2024)	Y	NA	NA	NA	Low	Low
Rajesh et al. (2023)	Y	NA	NA	NA	Low	Low
Vijay and Pal (2023)	Y	NA	NA	NA	Low	Low
Nuzhath et al. (2024)	Y	NA	NA	NA	Low	Low
Gunjiganvi et al. (2023)	N	PN	NI	NI	High	High

Moszeik et al. (2022)	N	PY	NA	NA	Low	Low
Jaiganesh et al. (2022)	Y	NA	NA	NA	Low	Low
D’cunha et al. (2021)	Y	NA	NA	NA	Low	Low
Rani et al. (2012)	Y	NA	NA	NA	Low	Low
Rani et al. (2011)	N	PN	NI	NI	High	High
D'Souza et al. (2021)	N	PN	NI	NI	High	High

Domain 4

Study ID	4.1: Was the method of measuring the outcome inappropriate?	4.2: Could measurement or ascertainment of the outcome have differed between intervention groups?	4.3: Were outcome assessors aware of the intervention received by study participants?	4.4: Could assessment of the outcome have been influenced by knowledge of intervention received?	4.5: Is it likely that assessment of the outcome was influenced by knowledge of intervention received?	4.0 Algorithm's result	4.0 Assessor's judgement
Schumann et al. (2018)	N	PN	N	NA	NA	Low	Low
Barbuto (2017)	N	PN	PY	NI	NI	High	High
Rani et al. (2016)	N	N	N	NA	NA	Low	Low
Yadav and Sardar (2016)	N	NI	PY	PY	PY	High	High
Deuskar	N	PN	PY	PY	PY	High	High
K. (2008)	N	PN	PY	PY	PY	High	High
Wahbeh and Nelson (2019)	N	PN	N	NA	NA	Low	Low
Rajagopalan et al. (2023)	N	N	NI	PY	PY	High	High
Sharpe et al. (2023)	N	PN	N	NA	NA	Low	Low

Moszeik et al. (2025)	N	N	PY	PY	PN	Some concerns	Some concerns
Ferreira-Vorkapic et al. (2018)	N	N	PY	NI	NI	High	High
Muley et al. (2024)	N	NI	NI	PY	NI	High	High
Rajesh et al. (2023)	N	PN	NI	PY	NI	High	High
Vijay and Pal (2023)	N	PN	PY	PY	NI	High	High
Nuzhath et al. (2024)	N	NI	NI	PY	NI	High	High
Gunjiganvi et al. (2023)	N	PN	NI	PY	NI	High	High
Moszeik et al. (2022)	N	N	PY	PY	NI	High	High
Jaiganesh et al. (2022)	N	PN	NI	PY	NI	High	High
D’cunha et al. (2021)	PN	PN	Y	NI	NI	High	High
Rani et al. (2012)	N	PN	PY	PY	PY	High	High
Rani et al. (2011)	N	PN	PY	NI	NI	High	High
D'Souza et al. (2021)	N	PN	NI	PY	NI	High	High

Domain 5:

Study ID	5.1: Were the data that produced this result analysed in accordance with a pre-specified analysis plan that was finalized before unblinded outcome data were available for analysis?	5.2: Is the numerical result being assessed likely to have been selected, on the basis of the results, from multiple eligible outcome measurements (e.g. scales, definitions, time points) within the outcome domain?	5.3: Is the numerical result being assessed likely to have been selected, on the basis of the results, from multiple eligible analyses of the data?	5.0 Algorithm's result	5.0 Assessor's judgement
Schumann et al. (2018)	NI	PN	PN	Some concerns	Some concerns
Barbuto (2017)	NI	PN	PN	Some concerns	Some concerns
Rani et al. (2016)	NI	PN	PN	Some concerns	Some concerns
Yadav and Sardar (2016)	NI	PN	PN	Some concerns	Some concerns
Deuskar	NI	PN	NI	Some concerns	Some concerns
K. (2008)	NI	PN	PN	Some concerns	Some concerns
Wahbeh and Nelson (2019)	NI	PN	PN	Some concerns	Some concerns
Rajagopalan et al. (2023)	NI	PN	N	Some concerns	Some concerns
Sharpe et al. (2023)	Y	N	N	Low	Low
Moszeik et al. (2025)	NI	N	N	Some concerns	Some concerns
Ferreira-Vorkapic et al. (2018)	NI	PN	PN	Some concerns	Some concerns
Muley et al. (2024)	NI	PN	PN	Some concerns	Some concerns
Rajesh et al. (2023)	NI	PN	PN	Some concerns	Some concerns

Vijay and Pal (2023)	NI	PN	PN	Some concerns	Some concerns
Nuzhath et al. (2024)	NI	PN	N	Some concerns	Some concerns
Gunjiganvi et al. (2023)	NI	PN	PN	Some concerns	Some concerns
Moszeik et al. (2022)	NI	PN	PN	Some concerns	Some concerns
Jaiganesh et al. (2022)	NI	PN	PN	Some concerns	Some concerns
D’cunha et al. (2021)	NI	PN	PN	Some concerns	Some concerns
Rani et al. (2012)	NI	PN	PN	Some concerns	Some concerns
Rani et al. (2011)	NI	PN	PN	Some concerns	Some concerns
D'Souza et al. (2021)	NI	PN	PN	Some concerns	Some concerns
